# Rediscovering Tomkins’ polarity theory: Humanism, normativism, and the psychological basis of left-right ideological conflict in the U.S. and Sweden

**DOI:** 10.1371/journal.pone.0236627

**Published:** 2020-07-31

**Authors:** Artur Nilsson, John T. Jost

**Affiliations:** 1 Department of Behavioural Sciences and Learning, Linköping University, Linköping, Sweden; 2 Department of Psychology, Lund University, Lund, Sweden; 3 Department of Psychology, New York University, New York, NY, United States of America; Saint Peter's University, UNITED STATES

## Abstract

According to Silvan Tomkins’ polarity theory, ideological thought is universally structured by a clash between two opposing worldviews. On the left, a humanistic worldview seeks to uphold the intrinsic value of the person; on the right, a normative worldview holds that human worth is contingent upon conformity to rules. In this article, we situate humanism and normativism within the context of contemporary models of political ideology as a function of motivated social cognition, beliefs about the social world, and personality traits. In four studies conducted in the U.S. and Sweden, normativism was robustly associated with rightist (or conservative) self-placement; conservative issue preferences; resistance to change and acceptance of inequality; right-wing authoritarianism and social dominance orientation; system justification and its underlying epistemic and existential motives to reduce uncertainty and threat; and a lack of openness, emotionality, and honesty-humility. Humanism exhibited the opposite relations to most of these constructs, but it was largely unrelated to epistemic and existential needs. Humanism was strongly associated with preferences for equality, openness to change, and low levels of authoritarianism, social dominance, and general and economic system justification. We conclude that polarity theory possesses considerable potential to explain how conflicts between worldviews shape contemporary politics.

## Introduction: Personality and politics

“Ideology appears in many domains, but it is found in its purest form in those controversies which are centuries old, and which have never ceased to find true believers, whether the issue is joined in mathematics or in aesthetics or in politics. Over and over again, whether theorists address themselves to one or another of these domains, they appear to become polarized on the same issues.” (Silvan Tomkins, 1963, [1, p. 389])

There has been a significant revival of interest in the link between personality and politics [2–5]—a topic that once garnered tremendous attention in both psychology and political science [6–10]. Because of its methodological hegemony, most researchers of recent vintage have adopted the “Big Five” taxonomy of personality traits as an integrative framework for guiding research in this area [2, 5, 11–14]. That is, researchers have largely focused on the traits of openness, conscientiousness, extraversion, agreeableness, and emotional stability—and the extent to which they are correlated with social and political attitudes.

Despite considerable strengths, the Big Five model leaves out a number of theoretically rich aspects of personality that are associated with one’s personal worldview, such as goals, values, scripts, narratives, personal constructs, and philosophical assumptions about the world [15–18]. These omissions are significant insofar as such features of personality shape “the dynamics of [the individual’s] initial resonance to ideology, of [his or her] seduction by ideas, of disenchantment with ideas, of addiction to ideas,” as one of the great psychological theoreticians of the left and right, Silvan Tomkins [19, p. 73], characterized the broad subject matter of the “psychology of knowledge.”

In the early years of the 21^st^ century, a number of studies have built on earlier research programs, documenting useful and interesting connections between political ideology, on one hand, and various types of character traits [11], personal values [20], moral intuitions [21], and beliefs about the social world [22], on the other. At the same time, the scientific yield has been largely taxonomic. These research programs have generated lists of specific discrepancies between leftists and rightists (or liberals and conservatives), but they have not produced an integrative account of how personal worldviews might structure political ideology (and vice versa). As a result, they tend to miss broader patterns of meaning that give rise to left-right divergence in ideological sensibilities [18]. We seek to rectify this oversight by revisiting Tomkins’ [1, 19, 23] pioneering work on polarity theory, mainly because it offers a sweeping, ambitious, and yet detailed theoretical account of the worldviews that animate ideologies of the left and right.

### Polarity theory: Humanism vs. normativism

According to Tomkins’ [1, 19, 23, 24] polarity theory, ideological thought is structured universally by a clash between two opposing worldviews. On the left, a *humanistic* worldview seeks to uphold the dignity of the person, representing him or her as intrinsically good and valuable, as “the measure, an end in himself [or herself], an active, creative, thinking, desiring, loving force in nature” [1, p. 391]. On the right, a *normative* worldview represents the person as devoid of intrinsic goodness and value and able to “realize himself, attain his full stature only through struggle toward, participation in, conformity to a norm, a measure, an ideal essence basically prior to and independent of man” [1, p. 392].

From these opposing worldviews of humanism and normativism, Tomkins derived a wide range of psychological consequences, including a parallel set of left-right divergences with respect to: (1) *attitudes to affect*, namely openness, tolerance, and enthusiasm as opposed to uneasiness, restraint, and self-control; (2) *interpersonal attitudes*, namely unconditional love, respect, and warmth as opposed to punishment and respect that is contingent upon conformity and achievement; (3) *epistemology*, namely imagination, creativity, and excitement as opposed to discipline, observation, and the minimization of error; and (4) *political values*, namely the promotion of human rights and well-being as opposed to the maintenance of law and social order (see also [25]).

Polarity theory was developed, at least in part, on the basis of observations made by cultural anthropologists, philosophers, and political theorists about the cultural meanings associated with left- and right-wing ideologies in various countries [26] (see also [27]). According to Tomkins [1, p. 389], there is a “love affair” between the individual’s “feelings and ideas about feelings” and organized belief systems, such as political (or religious) ideologies. Over time, the love affair tends to strengthen, and the bonds between psychological and ideological forces are likely to tighten. Tomkins wrote that: “the fit need not at the outset be perfect, so long as there is a similarity between what the individual thinks and feels is desirable and the characteristics of [the ideology] sufficient to set the two entities into sympathetic coordination with each other” (p. 389).

To some degree, polarity theory informed theory and research in social, personality, and political psychology during the latter half of the 20^th^ century, but it never rose to a position of great prominence in these areas. This is unfortunate, given the richness and originality of Tomkins’ theorizing and the fact that it is unique in offering an integrative psychological account of how personal worldviews structure ideology [25, 28–30]. Although several contemporary psychological theories of ideology highlight the roles of values, moral convictions, and beliefs about the social world [20–22], these models are more parochial and less ambitious than Tomkins’ theory. Among other things, they fail to appreciate the connections across different worldview domains (such as art, values, science, philosophy, and politics) and the underlying structure of personality that gives rise to these connections. Tomkins’ approach is more promising because it points the way toward an integrative conception of worldviews by identifying underlying philosophical assumptions, personal constructs, and narrative scripts or schemas that help to explain *why* various aspects of personality and ideology are intertwined in the first place [16, 18, 25, 30, 31].

To capture the contrast between humanistic and normative orientations in multiple domains of life, Tomkins [32] constructed the *Polarity Scale* in which respondents were presented with 59 pairs of statements and asked to endorse one of the statements, both, or neither (reprinted in [33]). Over the years, the scale was methodologically refined [33, 34], converted into Likert-style format [30], and expanded to reliably measure several different facets of humanism and normativism [25]. Studies confirm that humanism and normativism—as conceptualized by Tomkins—do indeed form coherent but distinct worldviews [25, 30, 31, 34].

Despite these empirical advances, the polarity between humanism and normativism has been largely neglected in recent scholarship pertaining to ideological differences in thoughts, feelings, and behavior [2, 3, 5, 13, 20–22, 35–37]. A handful of studies have provided suggestive evidence that humanism and normativism are related to left-right ideology in ways that polarity theory would portend [30, 38–41]. Results suggest that humanism and normativism represent at least somewhat distinct psychological systems or worldviews (see also [25, 31]), and these contribute independently to the polarization of political attitudes along a left-right dimension of ideology. However, these studies have typically relied on small convenience samples, *ad hoc* measures of ideology, and (in some cases) suboptimal measures of humanism and normativism. Most of the studies were conducted several decades ago, so their relevance to understanding current affairs is unclear. To our knowledge, no studies have linked the constructs of humanism and normativism to variables specified by major contemporary models of ideology.

### Contemporary psychological models of political ideology

In the present research program, we sought to leverage Tomkins’ [19] framework to develop a theory-driven, integrative account of left-right ideological coherence (or “ideo-affective resonance”) between psychological and political factors (see also [42]). The hope is that such an account will eventually help to illuminate potential sources of ideological conflict between the left and the right—both within the person (ambivalence) and within society as a whole (polarization). In particular, we investigated the roles of humanistic and normative worldviews in lending meaning, structure, and significance to one’s ideological belief system.

The goal of this article is not to present a comprehensive theory of all political belief systems but rather to provide an account of the potential clashes between generalized worldviews on the left and right. Some scholars have argued that political ideology is not reducible to a single dimension [35, 43–45]. Nevertheless, the left-right distinction is an extremely prominent one that is readily observable in a wide range of historical, cultural, and political contexts [42], and the incompatibility between the left and the right is what Tomkins set out to explain ([28], p. 105).

From the perspective of polarity theory, a failure to appreciate the generally oppositional nature of worldviews associated with humanism (on the left) and normativism (on the right) could lead researchers to underappreciate the role of psychological factors in contributing to ideological conflict and polarization. For instance, Malka and Soto [37] propose a multidimensional scheme in which exposure to political communication “can potentially lead people to adopt attitudes in certain domains that are substantively unrelated, or even contrary, to their underlying dispositional needs” and argue that “the ideological structuring of political attitudes… might not cohere, and might even sometimes compete, with the structuring compelled by dispositional influences” (p. 140; see also [35]). One limitation of approaches such as this one is that they have little to offer when it comes to understanding the intensity and precise nature of left-right conflict in society (e.g., [46], see also [4]).

For Tomkins [23], on the other hand, the left-right conflict is a fundamental, age-old one that is “a sublimated derivative of social stratification and exploitation” (p. 173). This is because social systems based on stratification and exploitation—as most social systems throughout human history have been [47]—are sure to have both defenders and challengers. Normative, right-wing ideologies are “defensive ideologies [that] vary as a function of the nature of the society they defend” and “place the blame for [problems in society] squarely upon those who suffer and complain,” like “the welfare ‘cheats’ who are to blame for their own problems” ([23], p. 176). By contrast, humanistic, left-wing ideologies “place the blame for the problematic on the established normative authority, which must then change itself or be changed by those who suffer” (p. 177).

This formulation is highly compatible with Jost et al.’s [15] model of ideology as motivated social cognition, which proposes that leftists are motivated to increase social, economic, and political equality and this leads them to be supportive of social change, whereas rightists are motivated to preserve tradition and to maintain the status quo and are therefore prepared to justify existing forms of inequality. Or, as Tomkins [23] put it: “the left represented, then [hundreds of years ago] as now, the oppressed and exploited against their warrior oppressors” (p. 173), and “the right is… apologist of primarily masculine, adversarial stratification, buttressed by ‘tradition’” (p. 177). In an effort to synthesize insights from polarity theory and the model of ideology as motivated social cognition, we hypothesized that humanism would be associated with rejection (vs. acceptance) of inequality and advocacy of (vs. resistance to) social change, whereas normativism would be associated with acceptance (vs. rejection) of inequality and resistance to (vs. advocacy of) social change.

Relatedly, we considered the role of system justification (see also [39])—defined as the motivation to defend, bolster, and justify the societal status quo [48], which is itself thought to be undergirded by epistemic and existential needs to reduce uncertainty and threat [49]. We hypothesized that humanism would be negatively associated with system justification, epistemic needs, and existential needs and that normativism would be positively associated with these motivational dispositions. These hypotheses are consistent with Tomkins’ [23, p. 175–7] proposal that normative, right-wing ideologies are in defense of the system of social stratification, whereas humanistic, left-wing ideologies are offered in protest against systems that are perceived as unjust or exploitative [50].

We also incorporated key variables from Duckitt’s [22] dual process model, which is another prominent perspective in contemporary psychology. This model suggests that there are two distinct motivational systems that underlie right-wing ideology. One of these is associated with Right-Wing Authoritarianism (RWA) and is related to social conformity and the belief that the world is a dangerous place, in which the lives of good people are threatened by the actions of bad people. The other is associated with Social Dominance Orientation (SDO) and is related to tough-mindedness and the belief that the world is a competitive jungle, characterized by a ruthless struggle for resources and power. These ideas, too, are broadly consistent with Tomkins’ theorizing, insofar as the person who is drawn to right-wing ideology is described as “negatively disposed toward human beings in his displayed affect, in his perceptions, and in his cognitions” [23, p. 171] and as holding that “the weak should be toughened and the strong willed should be curbed” [1, p. 410].

Finally, we sought to integrate contemporary models of personality psychology. Research inspired by the Big Five model has demonstrated that openness to new experiences is associated with liberal and left-wing ideology, whereas conscientiousness is associated with conservative and right-wing ideology [11, 13, 20]. Chirumbolo and Leone [51] have suggested that the HEXACO six-factor model, which modifies and extends the Big Five, may be preferable to the Big Five when it comes to the study of ideology. The HEXACO includes a sixth trait, referred to as honesty-humility, which covers sincerity, fairness, modesty, and lack of materialism. The other five traits have meanings that differ subtly from the Big Five. Notably, neuroticism is called emotionality within the HEXACO, where it includes empathy, emotional ties to others, and sentimentality, in addition to anxiety [52]. Past research suggests that honesty-humility and emotionality are associated with low social dominance [53, 54].

## Overview of research and hypotheses

The purpose of this research program was to systematically investigate relations between humanism and normativism, on one hand, and variables that play key roles in contemporary models of political ideology, on the other. To head off possible misunderstandings, our goal was not to test a *causal* model of the congruence between personality and political orientation. Rather, we explored the possibility that incorporating the constructs of humanism and normativism would help to elucidate associations among seemingly disparate phenomena—such as epistemological orientations, beliefs about human nature and the social world, political attitudes, and personality traits (see also [31]). In so doing, we hoped to extend and elaborate upon of the existing body of knowledge concerning the personality-ideology interface.

We see this attempt at unification as a crucial aspect of scientific explanation [55, 56], perhaps especially in the social sciences, where the testing of truly comprehensive causal models is seldom feasible [57]. Approaches such as this one can, however, serve as a springboard for future empirical investigations of causal mechanisms using experimental or longitudinal designs as well as enriched hermeneutic understandings of human experience. In contrast to many other theories of political ideology, polarity theory offers a sophisticated, integrative account of generalized worldviews that specifies core values and philosophical assumptions about humanism and normativism that can help to explain broader patterns of coherence and structure in both personality and ideology [18, 25, 30, 31].

It is too early in the research process to state with confidence whether humanism and normativism should be thought of as “master constructs” that help the individual to organize many other traits and characteristics of social and political significance. Nonetheless, if Tomkins was correct that these poles reflect fundamental elements of the human experience and that they shape the individual’s personality and his or her overall worldview, they should be linked to a wide range of political and psychological outcomes. Our main predictions, which were derived from the foregoing theoretical analysis, are enumerated as follows:

(H1) Humanism will be (a) associated with leftist (and liberal) ideological self-placements and preferences, whereas normativism will be (b) associated with rightist (and conservative) ideological self-placement and preferences.
(H2) Humanism will be associated with (a) a preference for equality, openness to social change, lesser system justification, and weaker epistemic and existential needs to reduce uncertainty and threat. Normativism will be associated with (b) a tolerance of inequality, resistance to change, greater system justification, and stronger epistemic and existential needs to reduce uncertainty and threat.
(H3) Humanism will be negatively associated with (a) competitive-world beliefs, SDO, dangerous-world beliefs, and RWA. Normativism will be positively associated with (b) competitive-world beliefs, SDO, dangerous-world beliefs, and RWA.
(H4) Humanism will be (a) positively associated with openness, emotionality, and honesty-humility, whereas normativism will be (b) negatively associated with openness, emotionality, and honesty-humility.

We tested these hypotheses in four studies. In Study 1, we investigated the associations between humanism and normativism, on one hand, and political orientation, on the other, in the context of Jost et al.’s [15] model of ideology as motivated social cognition and Duckitt’s [22] dual process model of ideology. Study 2 was a conceptual replication of Study 1 using a more diverse U.S. sample and an issue-based measure of ideological preferences. In Studies 3 and 4, we investigated the same theoretical models in the context of Sweden. In Study 4, we incorporated the HEXACO framework [52] as well.

Although the U.S. and Sweden are both Western, post-industrial democracies, there are critical differences between them. Sweden has a long history of Social-Democratic rule, and the left-right dimension is primarily structured according to attitudes that are supportive or critical of the economic system, which may be characterized as “welfare capitalism” [44, 58, 59]. Given this social and historical context, it is at least conceivable that system justification tendencies and resistance to change would be more prevalent among Social Democrats (on the Swedish left) than among “Liberals” and libertarians (on the Swedish right)—as Jost, Federico, and Napier [60] suggested. It may also be worth noting that Sweden is one of the most egalitarian nations in the world in terms of income and gender disparities, whereas the U.S. is one of the least egalitarian Western nations [61]. In addition, Sweden is one of the most secular countries in the world, and the U.S. is the least secular country in the West [62]. Despite these differences, it is clear that Tomkins’ ambitious theorizing seeks to address truly universal themes pertaining to ideology. Therefore, we did not make separate predictions for the two countries.

## Study 1

In this study, we investigated associations between humanism and normativism, on one hand, and variables that are central to two psychological models of ideology, on the other, namely Jost et al.’s [15] model of ideology as motivated social cognition and Duckitt’s [22] dual process model of ideology, in a sample of US undergraduate students. We also conducted an exploratory *post hoc* analysis of correlations across the different facets of humanism and normativism to address the possibility that humanistic and normative values would be associated with left-right political orientation.

### Method

This research was approved by New York University’s Committee on Activities Involving Human Subjects (Protocol #09–7585). Participants were given full disclosure of the procedure and their rights, they were not subjected to an experimental manipulation, and their data were completely anonymous. We obtained written consent from all participants and offered them both written and verbal forms of debriefing. All relevant data are within the manuscript and its Supporting Information Files. Data files and other supplementary documents are also are accessible through the Open Science Framework: https://osf.io/crjus.

#### Participants

Participants were 384 New York University psychology students who were compensated with course credit (mean age = 19.6, *SD* = 1.62; 77.3% women). Of these, 116 participated in a laboratory and 268 accessed the *Psychsurveys*.*org* website. Ideological self-placement was left-of-center for 69.8% and right-of-center for 9.64% of the participants. Scales measuring resistance to change, preference for equality, general system justification, and need for closure were part of a separate test battery completed by 199 of the participants (mean age = 19.1, *SD* = 1.40, 73.0% women). The items measuring ideological self-placement in terms of social and economic issues were completed by participants who took the study online (mean age = 19.6, *SD* = 1.62, 66.4% women).

To ensure adequate power, we decided to use sample sizes of 200 persons at a minimum and preferably more than 300 persons, in line with common recommendations for sampling in structural equation modeling [63] and correlation analysis [64]. At the same time, it is important to note that simulation studies have shown that relations among sample size, power, and bias in structural equation models can vary substantially depending upon model characteristics. Wolf, Harrington, Clark, and Miller [65] found that models with weak (or extremely strong) factor loadings and paths required larger sample sizes to ensure adequate power and unbiased parameter estimates, but increasing the number of latent factors above two had little effect on the minimum sample size required. Our models generally had relatively strong factor loadings, albeit paths of varying magnitude, and our sample sizes were much closer to the upper end (*N* = 460) than the lower end (*N* = 30) of minimum sample sizes reported by Wolf et al. [65]. Calculations of a posteriori power for RMSEA [66] yielded estimates close to 100% for all of our models, and this held true across all of our studies. Calculations of a posteriori power for correlation analysis [67] revealed that our sample sizes gave us at least 80% power (two-tailed) to detect associations of |*r*| = .15 (*n* = 384) and .20 (*n* = 199). Because of missing data, the actual sample sizes were slightly smaller for some of the variables (*n* ≥ 371 in the full sample and *n* ≥ 196 in the subsample) but this did not alter the power estimates.

#### Measures

We used Likert response scales, which were anchored by “*Strongly disagree*” and “*Strongly agree*” for all variables. All items are available in (S1 Appendix).

We measured humanism (*M* = 5.27, *SD* = .55; *α* = .90) and normativism (*M* = 3.67, *SD* = .62; *α* = .90) with 40 items each. These items were distributed equally among five facet-domains: view of human nature (*α*_humanism_ = .76, *α*_normativism_ = .73), interpersonal attitude (*α*_humanism_ = .76, *α*_normativism_ = .73), attitude to affect (*α*_humanism_ = .76, *α*_normativism_ = .77), epistemology (*α*_humanism_ = .67, *α*_normativism_ = .66), and political values (*α*_humanism_ = .76, *α*_normativism_ = .75) [25, 68]. Sample items include “Human beings are basically good” (humanism) and “The maintenance of law and order is the most important duty of any government” (normativism). Participants who completed Study 1 in a laboratory context responded to the items measuring humanism and normativism on a 1–7 Likert scale while those who took this study online responded on a 1–5 Likert scale. We therefore transformed the responses of the latter group so that these scales would range from 1 to 7 for all participants in Study 1.

We measured resistance to change with 11 items (e.g. “If you start changing things very much, you often end up making them worse,” *M* = 4.87, *SD* = .97; *α* = .71) and preference for equality with 9 items (e.g., “Prosperous nations have a moral obligation to share some of their wealth with poor nations,” *M* = 6.43, *SD* = 1.17; *α* = .77). Most of the items were constructed or taken from scales that have undergone extensive data collection and refinement prior to the current research. We measured general system justification (*M* = 4.42, *SD* = 1.18; *α* = .76) with Kay and Jost’s [69] scale (8 items, e.g. “Society is set up so that people usually get what they deserve”). Participants responded on a 1–9 Likert scale. We measured economic system justification (*M* = 2.96, *SD* = .91; *α* = .76) with 7 items taken from Jost and Thompson’s [70] scale (e.g. “Social class differences reflect differences in the natural order of things”).

We measured existential motivation with four items (e.g. “I try to have nothing to do with the subject of death”) addressing death anxiety [71] (*M* = 4.02, *SD* = 1.34; *α* = .80), a short measure (12 items, e.g. “I try to avoid getting too close to my partner”) of insecure romantic relationship attachment [72] (*M* = 3.55, *SD* = .75; *α* = .76), and one item addressing fear of terrorism (“Our way of life is seriously threatened by the forces of terrorism in the world”, *M* = 4.04, *SD* = 1.50). Participants responded on a 1–7 Likert scale. We measured epistemic motivation (*M* = 3.64, *SD* = .48; *α* = .86) with Webster and Kruglanski’s [73] need for cognitive closure scale (42 items, e.g. “I enjoy having a clear and structured mode of life”); participants responded on a 1–6 Likert scale in this case.

We measured RWA (*M* = 3.31, *SD* = .63; *α* = .79) with a modified short-version [74] of Altemeyer’s [75] original scale (15 items, e.g. “There are many radical, immoral people trying to ruin things; the society ought to stop them”) that uses items with less extreme wording and less reference to specific social groups (e.g., women and homosexuals), compared to the original scale. We measured SDO (*M* = 3.04, *SD* = .90; *α* = .77) with a short-version of the SDO scale [47] (8 items, e.g. “Inferior groups should stay in their place”). We measured perceptions of a dangerous world (*M* = 3.56, *SD* = .74; *α* = .78) with Duckitt’s [22] modification of Altemeyer’s [76] scale (10 items, e.g. “Every day as society becomes more lawless and bestial, a person’s chances of being robbed, assaulted, and even murdered go up and up”). We measured belief in a competitive-jungle world (*M* = 2.94, *SD* = .87; *α* = .83) with Duckitt’s [22] scale (10 items, e.g. “If it’s necessary to be cold blooded and vengeful to reach one’s goals, then one should do it”). Participants responded on a 1–7 Likert scale.

Participants reported their ideological self-placement (“Where would you place yourself on the following scale of political orientation?”, *M* = 3.71, *SD* = 1.39; “In terms of economic issues, where would you place yourself on the following scale?”, *M* = 4.64, *SD* = 1.79; “In terms of social and cultural issues, where would you place yourself on the following scale?”, *M* = 3.28, *SD* = 1.67) on Likert scales ranging from 1 (*Extremely liberal*) to 9 (*Extremely conservative*), which have been used widely in previous research (e.g., [4, 11]).

Participants completed the measures in the following order: humanism and normativism; political attitudes (resistance to change, preference for equality, RWA, SDO, and system justification); dangerous- and competitive-world beliefs; existential and epistemic motivations; and ideological self-placement. Item order was randomized within all sections except for the last one.

#### Statistical procedure

We calculated correlations in SPSS 26.0 to elucidate the pattern of relations involving humanism, normativism, and other psychological constructs. We report results of two-tailed tests with an alpha level of 0.05. We used Holm’s [77] sequential Bonferroni procedure to adjust the significance threshold for the number of hypotheses tested in each study (for details, see https://osf.io/crjus).

We used structural equation modeling to investigate which ideological variables were most strongly and directly associated with humanism and normativism in relatively comprehensive models. A key advantage of this technique is that it introduces a measurement model that separates latent factors from measurement error, while also enabling a test of the assumption that the modeled variables are *de facto* factorially distinct. Because structural equation modeling is sometimes conflated with causal analysis [78], it important to keep in mind that causal assumptions are, like any other metaphysical or epistemological assumptions, not intrinsic to any particular statistical technique. With this work we were in no way attempting to test any causal assumptions about, for instance, the developmental sequence of political attitude acquisition in childhood and adolescence.

We ran structural equation modeling in AMOS 23.0 basing calculations upon the covariance matrix and the maximum likelihood method. We evaluated model fit in terms of the χ^2^ Goodness-of-fit test; the Comparative Fit Index (CFI), which estimates goodness of fit compared to the null model; and the Root Mean Square Error of Approximation (RMSEA), which estimates lack of fit in relation to a perfect model, with 90% confidence intervals. The CFI is less sensitive to changes in model parsimony vs. complexity than the RMSEA. Widely employed conventions suggest that CFI estimates above .95 and RMSEA estimates below .06 indicate acceptable model fit, but statistical simulation studies have revealed that it is difficult to specify absolute boundaries for model fit that hold up across all sample sizes and distributions of data [79].

To specify the indicators of each latent variable in our models, we used item parceling. This technique is helpful when the substantive research questions concern the relations among constructs rather than the behavior of items, by reducing irrelevant noise and the risk of estimation errors [80, 81]. A detailed description of the parceling procedure is available in (S1 Appendix).

**Fig 1:**
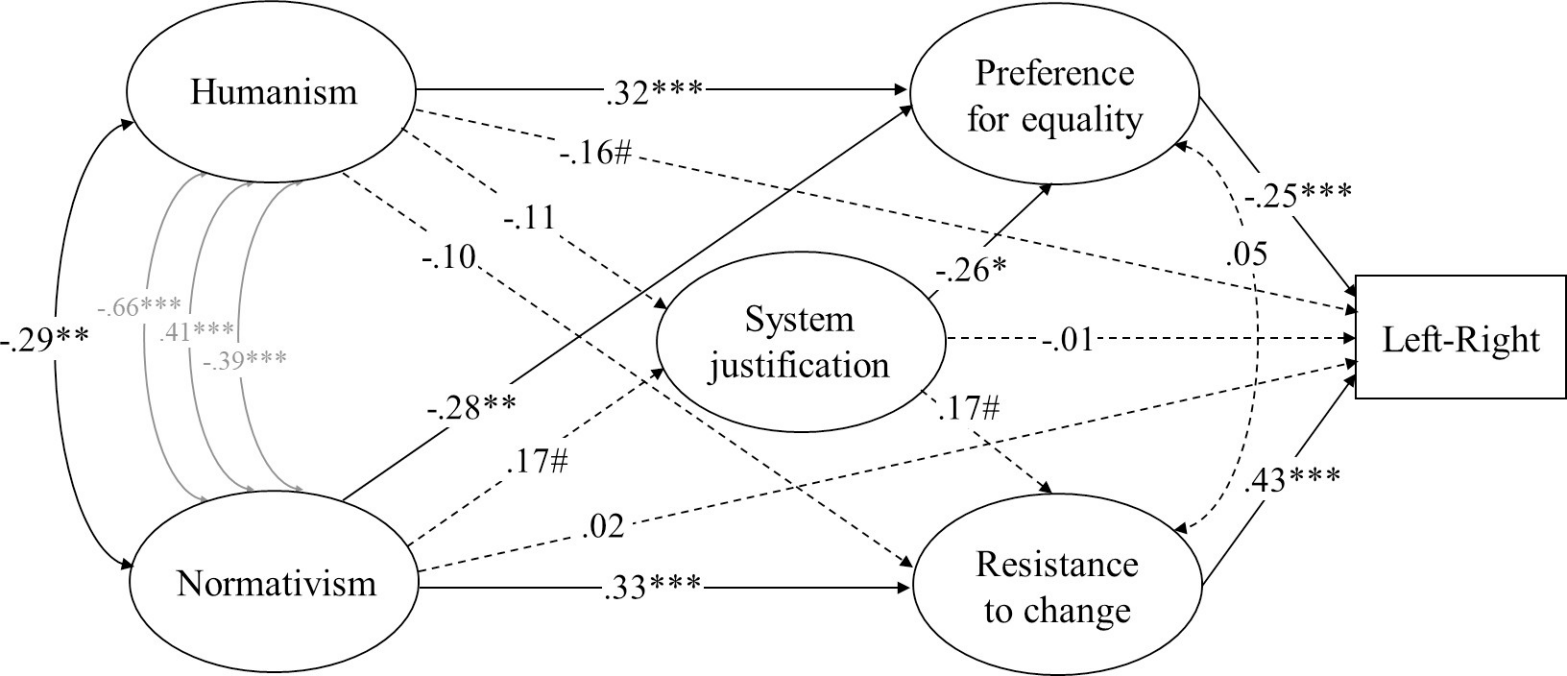
Structural equation model (standardized solution) of associations between humanist and normativist worldviews and the model of ideology as motivated social cognition in Study 1. # *p* < .10, * *p* < .05, ** *p* < .01, *** *p* < .001 (dotted lines represent non-significant estimates). Disturbances and factor loadings are not shown.

**Fig 2:**
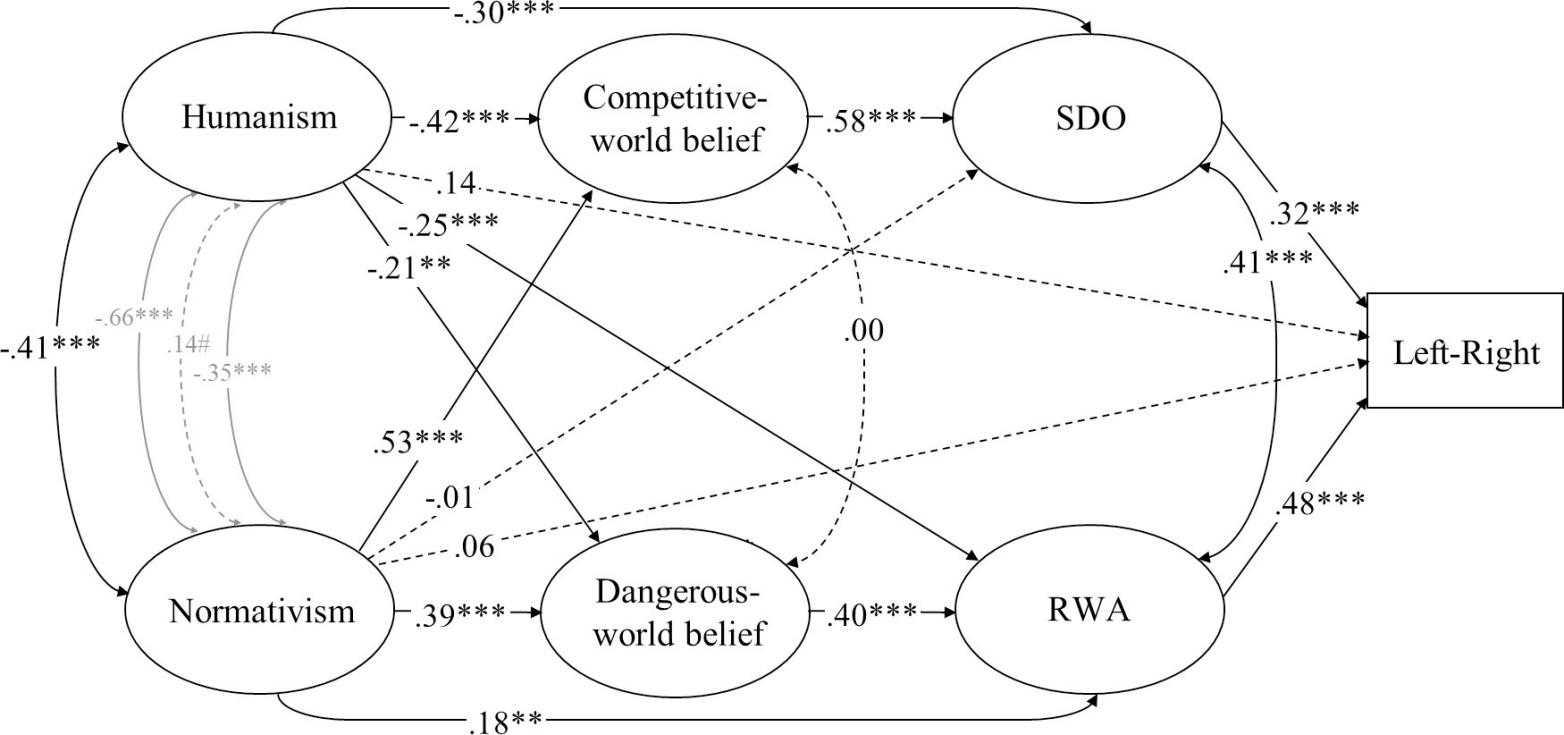
Structural equation model (standardized solution) of associations between humanist and normativist worldviews and constructs representing the dual process model of ideology in Study 1. # *p* < .10, * *p* < .05, ** *p* < .01, *** *p* < .001 (dotted lines represent non-significant estimates). Disturbances and factor loadings are not shown.

Because there is a degree of conceptual overlap among the variables, we first conducted confirmatory factor analyses in AMOS 23.0 and detailed item-level exploratory factor analyses in R 3.0 (the “psych” package) to ascertain the distinctness of overlapping variables. Models that represented each construct with separate factors did indeed generally yield better fit than models that integrated similar constructs, and distinct theoretical constructs (and their items) tended to load on different factors in exploratory factory analyses (see S2 Appendix). Furthermore, the omega total (*ω*_*t*_) reliability coefficient [82, 83], which provides an estimate of how much of the covariance between the indicators all of the latent factors in the model account for, was .88 for the full five-factor measurement model for ideology as motivated social cognition (Fig 1) and .93 for the full six-factor dual process measurement model (Fig 2) in this study. All factor loadings were strong (Humanism: *λ* ≥ .61; Normativism: *λ* ≥ .61; Resistance to change: *λ* ≥ .48; Preference for equality: *λ* ≥ .65; System justification: *λ* ≥ .57; RWA: *λ* ≥ .68; SDO: *λ* ≥ .72; Dangerous-world belief: *λ* ≥ .68; Competitive-world belief: *λ* ≥ .74).

**Fig 3:**
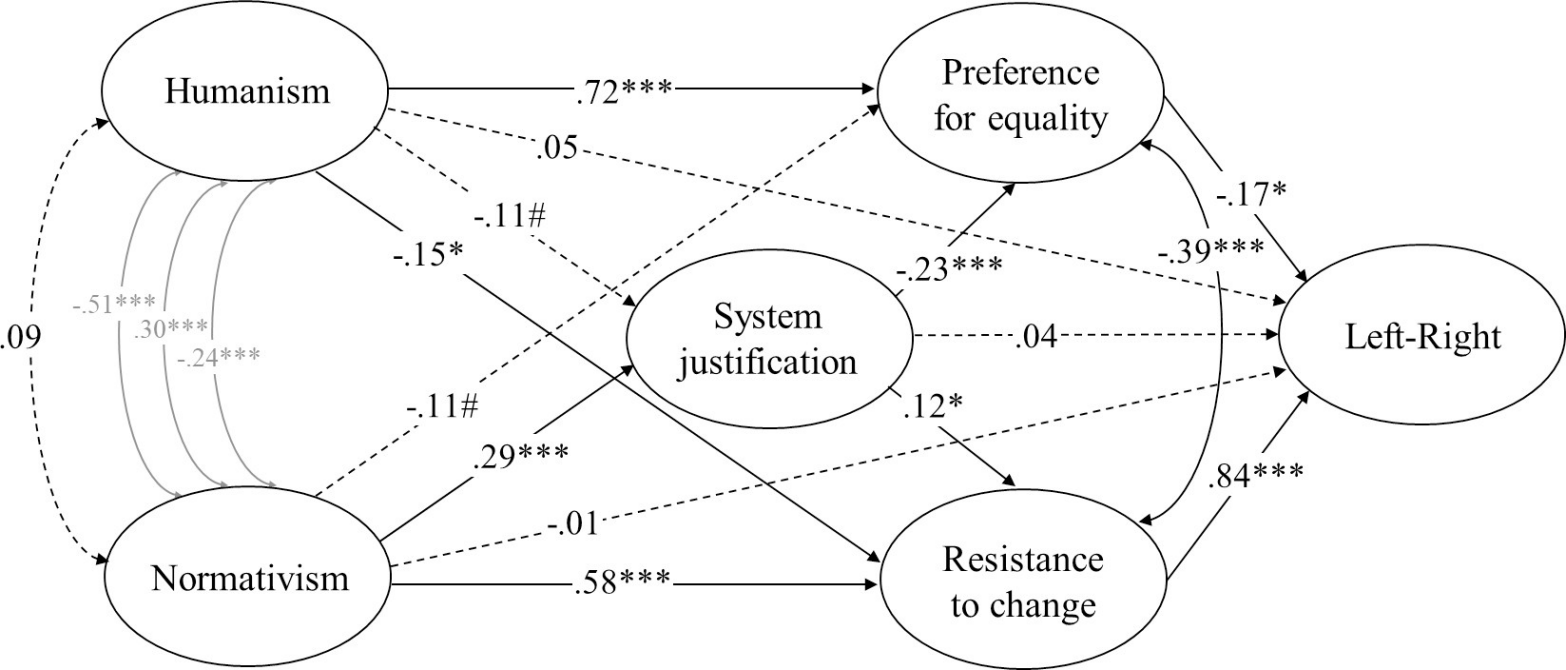
Structural equation model (standardized solution) of associations between humanist and normativist worldviews and the model of ideology as motivated social cognition in Study 2. # *p* < .10, * *p* < .05, ** *p* < .01, *** *p* < .001 (dotted lines represent non-significant estimates). Disturbances and factor loadings are not shown.

**Fig 4:**
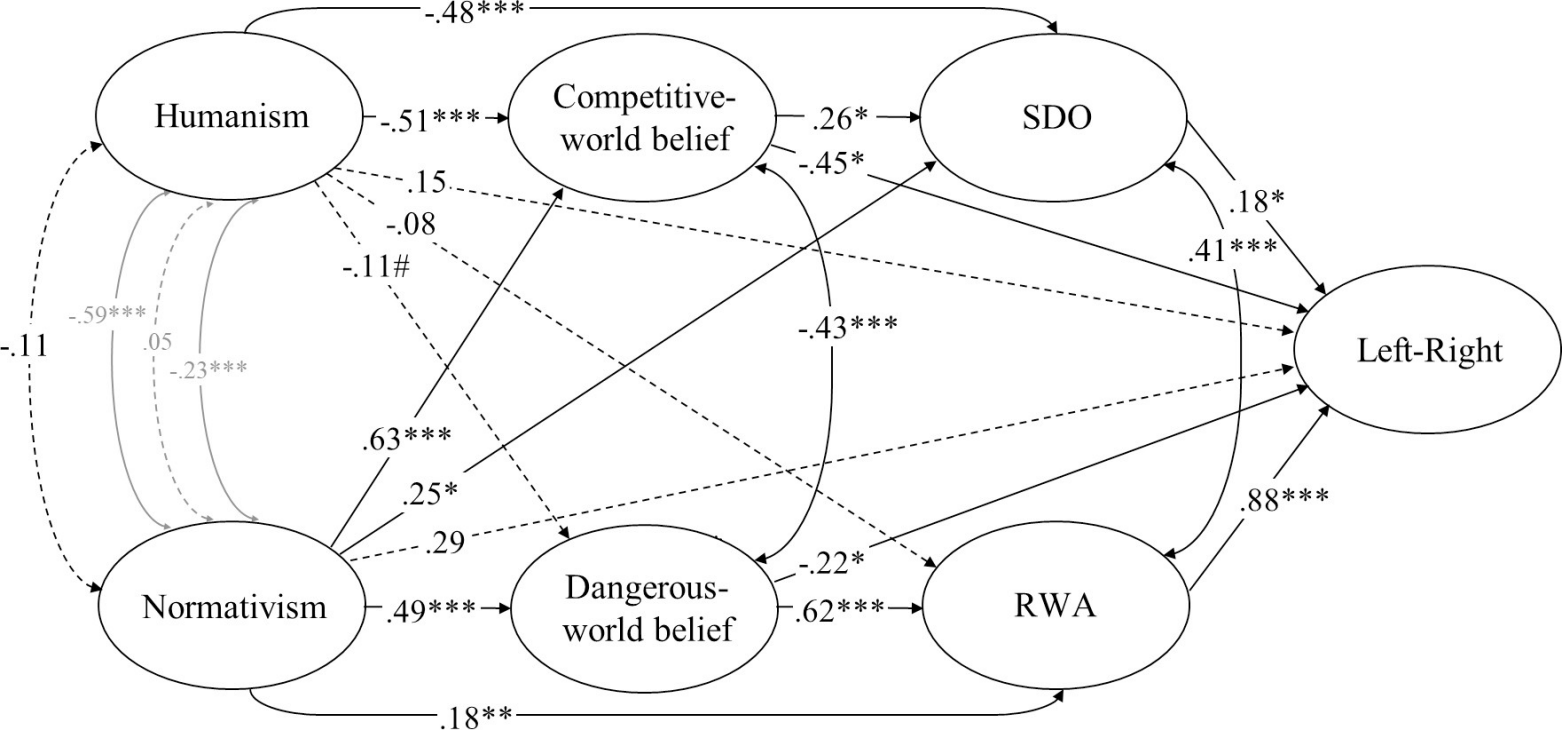
Structural equation model (standardized solution) of associations between humanist and normativist worldviews and constructs representing the dual process model of ideology in Study 2. # *p* < .10, * *p* < .05, ** *p* < .01, *** *p* < .001 (dotted lines represent non-significant estimates). Disturbances and factor loadings are not shown.

**Fig 5:**
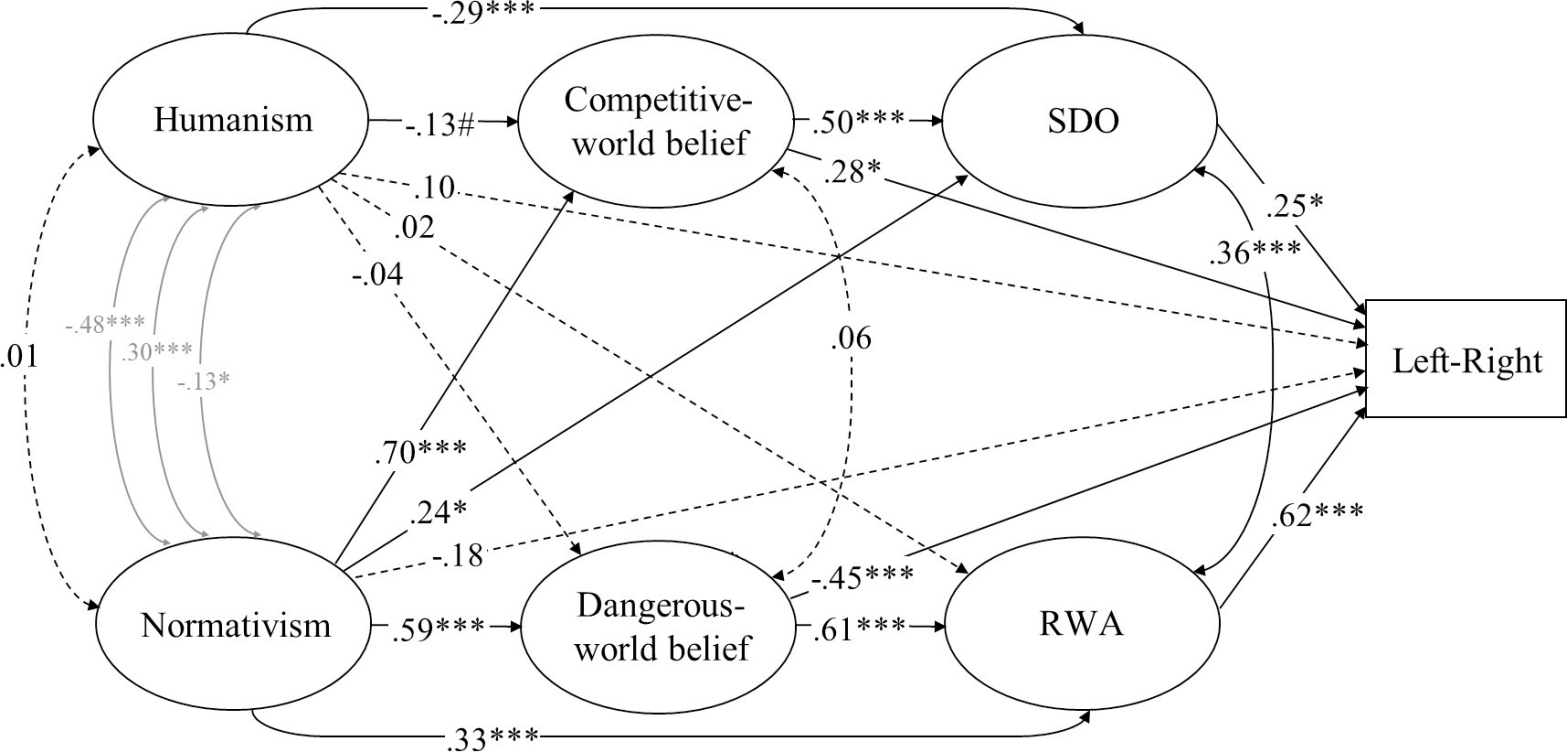
Structural equation model (standardized solution) of associations between humanist and normativist worldviews and constructs representing the dual process model of ideology in Study 3. # *p* < .10, * *p* < .05, ** *p* < .01, *** *p* < .001 (dotted lines represent non-significant estimates). Disturbances and factor loadings are not shown.

We designed complete structural equation models, which include a measurement model and a structural model, to elucidate the pattern of relations between humanistic and normative worldviews, on one hand, and political ideology, on the other—specifying the relations among variables on the basis of prior theory and research. We placed humanism and normativism first in our models because we were primarily interested in the extent to which they accounted for variance in the other constructs, rather than the other way around. We also considered plausible changes in the ordering of the variables in our models, but these changes turned out to yield mathematically equivalent models (see [84] for an explanation). In analyses addressing the model of political ideology as motivated social cognition, the paths from humanism and normativism to ideological self-placement were specified to be mediated by system justification, resistance to change, and preference for equality, and the path from system justification to ideological self-placement was specified to be mediated by resistance to change and preference for equality (Figs 1, 3 and 7). For the dual process model, the path from dangerous-world beliefs to ideological self-placement was specified to be mediated by RWA and the path from competitive-world beliefs to ideological self-placement was specified to be mediated by SDO, and the paths from humanism and normativism to ideological self-placement were specified to be mediated by these four variables (Figs 2, 4 and 5). We estimated the covariances of residual terms of theoretically coupled variables, including humanism and normativism, preference for equality and resistance to change, RWA and SDO, and dangerous- and competitive-world beliefs.

Model fit was acceptable both for the model of ideology as social cognition, *χ*^*2*^(87) = 148.87, *p* < .001, CFI = .935, RMSEA = .060 [.043, .076], and the dual process model, *χ*^*2*^(133) = 352.4, *p* < .001, CFI = .930, RMSEA = .066 [.057, .074]. We therefore made no post hoc adjustments to these models. We also explored the effect of excluding multivariate outliers from each analysis based on their Mahalanobis distance (*p* < .001, [85]), but we report the results without these exclusions because their effect on the results was negligible. We saw no signs of multicollinearity when comparing path estimates across model variations.

For those cases in which the paths from humanism (or normativism) to *both* resistance to change and acceptance of inequality (or RWA and SDO or dangerous-world and competitive-world beliefs) were significant, we compared the strengths of these paths by computing bias corrected 95% unstandardized bootstrap confidence intervals with 20000 resamples to test the differences between paths. All variables were standardized prior to these analyses. Indirect paths from humanism and normativism to political orientation through the constructs in our models are reported in (S3 Appendix).

### Results

Correlations bearing on our hypotheses are summarized in Table 1. Humanism was consistently associated with a more leftist (or liberal) self-placement in terms of general, social, and economic concerns, whereas normativism was consistently associated with a more rightist (or conservative) self-placement, providing unambiguous support for (H1a) and (H1b).

**Table 1:** Correlations involving humanism, normativism, ideology, and motivation in Study 1 (U.S.).

	1	2	3	4	5	6	7	8	9	10	11	12	13	14	15	16
1. Humanism																
2. Normativism	-.36***															
3. General ideological self-placement	-.29***	.37***														
4. Social ideology self-placement	-.24***	.24***	.72***													
5. Economic ideology self-placement	-.22***	.29***	.60***	.40***												
6. Resistance to change	-.19**	.36***	.46***	.46***	.21**											
7. Preference for equality	.39***	-.37***	-.36***	-.20*	-.50***	-.21**										
8. RWA	-.36***	.42***	.61***	.53***	.20***	.58***	-.12									
9. SDO	-.54***	.43***	.49***	.33***	.45***	.22**	-.59***	.46***								
10. Dangerous-world beliefs	-.38***	.51***	.39***	.32***	.18**	.46***	-.09	.58***	.37***							
11. Competitive-world beliefs	-.54***	.57***	.35***	.20***	.27***	.23**	-.42***	.35***	.63***	.43***						
12. General SJ	-.10	.23**	.25***	.27***	.40***	.25***	-.29***	.21**	.24**	-.10	.17*					
13. Economic SJ	-.32***	.47***	.44***	.35***	.45***	.36***	-.48***	.42***	.57***	.33***	.44***	.43***				
14. Need for closure	-.11	.29***	.24***	.41***	.16*	.31***	-.07	.28***	.14	.34***	.28***	.13	.17*			
15. Death anxiety	01	.12*	.06	.10	.17**	.07	.08	.10	.00	.02	.08	.05	.09	.16*		
16. Insecure attachment	.00	.22***	-.01	-.06	.02	.13	-.06	.00	.08	.07	.17**	-.14*	.04	.11	.16**	
17. Fear of terrorism	-.07	.22***	.25***	.30***	.18**	.40***	-.11	.29***	.12*	.35***	.06	.05	.21***	.26***	.16**	.06

* *p* < .05.

** *p* < .01.

*** *p* < .001.

#### The model of ideology as motivated social cognition

With respect to (H2a), humanism was associated with preference for equality, openness to change, and (low) economic system justification, but it was not associated with general system justification, epistemic motivation, or existential motivation. In summary, then, support for (H2a) was somewhat mixed. Support for (H2b) was much stronger. Normativism was significantly correlated with resistance to change, tolerance for inequality, economic system justification, and general system justification, and with all measures of epistemic and existential motivation, although the correlation between normativism and death anxiety failed to reach significance when we adjusted for multiple testing (*p* = .023; corrected *p*-threshold = .008).

As shown in Fig 1, the associations between humanism and leftist self-placement in general, openness to change, and system justification failed to reach significance once other variables were taken into account through structural equation modeling. Normativism, on the other hand, was directly and robustly associated with resistance to change and acceptance of inequality; the magnitude of these associations did not differ significantly (.08[-.20, .42], *p* = .61).

#### The dual-process model

Support for (H3) was unequivocal in this study. Humanism was strongly and negatively associated with RWA, SDO, and dangerous-world and competitive-world beliefs, consistent with (H3a). Furthermore, normativism was strongly and positively associated with these four variables, consistent with (H3b).

Structural equation modeling revealed that normativism was directly and robustly associated with RWA but not SDO after adjusting for all other variables, as illustrated in Fig 2. Normativism was also directly associated with both dangerous-world and competitive-world belief, and these associations were approximately equally strong (.10 [.06, .25], *p* = .21). Humanism was negatively associated with RWA and SDO to a similar degree (.07 [.18, .33], *p* = .54); it was slightly more strongly (negatively) associated with competitive-world than dangerous-world beliefs (.19 [.01, .37], *p* = .042).

#### Variation across different facets of humanism and normativism

Correlations between the different facets of humanism and normativism and ideological variables are presented in Table 2. The results show clearly that the associations between humanism and normativism and ideological orientations were not limited solely to the political values facets. Humanist and normativist views of human nature and interpersonal attitudes, for example, were at least as strongly linked as political values to preference for equality, SDO, and competitive-world beliefs.

**Table 2:** Correlations across different facets of humanism and normativism in Study 1 (U.S.).

	Humanism					Normativism				
	Human nature	Inter-personal	Affect	Epistem-ology	Political values	Human nature	Inter-personal	Affect	Epistem-ology	Political values
General ideological self-placement	-.16***	-.18***	-.16**	-.20***	-.36***	.26***	.30***	.22***	.23***	.40***
Social ideology self-placement	.12	-.08	-.17**	-.24***	-.33***	.17**	.14*	.13	.21**	.26***
Economic ideology self-placement	-.08	-.13	-.12	-.16*	-.30***	.21***	.25***	.17*	.15*	.33***
Resistance to change	-.05	-.17*	-.14	-.05	-.23***	.18*	.23***	.24**	.25***	.37***
Preference for equality	.36***	.41***	.16*	.15	.35***	-.30***	-.46***	-.23**	-.17*	-.24**
RWA	-.17***	-.26***	-.25***	-.24***	-.42***	.31***	.30***	.28***	.27***	.46***
SDO	-.41***	-.53***	-.28***	-.31***	-.53***	.38***	.45***	.27***	.20***	.34***
Dangerous-world beliefs	-.37***	-.25***	-.24***	-.22***	-.36***	.45***	.37***	.35***	.35***	.47***
Competitive-world beliefs	-.48***	-.54***	-.31***	-.28***	-.42***	.53***	.56***	.35***	.35***	.34***
General SJ	.03	-.18*	-.08	-.02	-.08	.04	.18**	.14	.25**	.33***
Economic SJ	-.23***	-.36***	-.14**	-.15**	-.36***	.38***	.44***	.26***	.34***	.44***
Need for closure	-.18*	-.05	-.03	.02	-.09	.19**	.18*	.09	.28***	.31***
Death anxiety	.02	-.03	.05	.00	.00	.10	.09	.05	.13*	.12*
Insecure attachment	-.02	.01	.02	.07	-.02	.18***	.18***	.20***	.13*	.07
Fear of terrorism	-.07	-.05	.04	-.06	-.08	.10	.14**	.10	.18***	.33***

* *p* < .05.

** *p* < .01.

*** *p* < .001.

### Discussion

The results of this study suggest that humanism and normativism are indeed associated with leftist and rightist ideological orientations respectively. As hypothesized, they were correlated with ideological self-placement (on social and economic issues), political attitudes, beliefs about the social world, and underlying motivations. An exploratory analysis revealed that the results generalized fairly well across the different facets of humanism and normativism. The one notable exception was that humanism was unrelated to epistemic and existential motives, whereas normativism was significantly associated with need for closure, insecure attachment, and fear of terrorism, but not death anxiety in general.

In terms of the model of political ideology as motivated social cognition, we found that humanism was directly associated only with preference for equality, whereas normativism was directly associated with resistance to change and acceptance of inequality. In terms of the dual process model, on the other hand, humanism was directly associated with (low) RWA, SDO, and dangerous- and competitive-world beliefs, whereas normativism was directly associated with RWA and dangerous- and competitive-world beliefs.

One limitation of this study is that participants were very homogeneous in terms of age, education, socio-economic background, and political orientation. The fact that more than two-thirds of the participants described themselves as left-of-center makes it difficult to generalize the results to those anchoring the right-wing pole. Another limitation of the study is that we tested our first hypothesis (H1) solely in terms of ideological self-placement. Consequently, it could be argued that humanism and normativism may be associated with leftist and rightist social identities but not actual ideological preferences. We addressed both of these limitations in Study 2.

## Study 2

In this study, we sought to replicate the findings of Study 1, investigating patterns of associations between humanist and normativist worldviews and variables specified by the two psychological models of political ideology with a more diverse online sample. We also included a measure of issue-based preferences.

### Method

#### Participants

Participants were 346 U.S. adults (mean age = 38.1, *SD* = 13.13; 60.2% women; 10.8% master’s degree; 37.3% bachelor’s degree; 15.7% lower college degree; 18.7% college without degree; 17.5% high school or lower) who completed the study online. They were recruited and compensated ($0.40) for their participation through Amazon’s Mechanical Turk website [86] using Qualtrics software. Ideological self-placement was left-of-center for 49.7% and right-of-center for 26.0% of the participants. The sample size gave us 80% power (two-tailed) to detect associations of |*r*| = .15. Missing data did not affect this power estimate (*n* ≥ 341 for all variables).

#### Measures

We measured humanism (*M* = 5.17, *SD* = .75, *α* = .82) and normativism (*M* = 4.12, *SD* = .74, *α* = .75) with 15-item short-versions of the scales used in Study 1 [25, 68]. We measured epistemic motivation using a 15-item shortened version [87] of the need for cognitive closure scale (*M* = 4.48, *SD* = 1.02, *α* = .89). We measured all other variables with the same items and scales administered in Study 1 (Resistance to change: *M* = 3.96, *SD* = 1.07, *α* = .86; Preference for equality: *M* = 5.09, *SD* = 1.04, *α* = .82; General system justification: *M* = 3.81, *SD* = 1.09, *α* = .79; Economic system justification: *M* = 3.45, *SD* = 1.26, *α* = .86; RWA: *M* = 3.48, *SD* = 1.18, *α* = .90; SDO: *M* = 2.76, *SD* = 1.24, *α* = .86; Dangerous-world beliefs: *M* = 3.95, *SD* = 1.29, *α* = .90; Competitive-world beliefs: *M* = 2.77, *SD* = .97, *α* = .83; Death anxiety: *M* = 3.98, *SD* = 1.50, *α* = .81; Insecure attachment: *M* = 3.01, *SD* = 1.06, *α* = .84). Participants responded on a Likert scale ranging from 1 (*Strongly disagree*) to 7 (*Strongly agree*) to all of the scales mentioned above.

As in Study 1, participants responded on 1–9 Likert scales to single-item measures of liberal-conservative self-placement in general (*M* = 4.28, *SD* = 2.24), in terms of social issues (*M* = 3.92, *SD* = 2.39), and in terms of economic issues (*M* = 4.78, *SD* = 2.27).

We measured ideological preferences with Everett’s [88] conservatism scale, which asks people to judge how positive or negative they feel about 12 issues (e.g., abortion, welfare benefits, and patriotism) on feeling thermometers ranging from 0 to 100. We added four issues (labor unions, gay marriage, affirmative action, and the death penalty) from Inbar, Pizarro, and Bloom’s [89] scale, bringing the total number of items to 16 (*M* = 55.4, *SD* = 15.5, *α* = .87). We placed the issue-based measure of conservative preferences before the existential and epistemic motivations. Apart from this, the order of the scales was the same as in Study 1.

#### Statistical procedure

We used the same statistical procedure as in Study 1. But we replaced the single-item ideological self-placement item with a latent factor representing left-right ideology that was based on both issue preferences and ideological self-placement (see supplementary documentation for details) in our structural equation models. The measurement models exhibited high omega total reliabilities (the model of ideology as social cognition: *ω*_*t*_ = .92; the dual process model: *ω*_*t*_ = .95) and the factor loadings were adequate (Humanism: *λ* ≥ .61; Normativism: *λ* ≥ .35; Resistance to change: *λ* ≥ .84; Preference for equality: *λ* ≥ .66; System justification: *λ* ≥ .76; RWA: *λ* ≥ .85; SDO: *λ* ≥ .85; Dangerous-world belief: *λ* ≥ .84; Competitive-world belief: *λ* ≥ .80; Left-right ideology: *λ ≥* .76).

Model fit was somewhat lower in this study than in the first study both for the model of ideology as social cognition, *χ*^*2*^(117) = 435.6, *p* < .001, CFI = .902, RMSEA = .089[.080, .098], and for the dual process model, *χ*^*2*^(169) = 689.3, *p* < .001, CFI = .896, RMSEA = .094[.087, .102]. Follow-up analyses revealed that the misfit was mainly due to imperfections in the measurement model stemming from item-factor cross-loadings. But the fit of the models we tested and the degree of overlap between constructs was comparable to that of a model comprised solely of the four constructs (RWA, SDO, and dangerous- and competitive-world beliefs) that make up the well-established dual process model (see supplementary documentation). We therefore did not consider it to be realistic to expect the scales to be perfectly separate in every case. But we were able to increase fit of the model building on the dual process account of ideology by adding direct paths from competitive- and dangerous-world beliefs to left-right ideology, Δ*χ*^*2*^(2) = 7.40, *p* = .025, *χ*^*2*^(167) = 681.9, *p* < .001, CFI = .897, RMSEA = .095[.087, .102]. Both of these variables predicted a leftist orientation when all other constructs were adjusted for (see Fig 4).

### Results

Correlations between the variables that were included in this study are summarized in Table 3. Humanism was robustly associated with a more leftist (or liberal) self-placement, consistent with (H1a). However, the association between humanism and issue-based preferences did not reach significance when we adjusted the significance threshold for the number of tests (*p* = .020; adjusted *p*-threshold = .007). Because the measure of issue-based preferences focused primarily on conservative issues (e.g., religion, traditional values, and fiscal responsibility), we conducted a *post hoc* analysis of the correlation between humanism and preferences for those four issues that were most aligned with a liberal worldview (welfare benefits, labor unions, gay marriage, affirmative action; *α* = .65), and this correlation was strong (*r* = .36, *p* < .001).

**Table 3:** Correlations involving humanism, normativism, ideology, and motivation in Study 2 (U.S.).

	1	2	3	4	5	6	7	8	9	10	11	12	13	14	15	16	17
1. Humanism																	
2. Normativism	-.11*																
3. General ideological self-placement	-.23***	.27***															
4. Social ideology self-placement	-.21***	.25***	.83***														
5. Economic ideology self-placement	-.27***	.23***	.83***	.64***													
6. Conservative preferences	-.13*	.35***	.69***	.64***	.64***												
7. Resistance to change	-.10	.42***	.66***	.69***	.58***	.76***											
8. Preference for equality	.61***	-.23***	-.39***	-.30***	-.46***	-.41***	-.33***										
9. RWA	-.15**	.38***	.61***	.69***	.49***	.69***	.82***	-.30***									
10. SDO	-.52***	.32***	.41***	.37***	.44***	.44***	.43***	-.71***	.44***								
11. Dangerous-world beliefs	-.10	.41***	.37***	.45***	.28***	.45***	.61***	-.12*	.66***	.26***							
12. Competitive-world beliefs	-.48***	.43***	.15**	.16***	.19***	.19***	.23***	-.53***	.26***	.64***	.21***						
13. General SJ	-.07	.11*	.26***	.20***	.28***	.34***	.26***	-.29***	.19***	.26***	-.23***	.21***					
14. Economic SJ	-.29***	.38***	.51***	.43***	.54***	.62***	.54***	-.66***	.52***	.64***	.21***	.46***	.64***				
15. Need for closure	.18***	.23***	.14*	.14*	.11*	.17**	.25***	.12*	.24***	-.03	.22***	-.04	.09	.11*			
16. Death anxiety	.04	.15**	.06	.02	.05	.07	.16**	-.05	.02	.04	.02	.14**	.20***	.15**	.37***		
17. Insecure attachment	-.14**	.17**	-.04	.02	-.06	-.07	.06	-.19***	.09	.27***	.09	.41***	-.02	.08	.17**	.16**	
18. Fear of terrorism	-.02	.30***	.38***	.38***	.36***	.51***	.54***	-.18**	.53***	.25***	.51***	.17**	.25***	.38***	.37***	.28***	.05

* *p* ≤ .05.

** *p* < .01.

*** *p* < .001.

Normativism was associated with more rightist (or conservative) self-placements and issue-based preferences, consistent with (H1b).

#### The model of ideology as motivated social cognition

With respect to (H2a), humanism was strongly associated with preference for equality and (low) economic system justification but was not significantly associated with openness to change, general system justification, or epistemic and existential motives. The negative correlation between humanism and insecure attachment did not reach significance when we adjusted the significance threshold (*p* = .008; adjusted *p*-threshold = .006), and humanism was *positively* associated with need for closure, contrary to our expectations.

As in Study 1, support for (H2b) was much clearer. Normativism was significantly associated with resistance to change, acceptance of inequality, economic system justification, and all measures of existential and epistemic motives. Correlations with death anxiety (*p* = .004; adjusted *p*-threshold = .006) and insecure attachment (*p* = .002; adjusted *p*-threshold = .005) were marginally significant. However, the association between normativism and general system justification did not reach significance when we adjusted the significance threshold to control the error rate (*p* = .040; adjusted *p*-threshold = .008).

As shown in Fig 3, the direct association between humanism and openness to change did reach significance when we tested the entire model of ideology as motivated social cognition through structural equation modelling. But this association was much weaker than the association between humanism and preference for equality (.67[.46, .89], *p* < .001). Normativism, on the other hand, was directly associated with resistance to change and system justification but not acceptance of inequality when all variables were taken into account.

#### The dual-process model

Humanism was negatively associated with RWA, SDO, and competitive-world beliefs, consistent with (H3a), but the association between humanism and dangerous-world beliefs was not significant (*p* = .075; adjusted *p*-threshold = .013). The negative association between humanism and RWA was marginally significant (*p* = .004; adjusted *p*-threshold = .005). Normativism was consistently associated with RWA, SDO, and dangerous- and competitive-world beliefs, providing unambiguous support for (H3b) once again.

Humanism was negatively associated with SDO and competitive-world beliefs but not RWA or dangerous-world beliefs when we took the entire dual-process model into account through structural equation modelling, as illustrated in Fig 4. Normativism, on the other hand, was strongly associated with dangerous- and competitive-world beliefs (.47[-.31, 1.32], *p* = .38) and RWA and SDO (.39[-.37, .93], *p* = .32).

### Discussion

The results of this study provide further evidence that normativism is consistently associated with a wide range of aspects of a rightist (or conservative) ideological orientation. With respect to the model of ideology as motivated social cognition, the results suggest that normativism is particularly strongly and directly associated with resistance to change but correlated also with acceptance of inequality, economic system justification, and epistemic and existential motives. With respect to the dual process model, this study gave no indication that normativism would be more closely associated with RWA and dangerous-world beliefs than with SDO and competitive-world beliefs.

The results of this study provide further evidence also that humanism is primarily associated with preference for equality rather than openness to change, and economic rather than general system justification, and that it is unrelated to epistemic and existential needs. In addition, the fact that humanism was more directly associated with (low) SDO than RWA and with (low) competitive- than dangerous-world beliefs in this study also suggests that humanism is associated with political orientation mainly because of its opposition to hierarchy and competitiveness rather than a desire to challenge traditional authorities or norms *per se*. Humanism was not significantly correlated with the measure of issue-based preferences we used in this study, but a *post hoc* analysis suggested that this may have been due to the fact that the measure we used focused primarily on traditional conservative issues. Humanism was strongly correlated with preferences regarding those issues that should resonate the most with a liberal worldview. In addition, it should be noted that the measures of issue-preferences and ideological self-placements correlated so strongly that we were able to model them as indicators of a common latent factor. These results suggest that humanism and normativism are associated with actual ideological preferences—but perhaps not always the same ones—rather than just social identities.

Taken together, the results of the first two studies provide evidence that normativism and to some extent humanism do permeate ideological orientations in a manner consistent with polarity theory. The results of Study 1 largely held up with a more diverse sample of participants recruited in Study 2. Nevertheless, the first two studies were limited to the U.S. cultural and political context. In Study 3 and 4, we tested our hypotheses in Sweden.

## Study 3

In this study, we investigated the associations between humanist and normativist worldviews and the dual-process model of ideology in a sample of Swedish adults, and we also included brief measures of epistemic and existential motives.

### Method

**Participants.** Participants were 360 Swedish adults (mean age = 30.5, *SD* = 13.31, 41.3% women) with an average of 2.83 (*SD* = 2.72) years of college or university education. They were recruited in universities, trains, and other public spaces mainly in southern Sweden, filled out written questionnaires, and were compensated with a lottery ticket or chocolate bar. Ideological self-placement was left-of-center for 64.5% and right-of-center for 35.5% of the participants. Scales measuring epistemic and existential needs were completed by 182 of the participants (mean age = 31.3, *SD* = 13.45, 54.1% women). A posteriori power analysis showed that we had at least 80% power (two-tailed) to detect correlations of |*r*| = .15 with the full sample (*n* ≥ 338 for all variables) and |*r*| = .20 to .21 (*n* ≥ 178) with the subsample.

#### Measures

We measured humanism (*M* = 3.81, *SD* = .54, *α* = .83) and normativism (*M* = 2.86, *SD* = .47, *α* = .74) with the 15-item short-scales used in Study 2. We measured RWA (*M* = 2.38, *SD* = .49, *α* = .81), SDO (*M* = 1.81, *SD* = .61, *α* = .77), dangerous-world beliefs (*M* = 2.52, *SD* = .62, *α* = .78), and competitive-world beliefs (*M* = 1.94, *SD* = .57, *α* = .79) with the same scales used in Study 1–2. We measured need for closure (*M* = 3.06, *SD* = .55, *α* = .78) with the short-scale used in Study 2. We measured death anxiety (*M* = 2.25, *SD* = .91, *α* = .87) with the four items used in Study 1, complemented with four items focused on behavioral manifestations of the fear of death, including “The sight of a corpse deeply shocks me” [90]. Participants responded on Likert scales ranging from 1 (*Strongly disagree*) to 5 (*Strongly agree*). They reported ideological self-placement (“In political matters, people sometimes talk about ‘the left’ and ‘the right.’ How would you place your views on this scale, generally speaking?”; *M* = 4.54, *SD* = 2.24) on a scale ranging from 1 (*Left*) to 10 (*Right*). They completed the humanism and normativism scales first, followed by SDO, RWA, dangerous-world beliefs, and competitive-world beliefs. All of the measures were translated into Swedish.

#### Statistical procedure

We followed the same statistical procedure as in the previous studies. We addressed the dual-process model of ideology through structural equation modelling, exactly as in Study 1 (Fig 2). The measurement model was adequate in terms of reliability (*ω*_*t*_ = .91) and factor loadings (Humanism: *λ* ≥ .67; Normativism: *λ* ≥ .36; RWA: *λ* ≥ .60; SDO: *λ* ≥ .74; Dangerous-world belief: *λ* ≥ .71; Competitive-world belief: *λ* ≥ .75). But the complete model exhibited some misfit mainly due to item-factor cross-loadings, as in Study 2, *χ*^*2*^(133) = 464.8 *p* < .001, CFI = .880, RMSEA = .083[.075, .092]. Once again, we were able to increase the fit by adding direct paths from dangerous- and competitive-world beliefs to left-right placement (see Fig 5), Δ*χ*^*2*^(2) = 14.9 *p* < .001, *χ*^*2*^(131) = 449.9 *p* < .001, CFI = .885, RMSEA = .082[.074, .091].

### Results

Correlations among study variables are shown in Table 4. The association between normativism and a rightist ideological orientation was, yet again, more consistent across different variables than the association between humanism and leftist orientation was. Humanism was in fact uncorrelated with ideological self-placement in this study, contrary to (H1a). Humanism was uncorrelated with death anxiety and need for closure when we adjusted the significance threshold (*p* = .018; adjusted *p*-threshold = .007), contrary to (H2a). Humanism was negatively correlated with SDO, consistent with (H3a), but it was not correlated with RWA or dangerous-world beliefs, and it was not significantly correlated with competitive-world beliefs when we adjusted the significance threshold (*p* = .028; adjusted *p*-threshold = .008). These results provide mixed support for (H3a).

**Table 4:** Correlations involving humanism, normativism, ideology, and motivation in Study 3 (Sweden).

	1	2	3	4	5	6	7	8
1. Humanism								
2. Normativism	.01							
3. Ideological self-placement	.05	.26***						
4. RWA	.03	.41***	.37***					
5. SDO	-.28***	.35***	.43***	.41***				
6. Dangerous-world beliefs	-.03	.38***	.16**	.60***	.34***			
7. Competitive-world beliefs	-.12*	.45***	.39***	.39***	.57***	.39***		
8. Need for closure	.18*	.36***	.18*	.40***	.25**	.41***	.29***	
9. Death anxiety	-.01	.03	-.04	.04	.05	.10	.17*	.23**

* *p* ≤ .05.

** *p* < .01.

*** *p* < .001.

Normativism, on the other hand, was correlated with rightist self-placement, RWA, SDO, dangerous- and competitive world beliefs, and need for closure but not death anxiety. These results again provide clear support for (H1b) and (H3b) but more mixed support for (H2b).

Structural equation modelling also failed to identify an association between humanism and RWA and dangerous- and competitive-world beliefs, as shown in Fig 5. Humanism was directly associated with *rightist* self-placement after adjusting for all variables specified by the dual-process model. Normativism, on the hand, was strongly, directly, and equivalently associated with dangerous- and competitive-world beliefs (-.10[-.33, .13], *p* = .37) and with RWA and SDO (.13[-.17, .56], *p* = .44).

### Discussion

The results of this study corroborate the observation from the first two studies that normativism was consistently associated with a wide range of aspects of ideological orientations even in a very different cultural and political context. The sole exception was that normativism failed to correlate with death anxiety, which is largely consistent with previous results; the correlation was non-significant in Study 1 and marginally significant in Study 2. The results of the first three studies suggest that normativism is no more closely associated with RWA and dangerous-world beliefs than with SDO and competitive-world beliefs.

The results of Study 3 were also very similar to those of the first two studies in that the association between humanism and leftist ideological orientation was limited to specific aspects. Humanism was unrelated to epistemic and existential motives, consistent with the results of the first two studies. Humanism was also unrelated to ideological self-placement, RWA, and dangerous- and competitive-world beliefs in Study 3. Taken in conjunction, the results of the first three studies suggest that humanism is most robustly and directly associated with aversion to hierarchy. The association between humanism and other types of ideological preferences, beliefs, and self-placements may be more contingent on contextual factors. For example, the fact that humanistic tendencies are prevalent on both the left and the right in Sweden might explain the lack of association between humanism and leftist self-placement. It is also quite possible that the rightists we recruited for this study were among the most humanistically oriented group of rightists in Sweden (e.g., social liberals).

## Study 4

In this study, we investigated the associations between humanist and normativist worldviews and the model of ideology as motivated social cognition in a sample of Swedish university students. We also took the HEXACO model of personality traits into consideration.

### Method

#### Participants

Participants were 332 Swedish Lund University students (mean age = 31.3, *SD* = 13.45, 54.1% women; behavioral sciences, *n* = 210; law, *n* = 103; engineering and economics, *n* = 19). They were recruited in public university spaces, filled out written questionnaires, and were compensated with a brownie. Ideological self-placement was left-of-center for 57.3% and right-of-center for 31.3% of the participants. The full sample gave us 80% power (two-tailed) to detect correlations of |*r*| = .15 (*n* ≥ 325 with the exception of conscientiousness, which was dropped after the first phase of data collection, *n* = 99).

#### Measures

We measured humanism (*M* = 5.47, *SD* = .61, *α* = .75) and normativism (*M* = 3.53, *SD* = .75, *α* = .79) with the 15-item short scales used in Studies 2 and 3. We measured resistance to change (10 items; *M* = 3.27, *SD* = .79, *α* = .74), and preference for equality (7 items; *M* = 5.56, *SD* = .91, *α* = .80) using scales from Study 1 with minor changes made to adapt them to the Swedish setting. We measured general system justification (*M* = 3.99, *SD* = 1.12, *α* = .84) with the scale used in Study 1 and economic system justification (*M* = 3.27, *SD* = 1.45, *α* = .84) with five of the items used in Study 1. We measured honesty-humility (*M* = 4.31, *SD* = .79, *α* = .67), emotionality (*M* = 4.57, *SD* = .92, *α* = .75), openness (*M* = 5.14, *SD* = .88, *α* = .72), and conscientiousness (*M* = 4.86, *SD* = .95, *α* = .80) with a short-version [52] of the HEXACO scale [91] that includes 10 items per trait, such as “I would be quite bored by a visit to an art gallery” (openness, reverse-scored) and “I feel like crying when I see other people crying” (emotionality). We include conscientiousness in correlational analyses but not structural equation modeling because of missing values.

Participants responded to the items measuring all constructs on Likert scales ranging from 1 (*Strongly disagree*) to 7 (*Strongly agree*). They reported their ideological self-placement (“Where would you place yourself on the following scale of political orientation?”; *M* = 4.34, *SD* = 1.81) on a Likert scale ranging from 1 (*Extremely left-wing*) to 9 (*Extremely right-wing*). In terms of questionnaire order, participants completed the humanism and normativism scales first with items presented in randomized order, followed by all political attitude items in randomized order, the moral foundations questionnaire, and all personality and motivational dispositions with items presented in randomized order. All of the measures were translated into Swedish.

#### Statistical procedure

**Fig 6:**
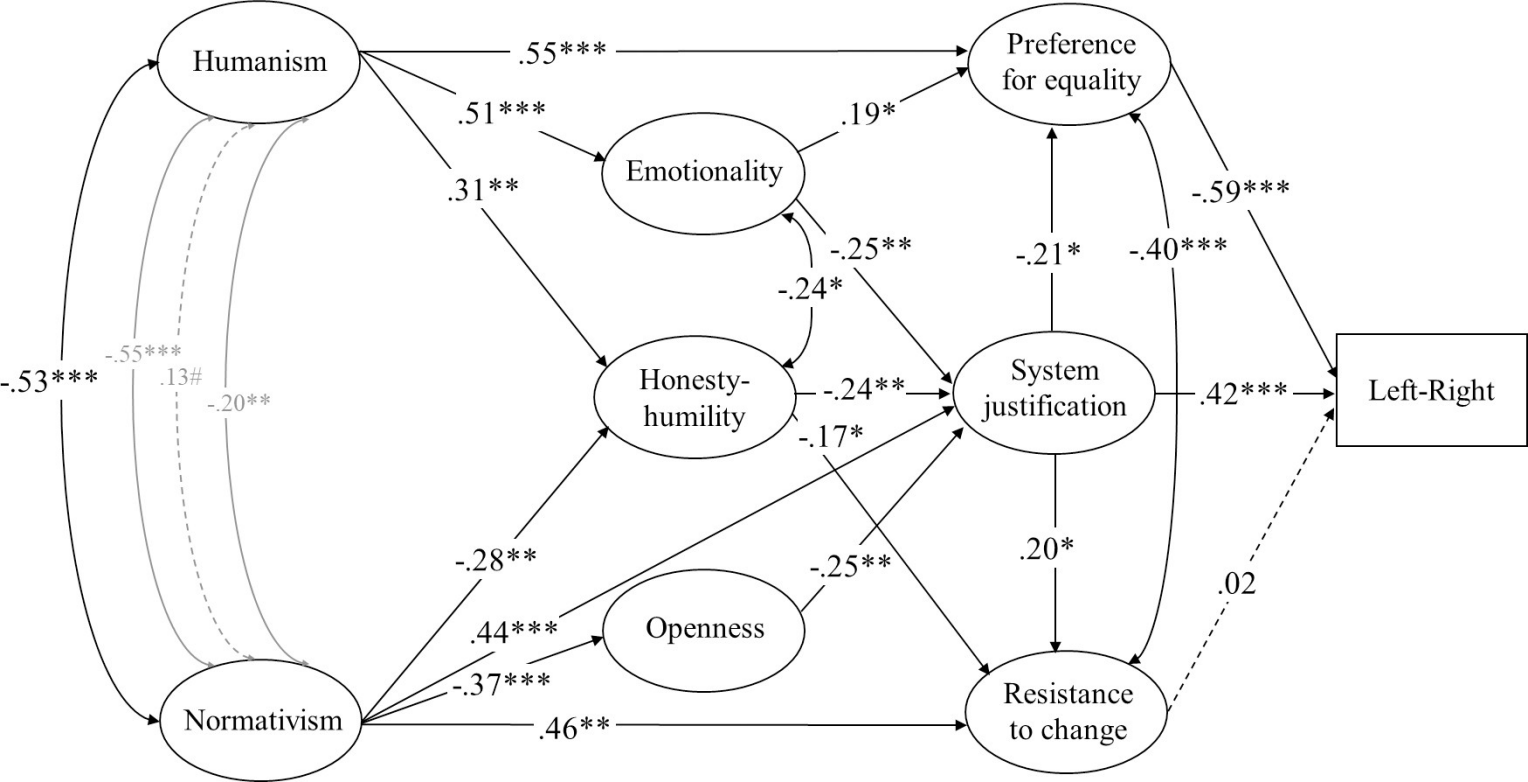
Structural equation model (standardized solution) incorporating the HEXACO traits and the model of ideology as social cognition in Study 4. # *p* < .10, * *p* < .05, ** *p* < .01, *** *p* < .001. Disturbances and factor loadings are not shown. Non-significant paths are omitted with the exception of the path from resistance to change to ideological self-placement.

We followed the same statistical procedure as in the previous studies. We used structural equation modelling to test the model of ideology as motivated social cognition that was introduced in Study 1. We then explored an even more comprehensive model, which retained the core structure but added emotionality, honesty-humility, and openness as potential mediators of the paths from humanism and normativism to system justification, resistance to change, preference for equality, and ideological self-placement (Fig 6). The model included paths from humanism and normativism to all other constructs and paths from these traits to system justification, resistance to change, preference for equality, and ideological self-placement. The measurement models exhibited adequate reliabilities (*ω*_*t*_ = .90 for both cases) and factor loadings (Humanism: *λ* ≥ .53; Normativism: *λ* ≥ .55; Resistance to change: *λ* ≥ .50; Preference for equality: *λ* ≥ .72; System justification: *λ* ≥ .74; Openness: *λ* ≥ .48; Honesty: *λ* ≥ .23; Emotionality: *λ* ≥ .49). The fit of the full structural equation model was acceptable both without traits, *χ*^*2*^(87) = 257.9, *p* < .001, CFI = .920, RMSEA = .077[.068, .088], and with traits, *χ*^*2*^(312) = 702.7 *p* < .001, CFI = .868, RMSEA = .062[.055, .068]. Although CFI was low in the latter case, it is not meaningful to evaluate CFI in terms of standard fit criteria when the RMSEA of the independence model is below .158 [63], and this was the case here (it was .148).

### Results

Table 5 shows correlations bearing on our hypotheses in this study. Humanism was strongly associated with leftist ideological self-placement, consistent with (H1a), and normativism was strongly associated with rightist self-placement, consistent with (H1b).

**Table 5:** Correlations involving humanism, normativism, ideology, and personality traits in Study 4 (Sweden).

	1	2	3	4	5	6	7	8	9	10
1. Humanism										
2. Normativism	-.43***									
3. Ideological self-placement	-.34***	.47***								
4. Resistance to change	-.27***	.49***	.55***							
5. Preference for equality	.57***	-.41***	-.61***	-.50***						
6. General SJ	-.25***	.40***	.67***	.50***	-.51***					
7. Economic SJ	-.35***	.54***	.76***	.57***	-.62***	.79***				
8. Openness	.16**	-.28***	-.31***	-.29***	.28***	-.28***	-.37***			
9. Honesty-humility	.28***	-.21***	-.21***	-.26***	.30***	-.18**	-.23***	.13*		
10. Emotionality	.32***	-.13*	-.14*	-.06	.34***	-.22***	-.18***	-.06	.12*	
11. Conscientiousness	.05	.07	.23*	.17	-.09	.08	.16	-.05	.14	.10

* *p* ≤ .05.

** *p* < .01.

*** *p* < .001.

#### The model of ideology as motivated social cognition

Humanism was associated with openness to change, preference for equality, and (low) general and economic system justification, whereas normativism was associated with resistance to change, acceptance of inequality, and general and economic system justification. These results provide unequivocal support for the aspects of (H2a) and (H2b) we tested in this study.

**Fig 7:**
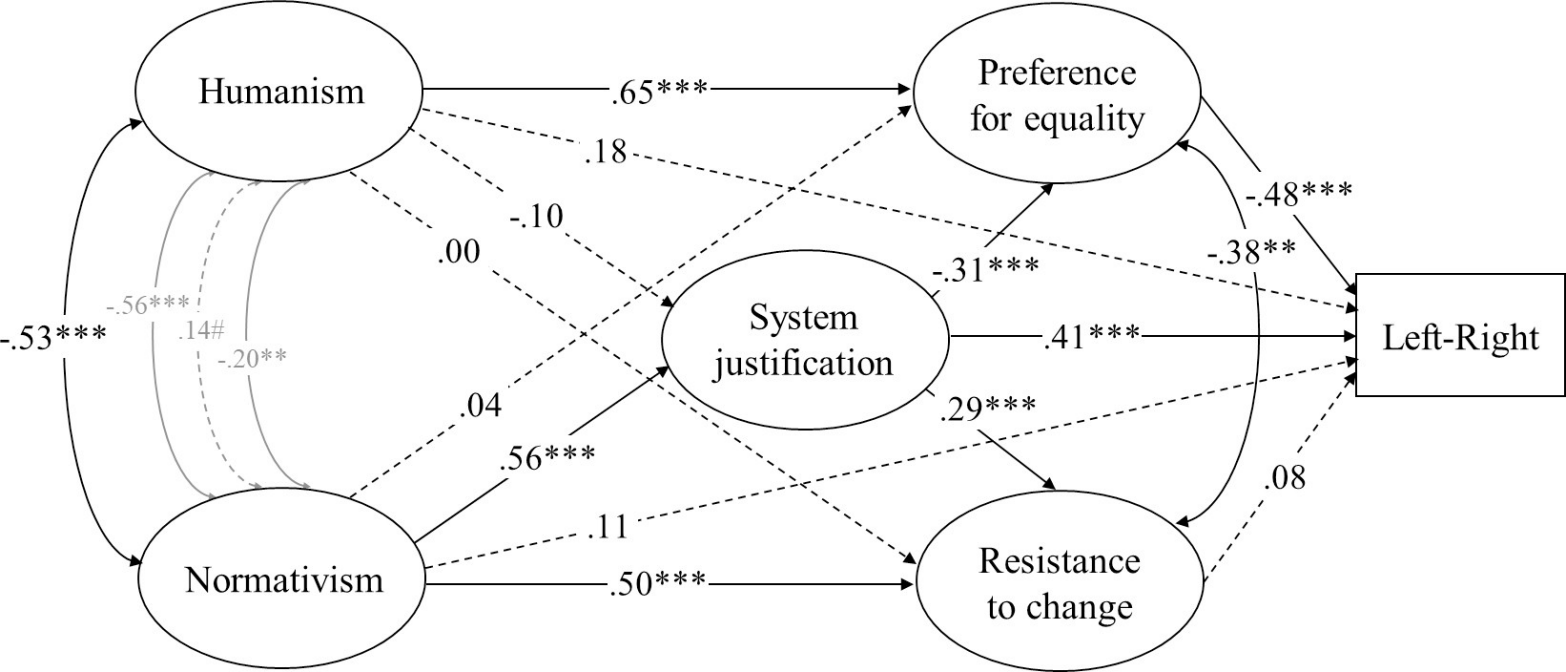
Structural equation model (standardized solution) of associations between humanist and normativist worldviews and the model of ideology as motivated social cognition in Study 4. # *p* < .10, * *p* < .05, ** *p* < .01, *** *p* < .001 (dotted lines represent non-significant estimates). Disturbances and factor loadings are not shown.

Structural equation modelling suggested that normativism was directly associated with resistance to change and system justification but not acceptance of inequality when the entire model of ideology as social cognition was taken into consideration, as shown in Fig 7. Humanism, on the other hand, was directly associated only with preference for equality when all variables were taken into account.

#### The HEXACO traits

In this study, we were able to address (H4) as well. As shown in Table 4, humanism was indeed significantly and positively associated with openness, emotionality, and honesty-humility. Thus, (H4a) was clearly supported. In addition, normativism was negatively associated with openness and honesty-humility, consistent with (H4b), and the correlation between normativism and emotionality was negative as well but only marginally significant (*p* = .020; adjusted *p*-threshold = .017).

Next we tested the entire model of ideology as social cognition and HEXACO traits through structural equation modeling. We observed that humanism was still directly associated with preference for equality, emotionality, and honesty-humility and normativism was still directly associated with resistance to change, system justification, and (low) openness and honesty-humility.

### Discussion

The results of this study provide further support for the notion that normativism is robustly associated with rightist ideological preferences and motivations. When we investigated the entire model of ideology as motivated social cognition through structural equation modelling we observed that normativism was directly associated with resistance to change and system justification but not acceptance of inequality. Thus, across studies, we found that normativism was more closely linked to resistance to change than acceptance of inequality when these specific ideological components were isolated. Humanism, on the other hand, was most closely linked to preferences for equality. It should be noted that humanism was associated with leftist self-placement, openness to change, and low system justification in Study 4. This indicates that the lack of a significant correlation between humanism and ideological self-placement (and other constructs) in Study 3 cannot be explained solely in terms of the Swedish cultural and political context.

In Study 4 we also observed that humanism and normativism were associated with personality traits that have well-known affinities with political preferences. In terms of the HEXACO model, humanism was positively correlated with emotionality, openness, and honesty-humility, whereas normativism was correlated negatively with these three traits. Structural equation modelling suggested that emotionality was more directly associated with humanism, whereas openness was more directly associated with (low) normativism. Honesty-humility was directly associated (in opposite directions) with humanism and normativism. Taken together, these findings provide additional support for Tomkins’ approach, which suggests that a profound resonance existing between opposing left-right ideological worldviews and the structure of personality, which is comprised of affective, cognitive, and motivational substrates.

## General discussion

The results of four studies conducted in the U.S. and Sweden were highly supportive of Tomkins’ [1, 19] contention that normativism is associated with a rightist political orientation, whereas humanism is associated with a leftist political orientation. Normativism was consistently associated with all of the social and political attitudes, beliefs, motivations, and personality traits that are characteristic of conservatives, with the exceptions of death anxiety and conscientiousness (in the Swedish samples). Analyses bearing on the motivated social cognition model introduced by Jost and colleagues [15] revealed that normativism was consistently associated with resistance to change, acceptance of inequality, and system justification in both U.S. and Swedish samples. It was directly associated with resistance to change in all three of the studies in which the association was tested and with system justification in two of the three studies, and with acceptance of inequality in one of three studies when all constructs were adjusted for through structural equation modelling. These findings are supportive of both polarity theory and the model of ideology as motivated social cognition; they suggest that normativism may be especially closely linked to resistance to change.

Analyses bearing on the dual process model introduced by Duckitt [22] indicated that normativism was consistently associated with right-wing authoritarianism (RWA) and social dominance orientation (SDO) as well as dangerous- and competitive-world beliefs. All of this is highly consistent not only with polarity theory but also with the dual process model. It is worth noting that normativism was more directly and strongly linked to RWA and dangerous-world beliefs than to SDO and competitive-world beliefs in only one of three studies. In general, normativism was independently associated with each of the variables specified by the dual process model.

Finally, we considered an integrative model that incorporated personality traits as well as the foregoing variables. We observed that normativism was negatively associated with openness and honesty-humility, and these associations held up even after we adjusted for the variables specified by the model of ideology as motivated social cognition. Normativism was negatively associated with emotionality to a marginal degree.

Results pertaining to humanism were somewhat more complex. Humanism was associated with liberal or leftist self-placement in three of four studies. In terms of the motivated social cognition model, humanism was consistently associated with high preference for equality and low economic system justification, but not with resistance to change or general system justification. The association between humanism and preference for equality was very robust even when other variables were adjusted for. These findings are generally—but not unequivocally—supportive of a hybrid model that combines polarity theory and the model of ideology as motivated social cognition. At the same time, humanism was largely unrelated to existential needs, and it was associated with high rather than low need for cognitive closure in one of three studies. This last finding was surprising and suggests the need for additional research.

In terms of the dual process model, the negative association between humanism and SDO held up in the three studies in which it was tested, and the association between humanism and cooperative-world beliefs held up in two of three studies with and without adjustments for other variables. Humanism was negatively associated with RWA in two studies (and in one when adjusting for other variables) and with dangerous-world beliefs in one study.

Analyses incorporating the HEXACO traits model as well as the model of ideology as motivated social cognition revealed that humanism was directly and robustly associated with emotionality and honesty-humility (and it was correlated with openness as well). These results suggest that polarity theory may be useful for understanding how beliefs, motives, values, and personality traits come to be structured along a left-right ideological dimension. Humanism and normativism, which were significantly and negatively correlated in three of the four studies, may indeed lend organization and structure to the individual’s personality and his or her worldview, as Tomkins [1, 19, 23] proposed.

On the basis of the four studies reported here, we would conclude that normativism represents a highly general (or expansive) worldview on the right, whereas humanism was linked more narrowly to certain elements of a left-leaning worldview. Humanism appears to be associated with leftist ideology because it involves a desire to promote equality and social justice (and to end human suffering); it does not seem to entail enthusiasm for social change *per se*. This is consistent with Tomkins’ [1] observation that it is only “during those historical periods when social and political authority is seen as violating the rights and dignity of man [that the left-wing ideologist] is apt to set himself in violent opposition to tradition” (p. 408).

One might infer from all of this that normativism is the more fundamental construct of the two. This is possible, but it is also conceivable that we have simply done a better job of measuring normativism (compared to humanism), perhaps because it is an easier construct to measure. In any case, both the motivated social cognition [15] and dual process [22] models, which we drew upon heavily in the current research program, focus predominantly (but not exclusively) on the motivational underpinnings of conservative or right-wing ideology. Humanistic motivation, as conceptualized by Tomkins [1, 19], may be especially useful for explaining why some individuals are attracted to liberal or left-wing ideology.

It is possible that the sets of traits, motivations, and beliefs that foster an individual’s attraction to left (vs. right) or liberal (vs. conservative) ideas comprise somewhat distinct psychological systems that contribute to ideological divergence (and polarization). Previous research suggests that humanism and normativism are factorially distinct [25], and that they are associated with different goals, values, and philosophical assumptions about the nature of mind and reality, among other things (see [31]).

The philosophical contrast between humanism and normativism is a critical one because it helps to explain how distinct psychological systems could produce ideologies that are, nevertheless, polarized along a single left-right continuum. Warmth, openness, creativity, curiosity, and support for human rights are not necessarily semantic opposites of rigor, discipline, rule-following, and support for social order. In addition, as Tomkins [1] pointed out, there are “middle of the road” ideologies that creatively synthesize aspects of humanism and normativism. At the same time, there is clearly an underlying tension—and therefore the *potential* for conflict—between value priorities and philosophical assumptions associated with humanistic and normative approaches to life. All of this could help to explain why the same (or similar) polarities tend to recur, over and over again, in politics, philosophy, science, art, and religion [1, 26, 27, 31, 42, 92, 93].

Despite a great many historical, cultural, political, and linguistic differences between the U.S. and Sweden, we obtained very similar results overall when testing our models in the two contexts. This speaks in favor of Tomkins’ [1] notion that the opposition between humanistic and normative worldviews and ideologies is universal. However, much more research is needed to probe the claim of universality in a serious manner. At the end of the day, the U.S. and Sweden are both Western, individualistic, post-industrial democracies, and many of our participants, especially in Studies 1 and 4, were more educated and more left-leaning than the average person; they are not statistically representative of the U.S. and Swedish populations, let alone “mankind” in general [94].

Another obvious limitation of the present research is that it relied solely on cross-sectional data and cannot speak to issues of causality. Experimental and longitudinal evidence would be needed to isolate specific ideo-affective “resonances” [19] or “affinities” [95] between ideological worldviews and cognitive, affective, motivational, and behavioral inclinations. Only these sorts of methods could help to elucidate the stages in the development of the “love-affair,” as Tomkins [1, p. 389] put it, between psychological and ideological forces. Although personality traits, because of their relative stability, are often treated as causes of worldviews (e.g., [46, 96], they are by no means immutable [97], and it is quite conceivable that the adoption of specific worldviews shapes personality characteristics (e.g., see [98]).

It is therefore crucial that future research addresses reciprocal directions of causality [18] as well as the role of genetic covariation [99] and social environments that are conducive to humanistic vs. normative ways of thinking and behaving [100]. Research based on the dual process model suggests that causal relations between dangerous-world beliefs and RWA and between competitive-world beliefs and SDO are bidirectional [101]. Such findings are consistent with Tomkins’ [1, 19] theorizing, which rests upon a subtle understanding of the mutual affinities involving worldviews, political ideologies, and cultural contexts (see [30]) rather than a simpler notion that a single unidirectional causal chain applies uniformly across cultures, historical contexts, and social groups. For instance, it would also be useful to determine, in experimental and other contexts, whether humanists would be especially likely to advocate for social change when the status quo is framed as jeopardizing the rights or well-being of individuals, as Tomkins’ [1] work would suggest. Conversely, we would expect that normativists would be more open to social change when it is framed as necessary to protect the social system [102].

Finally, more work remains to be done to integrate the various psychological models of political ideology and to measure their components optimally. Although humanism and normativism may represent key elements of personal worldviews, they are not necessarily exhaustive [31], and the scales used to measure them may be susceptible to social desirability and response biases [68]. Our results also highlight the fact that there is a good deal of conceptual and empirical overlap involving constructs derived from different theories. Further work is needed to determine the extent to which concepts and variables from different approaches can be more fully integrated or distilled into a more parsimonious model—or whether they should be treated as complementary. Above all, it is important to spell out the psychological processes that are responsible for “elective affinities” between personality traits and ideological worldviews (see [95]). Future work is needed to determine the extent to which psychological models of ideology can be reconciled and integrated with biological theories that explain political preferences in terms of neurocognitive structures and functions [103] and embodied cognition [104], so as to produce a more complete account of personality and political orientation.

## Conclusion

More than half a century has passed since Tomkins [1, 19, 23] introduced polarity theory, which characterizes the familiar left-right ideological divide in terms of conflicts between humanistic and normative worldviews. Since the time of his writings, political psychology has flourished as a scientific sub-discipline in ways that Tomkins could never have imagined. Research in this area has illustrated vividly and comprehensively the effects of beliefs, opinions, values, emotions, motivations, goals, personal narratives, and dispositional characteristics on political behavior [4, 5, 11, 22, 42, 47, 51, 54, 95, 105–107]. It is no exaggeration to suggest that Tomkins was one of the very first psychologists to appreciate the profound resonances between psychological and ideological forms of human activity [15, 28]. At the same time, most of the insights crystallized in polarity theory—the cumulative pinnacle of Tomkins’ thinking about ideology, and the product of a herculean effort to grapple with the structural dynamics of left and right throughout different cultural and historical epochs—are largely lost on contemporary researchers.

Despite having dipped into relative obscurity, polarity theory is in fact more relevant today than ever before, given the heightened salience of individual and group differences in beliefs, values, and moral convictions and their apparent role in ideological polarization in society [2, 4, 13, 22, 105]). While most recent models focus on specific aspects of worldviews—such as lists of values or moral intuitions about the social world [21, 22, 107]—polarity theory provides a richer, more integrative analysis of the patterns of meaning, structure, and coherence that permeate the individual’s entire worldview, including core assumptions that underlie moral, political, scientific, and aesthetic judgments, among many other things.

Polarity theory reminds us of the basic existential dilemmas inherent in the human condition—the kinds of dilemmas that personal worldviews and political ideologies seek to resolve [4, 18]. What is human nature like, how should knowledge be pursued, how should emotions be regulated, how should society be governed, and how should we treat other people in our own society and in others? These are fundamental questions, and how each of us answers them speaks volumes about us as individuals as well as the social and cultural experiences to which we have been exposed.

In a sense, polarity theory represents a cry of protest against the myopic, fragmented state of much contemporary research in psychology, which is driven by ever-increasing demands for narrow professional specialization. Most ambitiously, polarity theory seeks to explain how it is possible that a single, left-right dimension can be meaningful and useful—when it comes to describing, explaining, and predicting human behavior across time, place, and myriad life domains [4, 20, 42, 48, 95, 106, 107]. According to Tomkins, humanistic and normative orientations epitomize contrasting ways of dealing with timeless, fundamental problems posed by individual existence and social life, including questions of autonomy and authority, freedom and conformity, creativity and discipline, equality and stratification, fairness and exploitation, and progress and tradition. Much as the opposing gravitational pulls of the moon and sun aggravate geological fault lines and contribute to devastating earthquakes, is it possible that there are reasonably strong magnetic psychological poles that produce frictions, clashes, conflicts, and divisions of a surprisingly intense ideological nature?

## Supporting information

S1 Appendix Scale items and parceling. This document contains a detailed description of the item parceling procedure used for structural equation modeling and the items contained in each parcel.
(PDF)

S2 Appendix Distinctness of theoretically related constructs. This document contains a description of results from preliminary analyses conducted to ascertain that theoretically related constructs were sufficiently distinct to be modeled in terms of different latent factors.
(PDF)

S3 Appendix Indirect relations. This document reports results of analyses of indirect paths from humanism and normativism to political orientation.
(PDF)

S1 Data Data files and codebook. This is a zip archive that contains all of the data sets in csv-format and a codebook.
(ZIP)
