# Supplementary material for: Rediscovering Tomkins’ polarity theory: Humanism, normativism, and the psychological basis of left-right ideological conflict in the U.S. and Sweden
Source: PLoS One. 2020 Jul 31;15(7):e0236627. doi: 10.1371/journal.pone.0236627 (PMC7394437; doi:10.1371/journal.pone.0236627)
Supplement: S1 Appendix — This document contains a detailed description of the item parceling procedure used for structural equation modeling and the items contained in each parcel. (PDF) [file pone.0236627.s001.pdf]

Item parceling

Humanism and Normativism

We used the facet-representative (or "shared uniqueness") strategy (Little, Rhemtulla, Gibson, & Schoemann, 2013) when parceling these two constructs, because they have theoretically specified facets. Both constructs have five facets—view of human nature, interpersonal attitude, attitude to affect, epistemology, and political values—and their relation varies across the facets. Confirmatory factor analyses using item-parceling on data from Swedish and U.S. samples have been presented in past research (Nilsson, 2014).

In general, we tried to follow Little's (2013) recommendation to strive for a just-identified model, with three indicators per latent construct. To achieve this goal, we aggregated (1) view of human nature and interpersonal attitude (correlation between them: r = .60 and .58 with the full eight-item facet-scales in Study 1) because humanism and normativism are negatively correlated in terms of both of these facets, and (2) epistemology and political values (correlation between them: r = .51 and .46), because humanism and normativism are not negatively correlated across these two facets (Nilsson, 2014). The model with these aggregated facets (three indicators per factor) fit the data well. We present results of confirmatory factor analysis below followed. We also present all of the items in Supplementary Tables 1 and 2. The 15-item short-scales are comprised of the first three items listed for each of the facets (see Nilsson, 2015 for more details on the scale).

Study 1

Humanism: Fit of the full hierarchical model with eight items per facet as indicators: $\chi^2(735)=1500.1$, $p < .001$, RMSEA = .052[.048, .056]. Factor loadings of facets: .78, .73, .82, .55, .83. Factor loadings of items: ≥ .27, ≤ .85.

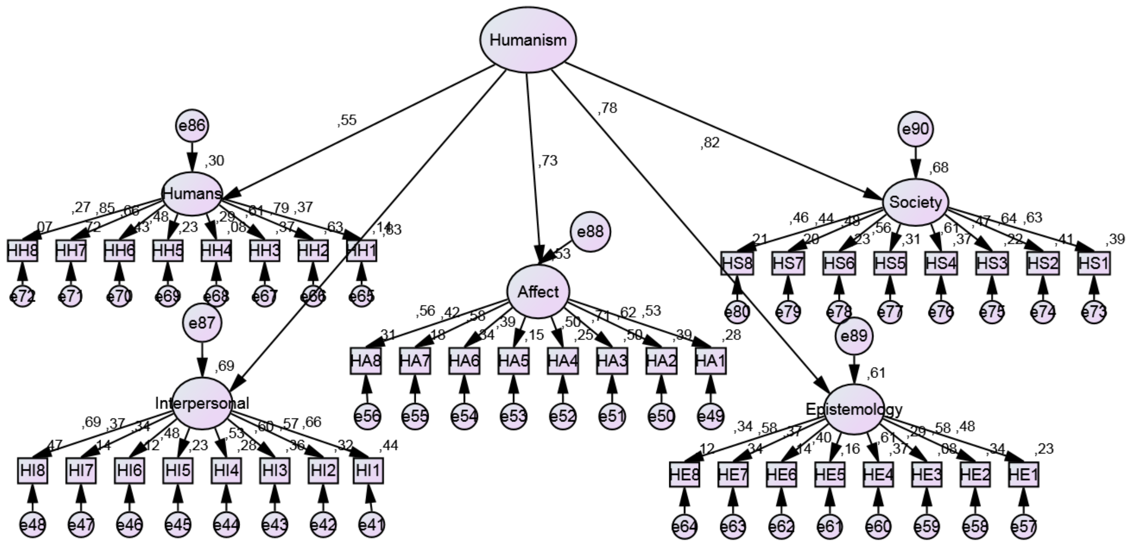

Normativism: Fit of the full hierarchical model with eight items per facet as indicators: $\chi^2(735)=1434.8$, $p < .001$, RMSEA = .050[.046, .054]. Factor loadings of facets: .74, .87, .72, .82, .67. Factor loadings of items: ≥ .30, ≤ .70.

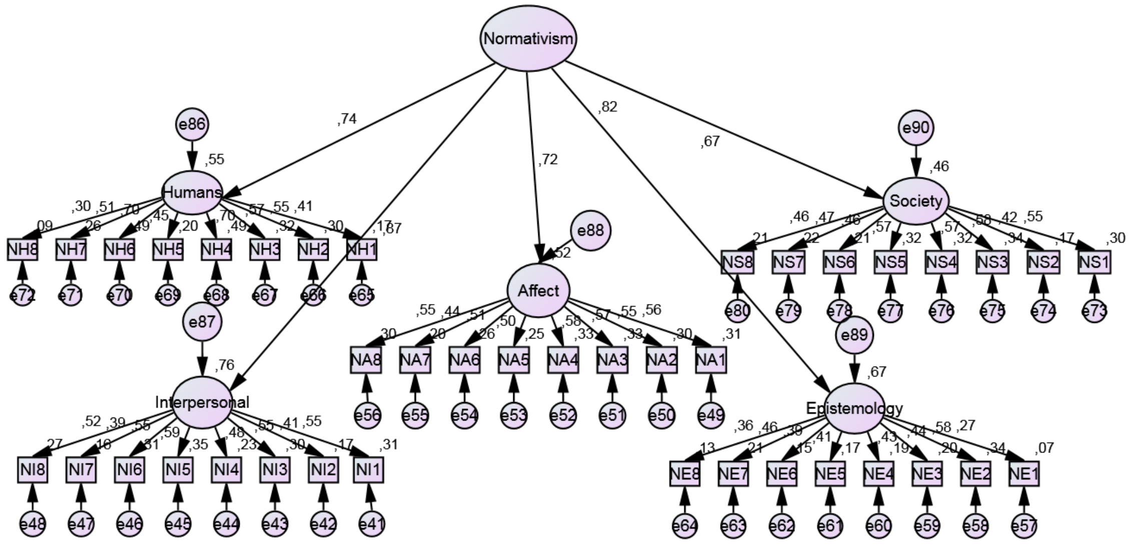

Combined model with all five facet-pairs: $\chi^2(29)=109.2$, $p < .001$, CFI = .932, RMSEA = .085[.068, .102], AIC = 181.2. Factor loadings, humanism: .56, .74, .64, .70, .65. Factor loadings, normativism: .69, .73, .66, .61, .64
Combined model with three facet-pairs: $\chi^2(5)=12.0$, $p = .035$, CFI = .989, RMSEA = .060[.015, .105], AIC = 56.0. Factor loadings, humanism: .64, .64, .85. Factor loadings, normativism: .78, .65, .73.

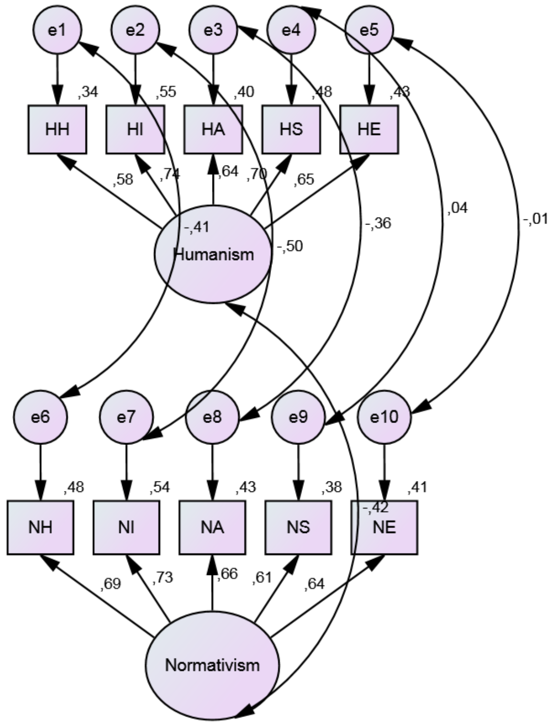

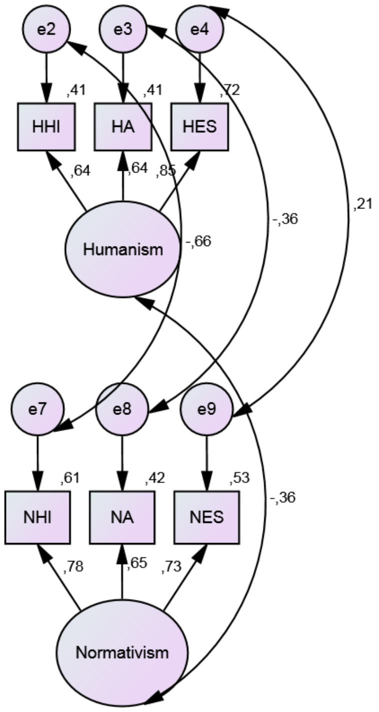

Study 2

Humanism: Fit of the full hierarchical model with three items per facet as indicators: $\chi^2(85)=305.6$, $p < .001$, RMSEA = .052[.048, .056]. Factor loadings of facets: .53, .86, .86, .72, .77. Factor loadings of items: .16, .65, .61 (political), .46, .88, .81 (human nature), .58, .79, .70 (affect), .76, .62, .71 (interpersonal), .43, .45, .81 (epistemology).

Normativism: Fit of the full hierarchical model with three items per facet as indicators: $\chi^2(85)=193.2$, $p < .001$, RMSEA = .060[.049, .072]. Factor loadings of facets: .45, .39, .48, .68, .89. Factor loadings of items: .52, .75, .67 (political), .64, .62, .71 (human nature), .63, .51, .57 (affect), .65, .72, .34 (interpersonal), .72, .48, .50 (epistemology).

Combined model with all five facet-pairs: $\chi^2(29)=175.2$, $p < .001$, CFI = .820, RMSEA = .120[.104, .138], AIC = 247.4. Factor loadings, humanism: .53, .72, .61, .50, .54. Factor loadings, normativism: .26, .26, .62, .49, .59.
Combined model with three facet-pairs: $\chi^2(5)= 14.1$, $p = .015$, CFI = .979, RMSEA = .072[.029, .118], AIC = 58.1. Factor loadings, humanism: .64, .67, .67. Factor loadings, normativism: .40, .54, .81.

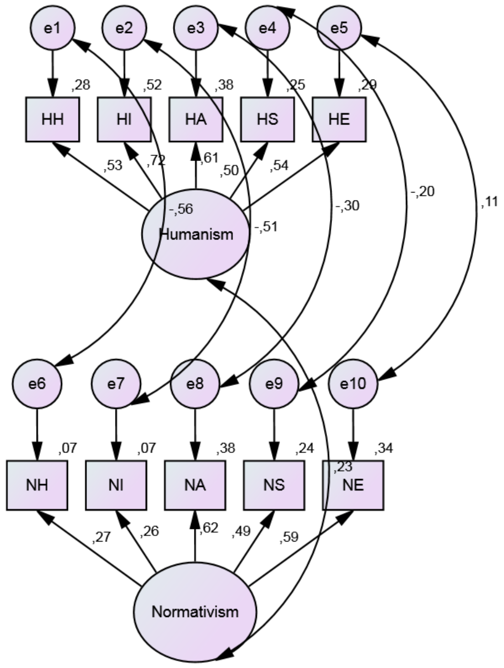

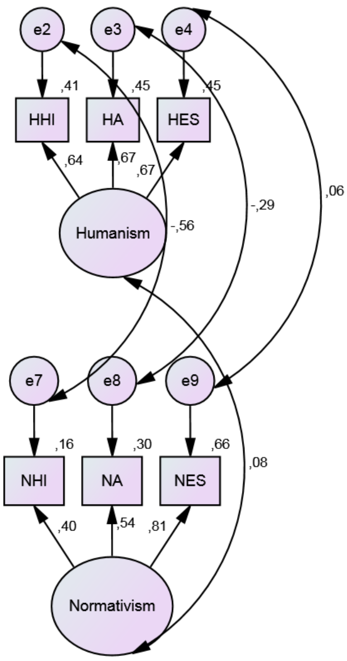

Study 3

Humanism: Fit of the full hierarchical model with three items per facet as indicators: $\chi^2(85)=334.5$, $p < .001$, RMSEA = .092[.082, .102]. Factor loadings of facets: .99, 1.03, .95, .68, .76. Factor loadings of items: .85, .46, .37 (political), .17, .50, .75 (human nature), .54, .64, .57 (affect), .87, .42, .56 (interpersonal), 1.01, .26, .16 (epistemology).

Normativism: Fit of the full hierarchical model with three items per facet as indicators: $\chi^2(85)=231.3$ $p < .001$, RMSEA = .070[.060, .081]. Factor loadings of facets: .70, .73, .65, .19, .67. Factor loadings of items: .61, .77, .62 (political), .52, .44, .54 (human nature), .54, .54, .59 (affect), .66, .52, .62 (interpersonal), .77, .67, .20 (epistemology).
Combined model with all five facet-pairs: $\chi^2(29)=145.5$, $p < .001$, CFI = .868, RMSEA =

.107[.090, .125], AIC = 217.5. Factor loadings, humanism: .62, .85, .65, .73, .49. Factor loadings, normativism: .46, .55, .52, .56, .22.
Combined model with three facet-pairs: $\chi^2(5)$= 27.5, p < .001, CFI = .952, RMSEA = .114[.074, .157], AIC = 71.5. Factor loadings, humanism: .68, .67, .83. Factor loadings, normativism: .43, .53, .71.

Study 4

Humanism: Fit of the full hierarchical model with three items per facet as indicators: $\chi^2(85)$=156.3, p < .001, RMSEA = .051[.038, .063]. Factor loadings of facets: .50, .95, .80, .52, .59. Factor loadings of items: .56, .38, .24 (political), .69, .83, .22 (human nature), .71, .67, .42 (affect), .52, .44, .71 (interpersonal), .24, .50, .47 (epistemology).

Normativism: Fit of the full hierarchical model with three items per facet as indicators: $\chi^2(85)$=239.4, p < .001, RMSEA = .074[.063, .086]. Factor loadings of facets: .33, .55, .67, .70, .75. Factor loadings of items: .77, .59, .65 (political), .70, .80, .31 (human nature), .48, .74, .63 (affect), .73, .61, .57 (interpersonal), .61, .65, .24 (epistemology).

Combined model with all five facet-pairs: $\chi^2(29)$=102.8, p < .001, CFI = .886, RMSEA = .088[.070, .107], AIC = 174.8. Factor loadings, humanism: .54, .70, .55, .51, .46. Factor loadings, normativism: .62, .63, .55, .44, .42.
Combined model with three facet-pairs: $\chi^2(5)$= 22.1, p < .001, CFI = .956, RMSEA = .102[.061, .147], AIC = 66.1. Factor loadings, humanism: .55, .62, .66. Factor loadings, normativism: .58, .63, .60.

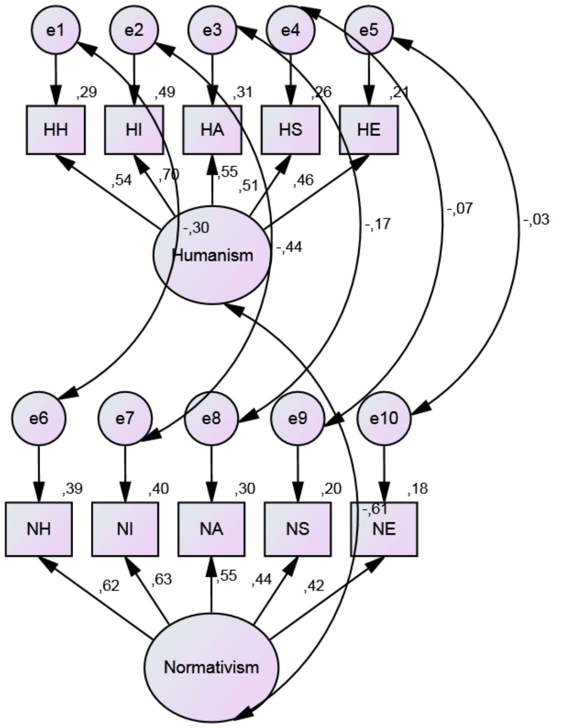

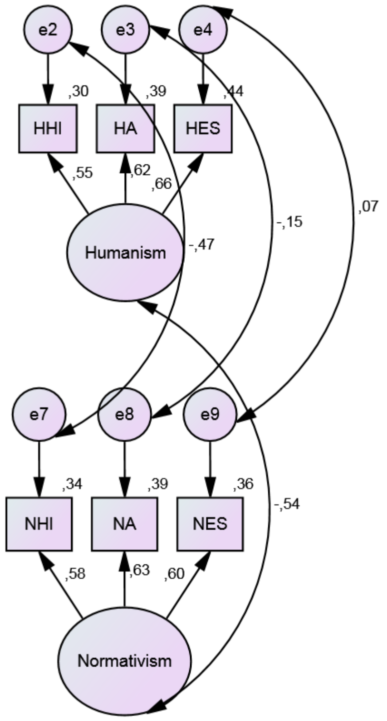

Supplementary Table 1. Humanism items.

Facet	Item
Human nature	All persons are in themselves valuable
	Human beings are basically good
	People are basically kind and helpful
	All human beings have an inner potential that they strive to realize
	Human beings are from the start good, even though bad circumstances can make them do bad things
	Although there is good and bad in people, humanity as a whole is basically good
	Human nature is basically good
	When people do good deeds it is almost always out of genuine compassion and care for others
Inter-personal	Human beings should be loved at all times, because they want and need to be loved
	Human beings should be treated with respect at all times
	When people are in trouble, they need help and should be helped
	Children must be loved so that they can grow up to be fine adults
	To assume that most people are well-meaning brings out the best in others
	Those who err should be forgiven
	No one has the right to humiliate another person
	All persons deserve to be loved
Attitude to affect	Feelings are the most important aspect of being human, because they give our lives meaning
	You need to be open to your feelings so that you can learn from them and understand who you are
	You must always leave yourself open to your own feelings – alien as they may sometimes seem
	There is a unique avenue to reality through the feelings, even when they seem alien
	The changeableness of human feelings makes life more interesting
	People should try to look inward to understand and accept their feelings as they are
	Feelings provide the most important guidance to a person's decisions
	You should go with you feelings so that you do not have to look back in regret for holding back from what you really wanted
Epistem-ology	The main purpose of education should be to enable the young to discover and create novelty
	Creativity and curiosity are the most important tools in the search for knowledge
	The important thing in science is to strike out into the unknown – right or wrong
	Personal imagination and understanding is crucial to the pursuit of knowledge
	A scientist must rely on creativity and intuition
	Learning must always start from your personal interests and experiences
	Great achievements require first of all great imagination
	Personal experiences can provide insights about reality that science cannot explain
Political values	The most important purpose of society is to protect people's rights, freedoms, and dignity
	The most important goal for a society is to make sure that all its members have a chance to lead a good life
	It is necessary to break the laws and rules of society when these lead to unfair treatment of some people
	The most important function of the government is to make sure people are treated in a just and dignified way
	Promotion of the welfare of the people is the most important function of a government
	We have to question the rules of the society when the well-being of individuals is threatened
	Society should encourage people to express themselves and follow their own desires
	Societies that violate human freedoms and rights must be vigorously questioned

Supplementary Table 2. Normativism items.

Facet	Item
Human nature	When people do good deeds, it is almost always out of an expectation to receive something in return
	The bad people in the world outnumber the good people
	People don't really care what happens to the next person
	People are naturally unfriendly and unkind
	Human beings are from the start primitive and egoistic animals that must be disciplined by society
	Human beings are basically evil
	Juvenile delinquency is simply a reflection of the basic evil in human beings – it has always existed in the past and it always will
	A person can only realize her-/himself by attaining external ideals
Inter-personal	Human beings should be treated with respect only when they deserve respect
	When people are in trouble, they should help themselves and not depend on others
	Human beings should be loved only when they have acted so that they deserve to be loved
	Some people respond only to punishment or the threat of punishment
	It is necessary to be hard and cold hearted toward other people when they deserve it
	Some people can only be changed by humiliating them
	When a person feels sorry for himself he should really feel ashamed of himself
	To assume that most people are well-meaning is asking for trouble
Attitude to affect	Human beings would be lost without reason, because feelings cannot be trusted
	Feelings must be controlled by reason, because they can make you do stupid things
	Feelings are often an obstacle to seeing how things really are
	You need to be wary of feelings, because they can hurt you and make you feel miserable
	If sanity is to be preserved, you must guard yourself against the intrusion of feelings which are alien to your nature
	The changeableness of human feelings is a weakness in human beings
	Going with your feelings often makes you unhappy in the long run
	There is no surer road to insanity than surrender to the feelings, particularly those which are alie to the self
Epistem-ology	The most important task for a scientist is to collect facts about reality through objective observation
	Reason has to be continually disciplined and corrected by reality and hard facts
	To observe objectively and describe in a neutral language is crucial to the pursuit of knowledge
	The trouble with theorizing is that it leads people away from the facts and substitutes opinions fo truth
	Observing the world accurately enables human beings to separate reality from imagination
	Discipline and rigour are the most important tools in the search for knowledge
	Imagination leads people into self-deception and delusions
	Education should focus on facts rather than theories
Political values	The maintenance of law and order is the most important duty of any government
	People who commit crimes must be punished severely so that they are deterred from repeating th crime
	A society must enforce its laws and rules strictly in order not to deteriorate
	In order for society to work, there must be clear and fixed rules, and punishment for transgressions
	The most important function of society is to keep people's destructive impulses under control with laws and rules
	Anger should be directed at those revolutionaries who undermine law and order
	Society should not encourage deviant and unwholesome activities
	It is often necessary to punish people severely in order to get them to conform to the social order

HEXACO

The hierarchical structure of the HEXACO traits is well-established (Lee & Ashton, 2016). Each trait can be subdivided into four distinct facets. This model proved to fit our data well. We therefore used a facet-representative strategy when parceling the HEXACO traits that we measured in Study 4 (openness, honesty-humility, and emotionality). Results of confirmatory factor analyses are shown below. The full HEXACO inventory and scoring keys are available online (hexaco.org/hexaco-inventory).

Openness

One-factor model with items as indicators: $\chi^2(35)=145.9$, $p < .001$, RMSEA = .098[.082, .115], AIC = 205.9. Factor loadings: .53, .44, .32, .32, .43, .50, .44, .60, .36, .57.

Hierarchical model with four facets (creativity, unconventionality, aesthetic appreciation, and inquisitiveness) with two or three items each as indicators: $\chi^2(31)=75.5$, $p < .001$, RMSEA = .064[.045, .083], AIC = 141.5. Factor loadings of the facets: .79, .70, .74, .74. Factor loadings of items: .67, .42, .55 (creativity), .37, .69, .58 (unconventionality), .75, .63 (aesthetic appreciation), .48, .60 (inquisitiveness).

Honesty-humility

One-factor model with items as indicators: $\chi^2(35)=172.$, $p < .001$, RMSEA = .127[.110, .146], AIC = 226.1. Factor loadings: .36, .34, .38, .38, .43, .67, .49, .26, .51.

Hierarchical model with four facets (fairness, sincerity, greed avoidance, and modesty) with two or three items each as indicators: $\chi^2(31)=58.2$, $p < .001$, RMSEA = .068[.046, .090], AIC = 120.2. Factor loadings of the facets: .41, .66, .89, .55. Factor loadings of items: .82, .62 (fairness), .54, .48, .45 (sincerity), .54, .80 (greed avoidance), .93, .32 (modesty).

Emotionality

One-factor model with items as indicators: $\chi^2(35)=120.6$, $p < .001$, RMSEA = .086[.069, .103], AIC = 180.6. Factor loadings: .46, .41, .44, .72, .64, .37, .46, .54, .41, .34.

Hierarchical model with four facets (fearfulness, anxiety, sentimentality, and dependence) with two or three items each as indicators: $\chi^2(31)=54.9$, $p < .001$, RMSEA = .048[.026, .069], AIC = 122.9. Factor loadings of the facets: .80, .78, .78, .74. Factor loadings of items: .47, .55, .58 (fearfulness), .70, .83 (anxiety), .71, .52, .48 (sentimentality), .46, .57 (dependence).

Moral intuitions

According to Moral Foundations Theory, there are at least five distinct moral foundations, and these foundations can be divided into two main categories: "individualizing" (i.e., liberal) and "binding" (i.e., conservative) moral intuitions (Graham et al., 2013). We therefore used a facet-representative approach here as well, dividing the liberal moral intuitions into harm and fairness and the conservative intuitions into loyalty, authority, and purity (see also Nilsson & Erlandsson, 2015). Results of confirmatory factor analyses are shown below. The Moral Foundations Questionnaire is available in full online (moralfoundations.org/questionnaires).

Liberal moral intuitions

One-factor model with items as indicators: $\chi^2(54)=211.2$, $p < .001$, RMSEA = .094[.081, .107], AIC = 283.2. Factor loadings: .48, .59, .35, .47, .30, .23, .60, .47, .42, .56, .51, .40.

Hierarchical model with two facets (harm/care and fairness) with six items each as indicators: $\chi^2(53)=168.7$, $p < .001$, RMSEA = .081[.068, .095], AIC = 242.7. Factor loadings of the facets: .73 and .93. Factor loadings of items: .43, .64, .58, .54, .33, .25 (harm/care), .46, .47, .65, .62, .59, .34 (fairness).

Conservative moral intuitions

One-factor model with items as indicators: $\chi^2(135)=559.3$, $p < .001$, RMSEA = .097[.089, .106], AIC = 667.3. Factor loadings: .45, .52, .53, .29, .37, .11, .65, .46, .52, .58, .37, .31, .59, .56, .36, .49, .52, .36.

Hierarchical model with three facets (loyalty, authority, and purity) with six items each as indicators: $\chi^2(133)=533.8$, $p < .001$, RMSEA = .092[.083, .100], AIC = 615.8. Factor loadings of the facets: .78, .88, .87. Factor loadings of items: .69, .70, .43, .17, .33, .13 (loyalty), .60, .44, .73, .63, .37, .36 (authority), .36, .57, .59, .37, .63, .58 (purity).

Components of the dual-process model and the ideology as motivated social cognition model

All of the constructs that make up the dual-process model and the model of ideology as motivated social cognition are typically thought of as unidimensional. Although recent work has sought to disentangle a number of different facets of right-wing authoritarianism (Duckitt, Bizumic, Krauss, & Heled, 2010) and social dominance orientation (Ho et al., 2012, 2015), we used adapted versions of the original scales, which are the ones that were used in the development and evaluation of the dual-process model. We therefore decided to primarily use a balancing approach to parceling for all of these constructs, combining strongly loading items with weakly loading items in each parcel to balance out the factor loadings of the parcels and thereby optimize the measurement model (Little et al., 2013), insofar as the constructs showed sufficient evidence of the presence of a general factor in our data.

We investigated the factor-structure of the scales by computing hierarchical omega reliabilities based on item-level exploratory factor analysis with Schmid Leiman transformations and scrutinizing the factor loadings (using the "psych"-package version 1.8.4 in R version 3.0; Revelle, 2018). The hierarchical omega indicates the proportion of correlations among items that can be accounted for by one general factor. Although the hierarchical omega reliabilities varied (see Supplementary Tables 3-10 below), they were generally higher than those found in scales with underlying facets (e.g., the ω_h ranged from .35 to .54 for the HEXACO and moral intuitions scales in our data), only in one case did more than one item fail to correlate above .20 with the general factor (in Study 1, three RWA items failed to correlate with the general RWA factor), and there was usually no theoretically meaningful facet-structure. Lack of structural homogeneity commonly stemmed from the clustering of reversed and non-reversed items (e.g., in RWA and SDO).

There was, however, one exception. Resistance to change had low hierarchical omega reliabilities ($\leq$.50) in Study 1 and Study 4 (see Supplementary Table 7) and there was a potentially theoretically meaningful facet-structure. Items measuring a preference for slow over radical change tended to cluster together, as did items measuring traditionalism. We placed the items that most clearly represented these facets in two distinct parcels and the rest of the items (which were either not clear examples of one of the two facets or blended them) in a third parcel (see Supplementary Table 7), similar to correlational and radial parceling (Little et al., 2013). The relations between resistance to change and other constructs in our models were generally homogeneous across the facets (see Supplementary Table 11), and the model fit was not reduced with this parceling compared to a balancing approach to parceling in Study 1 or Study 4.

In Study 2, the hierarchical omega reliability of resistance to change was much higher. We therefore considered both using the same parceling as in Study 1 and Study 4 and using a balancing approach to parceling, which is appropriate for unidimensional scales. The parameter estimates proved to be almost exactly the same regardless of which parceling was used, but the parceling based on the balancing approach produced better model fit (χ^2[87] = 306.2, CFI = .915, RMSEA = .085[.075, .095], AIC = 435.2 compared to χ^2[87] = 396.4, CFI = .876, RMSEA = .101[.091, .111], AIC = 526.4). We therefore report estimates based on the balancing approach in Study 2.

We used a balancing approach also for the rest of the constructs that comprise the dual process model and the model of ideology as motivated social cognition. We started from the

item-total correlations, placing the item with the highest item-total correlation in the first parcel, the one with the second highest in the second parcel, and so on (Little et al., 2013). This technique does of course not guarantee that the parcels will have the same loadings on the latent factor. In cases for which the difference between the loadings was not negligible, we adjusted the parceling by exchanging items between the parcels to make the factor loadings more balanced.

We used a single item measuring ideological self-placement as the outcome variable in the majority of our analyses. But in Study 2 we also constructed a latent factor to represent ideology, using ideological self-placement as one of the indicators and two parcels with items measuring issue-based preferences as two additional indicators. We divided the issue-based preferences into parcels with same the balancing approach that we used for other constructs.

All of the items, the hierarchical omega reliabilities, the item-total-correlations, and the parceling are shown in Supplementary Tables 3-10.

Supplementary Table 3. Right-wing authoritarianism: item parceling.

Item	Study 1 ω_h = .67		Study 2 ω_h = .59		Study 3 ω_h = .68	
	Parcel	Item-total	Parcel	Item-total	Parcel	Item-total
1. Many good people challenge the state, criticize the church and ignore ‘‘the normal way of living’’. (reversed)	1	.37	3	.41	1	.35
2. There are many radical, immoral people trying to ruin things; the society ought to stop them.	3	.36	2	.65	2	.38
3. People ought to put less attention to the Bible and religion, instead they ought to develop their own moral standards. (reversed)	2	.55	1	.63	3	.11
4. Facts show that we have to be harder against crime and sexual immorality, in order to uphold law and order.	3	.36	3	.70	2	.56
5. Our forefathers ought to be honored more for the way they have built our society, at the same time we ought to put an end to those forces destroying it.	3	.11	2	.59	1	.57
6. If the society so wants, it is the duty of every true citizen to help eliminate the evil that poisons our country from within.	2	.22	1	.51	2	.41
7. Our society would be better off if we showed tolerance and understanding for untraditional values and opinions. (reversed)	1	.21	3	.55	3	.41
8. It would be best if newspapers were censored so that people would not be able to get hold of destructive and disgusting material.	2	.51	1	.45	3	.41
9. The situation in the society of today would be improved if troublemakers were treated with reason and humanity. (reversed)	1	.26	2	.43	2	.24
10. God's laws about abortion, pornography and marriage must be strictly followed before it is too late, violations must be punished.	1	.65	2	.73	3	.54
11. The society needs to show openness towards people thinking differently, rather than a strong leader, the world is not particularly evil or dangerous. (reversed)	2	.34	2	.52	1	.49
12. Our country needs free thinkers, who will have the courage to stand up against traditional ways, even if this upsets many people. (reversed)	3	.49	1	.60	3	.59
13. The ‘‘old-fashioned ways’’ and ‘‘old-fashioned values’’ still show the best way to live.	1	.53	1	.76	2	.55
14. It is better to accept bad literature than to censor it. (reversed)	3	.50	3	.53	1	.24
15. Our country needs a powerful leader, in order to destroy the radical and immoral currents prevailing in society today.	2	.41	3	.69	1	.54

Supplementary Table 4. Social dominance orientation: item parceling.

Item	Study 1 $\omega_h = .61$		Study 2 $\omega_h = .67$		Study 3 $\omega_h = .63$	
	Parcel	Item-total	Parcel	Item-total	Parcel	Item-total
1. We would have fewer problems if we treated people more equally. (reversed)	2	.52	2	.67	1	.49
2. Inferior groups should stay in their place.	3	.51	1	.66	1	.30
3. To get ahead in life, it is sometimes necessary to step on other groups.	1	.42	2	.55	3	.45
4. We should do what we can to equalize conditions for different groups. (reversed)	3	.46	2	.68	2	.46
5. In getting what you want, it is sometimes necessary to use force against other groups.	2	.45	3	.54	2	.62
6. Some groups of people are simply inferior to others.	1	.55	1	.70	1	.63
7. Group equality should be our ideal. (reversed)	1	.51	3	.66	3	.50
8. We should strive to make incomes as equal as possible. (reversed)	2	.38	1	.48	2	.40

Supplementary Table 5. Dangerous-world beliefs: item parceling.

Item	Study 1 ω_h = .54		Study 2 ω_h = .68		Study 3 ω_h = .64	
	Parcel	Item-total	Parcel	Item-total	Parcel	Item-total
1. Any day now chaos and anarchy could erupt around us. All signs are pointing to it.	3	.51	1	.70	2	.48
2. There are many dangerous people in our society who will attack someone out of pure meanness, for no reason at all.	3	.37	2	.59	3	.50
3. Despite what one hears about "crime on the street", there probably isn't any more now than there ever has been. (reversed)	3	.48	3	.58	3	.47
4. If a person takes a few sensible precautions, nothing bad is likely to happen to him or her; we do not live in a dangerous world. (reversed)	2	.38	1	.49	1	.36
5. Every day as society becomes more lawless and bestial; a person's chances of being robbed, assaulted, and even murdered go up and up.	2	.50	1	.77	1	.65
6. My knowledge and experiences tell me that the social world we live in is basically a safe, stable, and secure place in which most people are fundamentally good. (reversed)	1	.43	1	.59	3	.34
7. It seems that every year there are fewer and fewer truly respectable people, and more and more persons with no morals at all who threaten everyone else.	1	.55	2	.67	1	.47
8. My knowledge and experience tell me that the social world we live in is basically a dangerous and unpredictable place, in which good, decent, and moral people's values and way of life are threatened and disrupted by bad people.	1	.50	3	.74	2	.57
9. The "end" is not near. People who think that earthquakes, wars, and famines mean God might be about to destroy the world are being foolish. (reversed)	1	.34	2	.74	1	.24
10. Although it may appear that things are constantly getting more dangerous and chaotic, it really isn't so. Every era has its problems, and a person's chances of living a safe, untroubled life are better today than ever before. (reversed)	2	.52	3	.67	2	.42

Supplementary Table 6. Competitive-world beliefs: item parceling.

Item	Study 1 ω_h = .66		Study 2 ω_h = .75		Study 3 ω_h = .59	
	Parcel	Item-total	Parcel	Item-total	Parcel	Item-total
1. Winning is not the first thing; it's the only thing.	3	.59	1	.64	2	.56
2. The best way to lead a group under one's supervision is to show them kindness, consideration, and treat them as fellow workers, not as inferiors. (reversed)	2	.48	1	.49	1	.35
3. If it's necessary to be cold blooded and vengeful to reach one's goals, then one should do it.	1	.62	3	.71	3	.54
4. Life is not governed by the "survival of the fittest." We should let compassion and moral laws be our guide. (reversed)	1	.50	2	.39	3	.45
5. Money, wealth, and luxury are what really count in life.	1	.56	2	.67	2	.53
6. It is much more important in life to have integrity in your dealings with others than to have money and power. (reversed)	3	.50	3	.47	1	.26
7. It's a dog eat dog world where you have to be ruthless at all times.	2	.60	1	.61	1	.58
8. You know that most people are out to "screw" you; so you have to get them first when you get a chance.	2	.56	2	.63	1	.54
9. My knowledge and experience tells me that the social world we live in is basically a "competitive jungle" in which the fittest survive and succeed; power, wealth, and winning are everything; and might is right.	3	.44	3	.40	3	.40
10. We can make a society based on unselfish cooperation, sharing, and people generously helping each other, and not on competition and acquisitiveness. (reversed)	1	.41	1	.25	2	.44

Supplementary Table 7. Resistance to change: item parceling.

Item	Study 1 ω_h = .44		Study 2 ω_h = .71		Study 4 ω_h = .49	
	Parcel	Item-total	Parcel	Item-total	Parcel	Item-total
1. Young people sometimes get rebellious ideas, but as they grow up they ought to get over them and settle down.	3	.38	2	.59	3	.41
2. This country would be better off if there were more emphasis on traditional family ties.	1	.53	1	.76	1	.52
3. Our customs and national heritage are the things that have made us great, and some people should show greater respect for them.	1	.40	1	.69	1	.43
4. If you start changing things very much, you often end up making them worse.	2	.46	1	.52	2	.34
5. Changing any institution (e.g., government, religion, business) is risky, so it is better to change at a slow than a rapid pace.	2	.33	1	.39	2	.47
6. If something is wrong with an institution (e.g., government, religion, business), it is necessary to make immediate changes. (reversed)	2	.12	2	.15	2	.21
7. The answers for today's questions can often be found in the traditions and customs of the past.	3	.28	2	.74	3	.39
8. Looking back, life was much better in the past.	1	.38	2	.60	1	.32
9. The ''old-fashioned ways'' and ''old-fashioned values'' still show the best way to live.	3	.48	3	.82	3	.55
10. Our society would be better off if we showed tolerance and understanding for untraditional values and opinions. (reversed)	1	.12	3	.53	1	.32
11. If something grows up over a long time, there will usually be much wisdom in it.	3	.33	3	.24		-

Supplementary Table 8. Preference for equality: item parceling.

Item	Study 1 ω_h = .58		Study 2 ω_h = .64		Study 4 ω_h = .70	
	Parcel	Item-total	Parcel	Item-total	Parcel	Item-total
1. Companies should be held accountable for promoting racial diversity and a tolerant atmosphere in their offices and corporate culture.	3	.38	1	.60	1	.62
2. The government should take more measures to eliminate economic disparities between men and women who are doing the same work.	2	.39	1	.57	1	.52
3. It is the responsibility of the government to take care of people who can't take care of themselves.	3	.47	3	.54	2	.53
4. We need to take care of the poor and disadvantaged before helping the rest of the country.	1	.63	2	.64	3	.54
5. We should find ways to help others less fortunate than ourselves.	3	.51	2	.69	3	.57
6. Prosperous nations have a moral obligation to share some of their wealth with poor nations.	2	.51	3	.52	2	.61
7. Whether we like it or not, some people are just more worthy than others. (reversed)	1	.34	2	.33	1	.33
8. Large fortunes and estates should be taxed fairly heavily over and above income taxes.	1	.40	1	.48		-
9. A person should always be concerned about the well-being of others.	2	.50	3	.59		-

Supplementary Table 9. System justification: item parceling.

Item	Study 1 ω_h = .66		Study 2 ω_h = .79		Study 4 ω_h = .65	
	Parcel	Item-total	Parcel	Item-total	Parcel	Item-total
1. In general, I find society to be fair.	1	.60	2	.73	1	.72
2. In general, the American political system operates as it should.	2	.69	1	.64	3	.65
3. American society needs to be radically restructured. (reversed)	1	.39	1	.42	2	.59
4. The United States is the best country in the world to live in.	3	.40	1	.43	2	.36
5. Most policies serve the greater good.	3	.53	2	.54	2	.62
6. Everyone has a fair shot at wealth and happiness.	3	.50	3	.57	3	.51
7. Our society is getting worse every year. (reversed)	2	.24	3	.13	1	.49
8. Society is set up so that people usually get what they deserve.	2	.51	3	.58	3	.61

Supplementary Table 10. Conservative issue preferences.

	ω_h = .58	
	Parcel	Item-total
1. Abortion (reversed)	2	.52
2. Welfare benefits (reversed)	1	.42
3. Limited government	1	.49
4. Military and national security	1	.56
5. Religion	2	.58
6. Gun ownership	1	.53
7. Traditional marriage	2	.64
8. Traditional values	1	.75
9. Fiscal responsibility	1	.42
10. Business	2	.47
11. The family unit	1	.50
12. Patriotism	2	.53
13. Labor unions (reversed)	1	.33
14. Gay marriage (reversed)	2	.59
15. Affirmative action (reversed)	2	.26
16. The death penalty	2	.41

Supplementary Table 11. Correlations between resistance to change parcels and other constructs.

	Parcel 1 (traditionalism)	Parcel 2 (preference for slow change)	Parcel 3 (remaining items)
**Study 1**			
Left-right self-placement	.38***	.32***	.40***
Preference for equality	-.20**	-.27***	-.28***
System justification	12	22**	.25***
Humanism	-.12	-.11	-.07
Normativism	.22**	.20**	.27***
**Study 2**			
Left-right self-placement	.65***	.40***	.58***
Conservative issue preferences	.78***	.45***	.73***
Preference for equality	-.31***	-.38***	-.15**
System justification	.16**	.32***	.26***
Humanism	-.10#	-.26***	.08
Normativism	.36***	.28***	.35***
**Study 4**			
Left-right self-placement	.47**	.35***	.48***
Preference for equality	-.40***	-.32***	-.41***
System justification	.42***	.40***	.42***
Humanism	-.18***	-.22***	-.22***
Normativism	.41***	.26***	.44***
Liberal moral intuitions	-.25***	-.26***	-.31***
Conservative moral intuitions	.48***	.28***	.44***
Openness	-.28***	-.18***	-.23***
Honesty-humility	-.24***	-.18***	-.25***
Emotionality	-.04	-.06	-.03

Note. # $p \leq .10$, *, $p \leq .05$, ** $p \leq .01$, *** $p \leq .001$.

References

Duckitt, J., Bizumic, B., Krauss, S. W., & Heled, E. (2010). A tripartite approach to right-wing authoritarianism: The authoritarianism-conservatism-traditionalism model. *Political Psychology, 31*(5), 685-715.

Graham, J., Haidt, J., Koleva, S., Motyl, M., Iyer, R., Wojcik, S. P., & Ditto, P. H. (2013). Moral foundations theory: The pragmatic validity of moral pluralism. *Advances in Experimental Social Psychology, 47*, 55-130.

Ho, A., K., Sidanius, J., Pratto, F., Levin, S., Thomsen, L., Kteily, N., & Sheehy-Skeffington, J. (2012). Social dominance orientation: Revisiting the structure and function of a variable predicting social and political attitudes. *Personality and Social Psychology Bulletin*, *38*(5), 583-606.

Ho, A. K., Sidanius, J., Kteily, N., Sheehy-Skeffington, J., Pratto, F., Henkel, K. E., . . . Stewart, A. L. (2015). The nature of social dominance orientation: Theorizing and measuring preferences for intergroup inequality using the new SDO_7 scale. *Journal of Personality and Social Psychology, 109*(6), 1003-1028.

Lee, K. & Ashton, M. C. (2016). Psychometric properties of the HEXACO-100. *Assessment*, *25*(5), 543-556.

Little, T. D. (2013). *Longitudinal structural equation modeling*. New York, NY: Guilford Press.

Little, T. D., Rhemtulla, M., Gibson, K., & Schoemann, A. M. (2013). Why the items versus parcels controversy needn't be one. *Psychological Methods*, *18*(3), 285-300.

Nilsson, A. (2014). Humanistic and normativistic worldviews: Distinct and hierarchically structured. *Personality and Individual Differences*, *64*, 135-140.

Nilsson, A. (2015). Humanism and normativism facet scales and short scales. *Lund Psychological Reports*, *15*, 1-16.

Nilsson, A., & Erlandsson, A. (2015). The moral foundations taxonomy: Structural validity and relation to political ideology in Sweden. *Personality and Individual Differences*, *76*, 28-32.

Revelle, W. (2018). psych: Procedures of personality and psychological research. Nortwestern University, Evanston, Illinois, USA. https://CRAN.R-project.org/package=psych
