# Supplementary material for: Rediscovering Tomkins’ polarity theory: Humanism, normativism, and the psychological basis of left-right ideological conflict in the U.S. and Sweden
Source: PLoS One. 2020 Jul 31;15(7):e0236627. doi: 10.1371/journal.pone.0236627 (PMC7394437; doi:10.1371/journal.pone.0236627)
Supplement: S2 Appendix — This document contains a description of results from preliminary analyses conducted to ascertain that theoretically related constructs were sufficiently distinct to be modeled in terms of different latent factors. (PDF) [file pone.0236627.s002.pdf]

Distinctness of theoretically related constructs

Confirmatory factor analyses

We investigated the extent to which having two theoretically related constructs represented by one common latent factor rather than separate latent factors would reduce model fit. These tests were performed in AMOS with the item parceling procedure described in Appendix 1.

We investigated whether

1. preference for equality and resistance to change are reducible to a common factor,
2. humanism, normativism, preference for equality, and resistance to change are reducible to a two-factor structure integrating humanism with preference for equality and normativism with resistance to change,
3. humanism, normativism, SDO, and RWA are reducible to a two-factor structure integrating humanism with (low) SDO and normativism with RWA
4. humanism, normativism, competitive-world belief, and dangerous-world belief are reducible to a two-factor structure integrating humanism with (low) competitive-world belief and normativism with dangerous-world belief

We also considered possible *post hoc* three factor-models. In every case, trying to reduce theoretically related constructs to a common factor produced substantial decreases in model fit. The main results are shown in Supplementary Table 1 below.

Supplementary Table 1. Fit indices for confirmatory factor analyses of conceptually overlapping constructs.

	χ^2	*df*	*p*	$p(\Delta\chi^2)$	CFI	RMSEA [95% CI]	AIC
Preference for equality and resistance to change							
Study 1 (U.S.)							
One-factor model	108.3	9	<.001		.628	.236[.197, .277]	144.3
Two-factor model	12.26	8	.14	<.001	.984	.052[.000, .106]	50.26
Study 2 (U.S.)							
One-factor model	296.0	9	<.001		.695	.304[.275, .334]	332.0
Two-factor model	29.09	8	<.001	<.001	.978	.087[.055, .123]	67.09
Study 4 (Sweden)							
One-factor model	120.0	9	<.001		.812	.193[.163, .225]	156.0
Two-factor model	9.88	8	.27	<.001	.997	.027[.000, .073]	47.88
Humanism, normativism, preference for equality, and resistance to change							
Study 1 (U.S.)							
Two-factor model	258.7	50	<.001		.689	.145[.128, .163]	338.7
Four-factor model	69.14	45	.001	<.001	.964	.052[.025, .075]	159.1
Study 2 (U.S.)							
Two-factor model	311.9	50	<.001		.845	.123[.110, .137]	391.9
Four-factor model	168.2	45	<.001	<.001	.927	.089[.075, .104]	258.2
Study 4 (Sweden)							
Two-factor model	189.6	50	<.001		.887	.092[.078, .106]	269.6
Four-factor model	121.8	45	<.001	<.001	.938	.072[.057, .087]	211.8
Humanism, normativism, right-wing authoritarianism, and social dominance orientation							
Study 1 (U.S.)							
Two-factor model	451.4	50	<.001		.769	.145[.133, .157]	531.4
Four-factor model	170.8	45	<.001	<.001	.928	.085[.072, .099]	260.8
Study 2 (U.S.)							
Two-factor model	404.8	50	<.001		.837	.143[.131, .157]	484.8
Four-factor model	193.4	45	<.001	<.001	.932	.098[.084, .112]	283.8
Study 3 (Sweden)							
Two-factor model	496.5	50	<.001		.683	.158[.145, .170]	576.5
Four-factor model	192.3	45	<.001	<.001	.896	.095[.082, .110]	282.3
Humanism, normativism, dangerous- and competitive-world beliefs							
Study 1 (U.S.)							
Two-factor model	429.6	50	<.001		.774	.141[.129, .153]	509.6
Four-factor model	116.6	45	<.001	<.001	.957	.064[.050, .079]	206.6
Study 2 (U.S.)							
Two-factor model	554.1	50	<.001		.744	.171[.158, .184]	634.1
Four-factor model	285.3	45	<.001	<.001	.878	.124[.111, .138]	375.3
Study 3 (Sweden)							
Two-factor model	505.7	50	<.001		.676	.159[.147, .172]	585.7
Four-factor model	133.7	45	<.001	<.001	.937	.074[.060, .089]	223.7

$p(\Delta\chi^2)$ = *p*-value of the χ^2 difference test; CFI = Comparative Fit Index; RMSEA = Root Mean Square Error of Approximation; AIC = Akaike Information Criterion.

Exploratory factor analyses

To provide a more detailed look at the overlap and distinctness of theoretically related constructs in our models, we also report results from exploratory item-based hierarchical factor analyses performed with oblimin rotation, Schmid Leiman transformations, and the Minimum Residual method (retrieved through the "omega" function in the "psych"-package version 1.8.4 in R version 3.0; Revelle, 2018), along with fit statistics (RMSEA with 90% confidence intervals). We used the same number of factors as the number of theorized scales, unless a larger number of factors helped to clarify the factor structure. For humanism and normativism we used only the 30 items that were included in all of the studies.

Resistance to change and acceptance of inequality

Study 1

The factor structure of both scales is shown in the figure below. One reversed resistance to change item loaded on the preference for equality factor, which indicates the possible existence of a method factor in this case and others (in total, 90% of the items performed *completely* as predicted, i.e. loading only on the expected factor or factors).

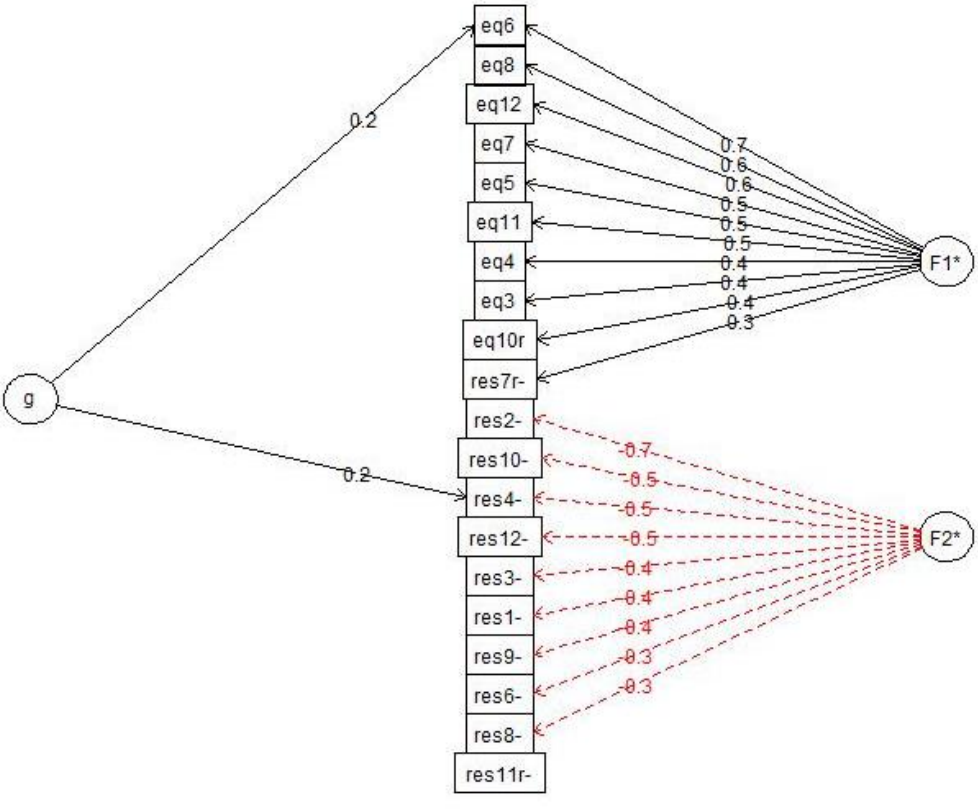

One general factor: χ^2 (170) = 1361.1, p < .001, RMSEA = .135[.127, .140]

Two factors: $\Delta\chi^2$ (19) = 779.4, p < .001, χ^2 (151) = 581.7, p < .001, RMSEA = .086[.078, .092]

Study 2

One reversed resistance to change item loaded strongest on the preference for equality factor, and there were a few cross-loadings (80% of the items performed completely as predicted).

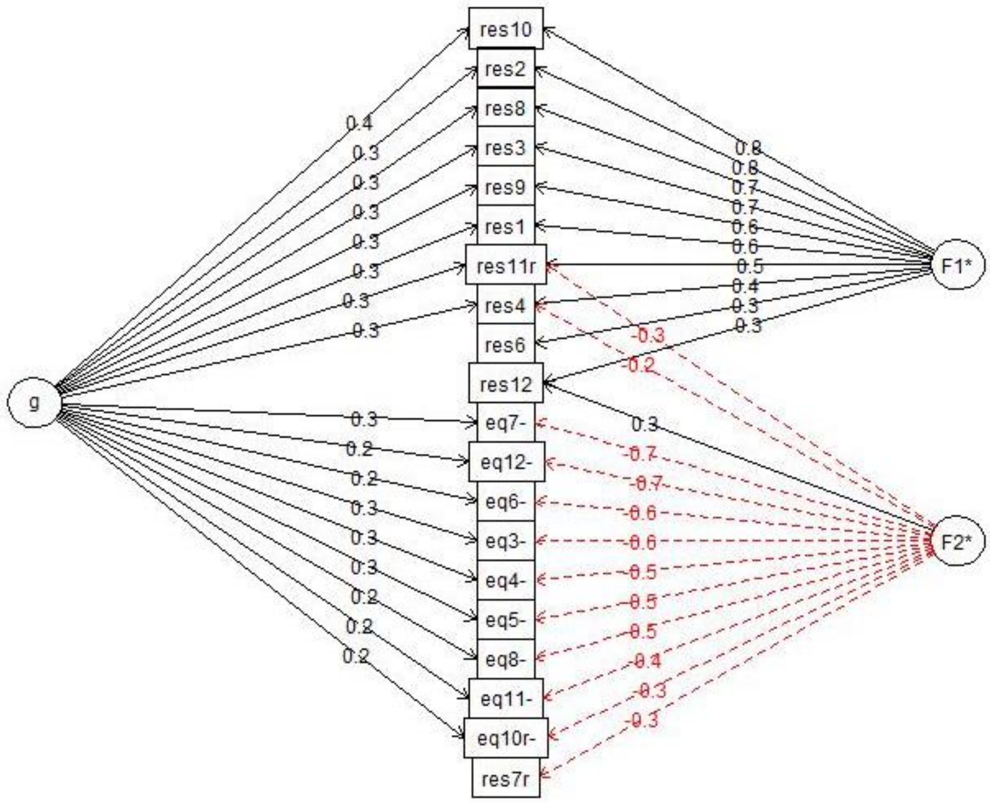

One general factor: χ^2 (170) = 1918.0, *p* < .001, RMSEA = .173[.164, .178]

Two factors: $\Delta\chi^2$ (19) = 1537.3, *p* < .001, χ^2 (151) = 380.7, *p* < .001, RMSEA = .067[.058, .074]

Study 4

Once again, one reversed resistance to change item loaded on the preference for equality factor, and two items loaded only on the general factor (82% of the items performed completely as predicted).

One general factor: χ^2 (170) = 588.1, *p* < .001, RMSEA = .111[.100, .118]

Three factors: $\Delta\chi^2$ (19) = 342.4, *p* < .001, χ^2 (151) = 245.7, *p* < .001, RMSEA = .066[.054, .075]

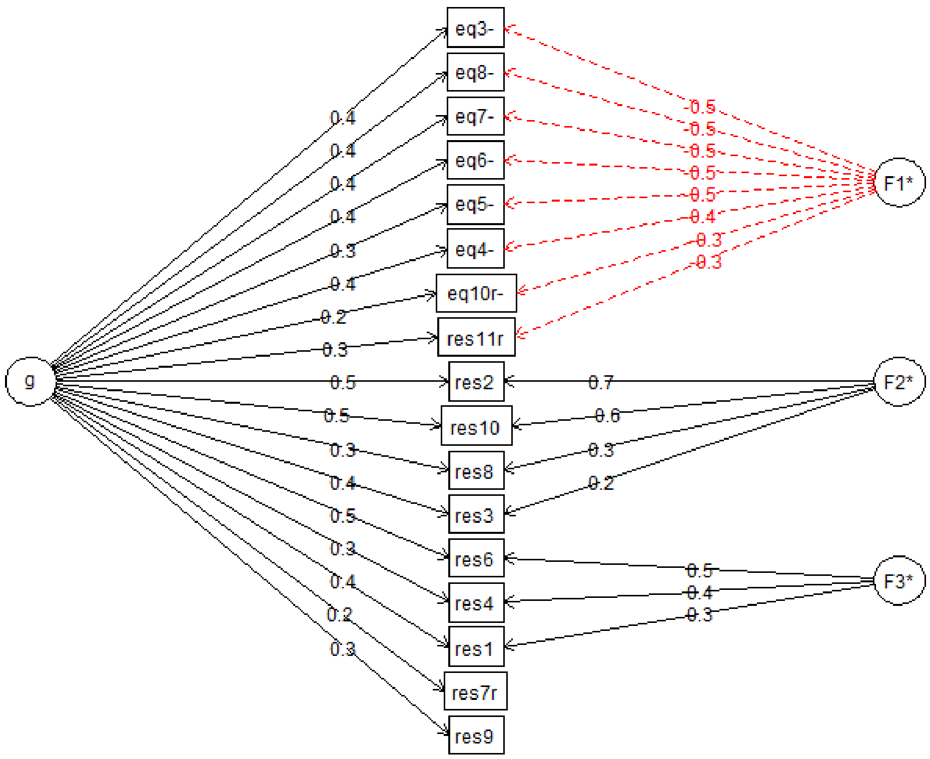

Normativism and resistance to change

Study 1

The two-factor structure was very clean in this case, but the two reversed resistance to change items did not load on any factor (92% of the items performed as predicted)

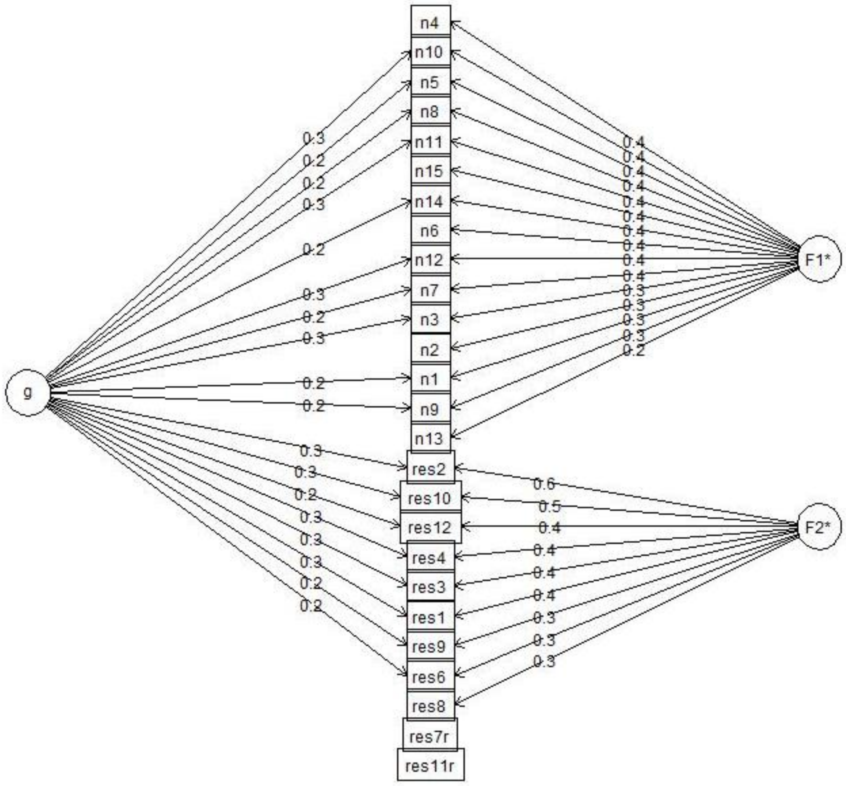

One general factor: χ^2 (299) = 1254.3, p < .001, RMSEA = .092[.085, .095]

Two factors: $\Delta\chi^2$ (25) = 451.4, p < .001, χ^2 (274) = 802.9, p < .001, RMSEA = .071[.064, .076]

Study 2

Three normativism items loaded on the resistance to change factor, two items loaded on both factors, and two items did not load on any of the factors (73% of the items performed completely as predicted).

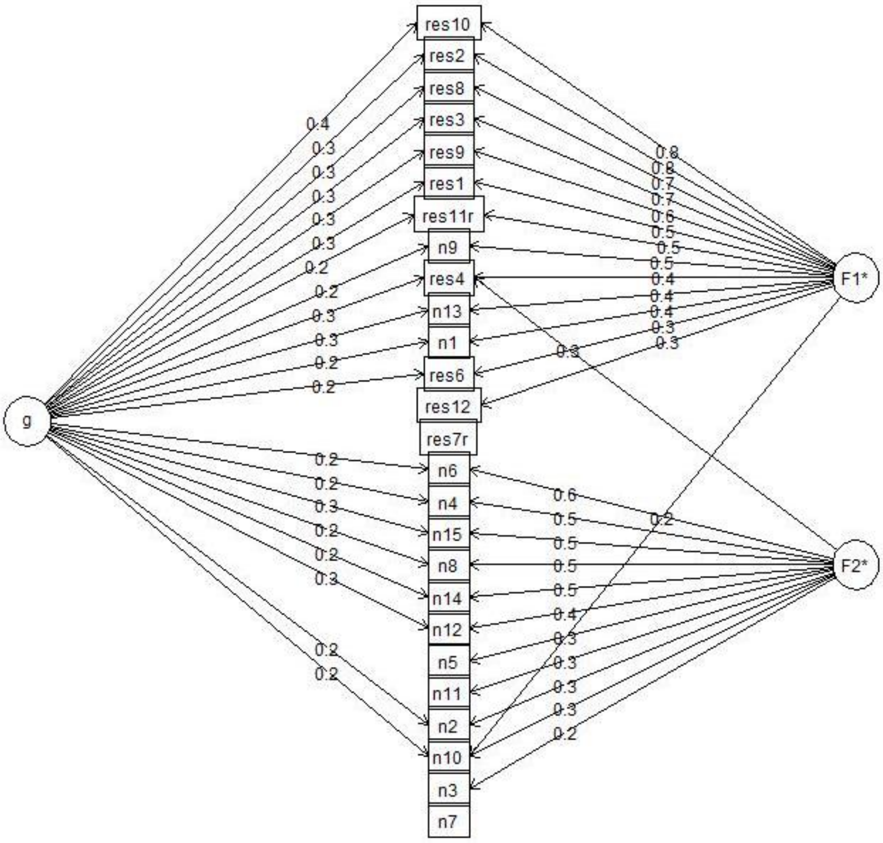

One general factor: χ^2 (299) = 1946.9, p < .001, RMSEA = .129 [.121, .132]

Two factors: $\Delta\chi^2$ (25) = 1168.7, p < .001, χ^2 (274) = 778.2, p < .001, RMSEA = .074 [.066, .078]

Study 4

Two resistance to change items loaded only on the normativism factor and two loaded on both factors, and three items did not load on any factor (67% of the items performed completely as predicted).

One general factor: χ^2 (252) = 1047.8, p < .001, RMSEA = .100[.091, .104]

Two factors: $\Delta\chi^2$ (23) = 363.2, p < .001, χ^2 (229) = 684.6, p < .001, RMSEA = .079[.071, .084]

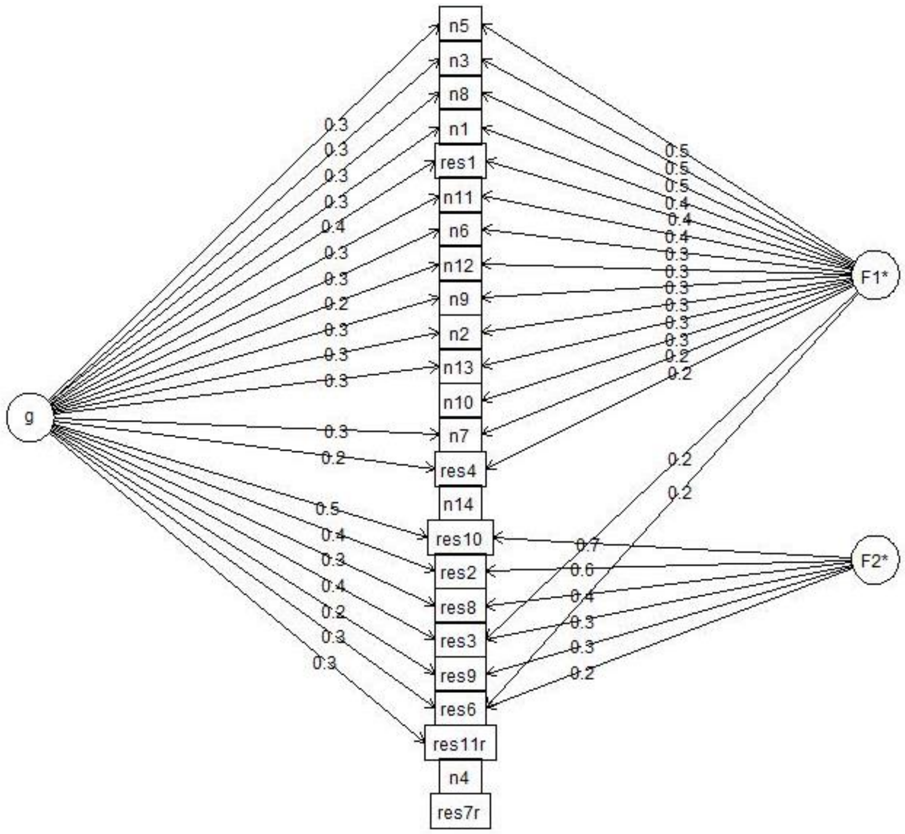

Humanism and preference for equality

Study 1

One humanism item loaded only on the preference for equality factor and one loaded on both factors (92% of the items performed completely as predicted).

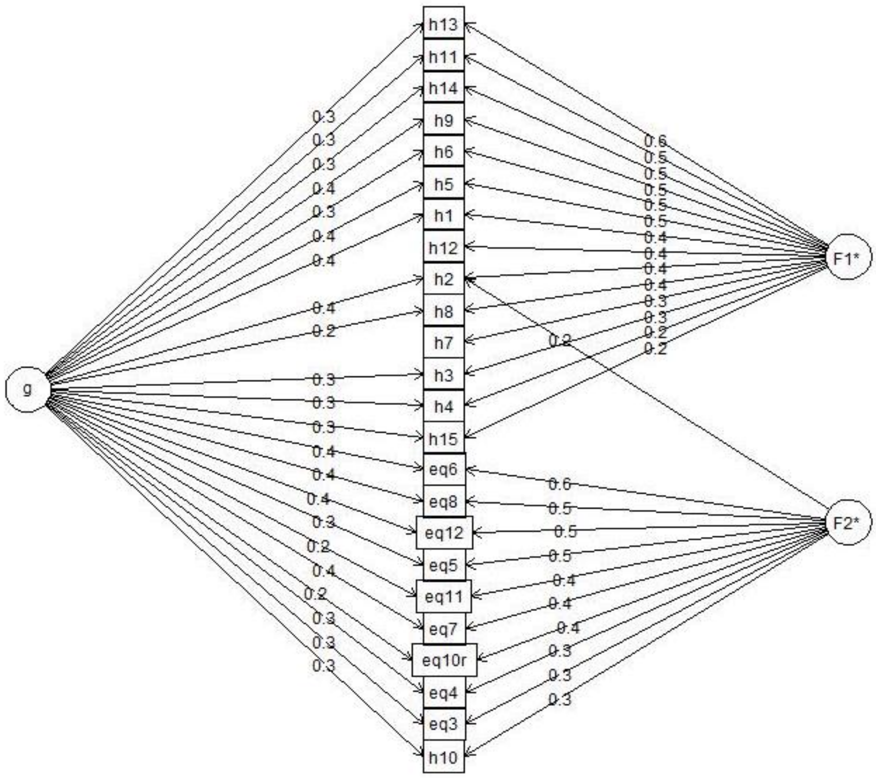

One general factor: χ^2 (252) = 1387.1, *p* < .001, RMSEA = .109[.101, .112]

Two factors: $\Delta\chi^2$ (23) = 612.6, *p* < .001, χ^2 (229) = 774.5, *p* < .001, RMSEA = .079[.072, .084]

Study 2

The scales overlapped more in this sample than in the others (50% of the items performed completely as predicted).

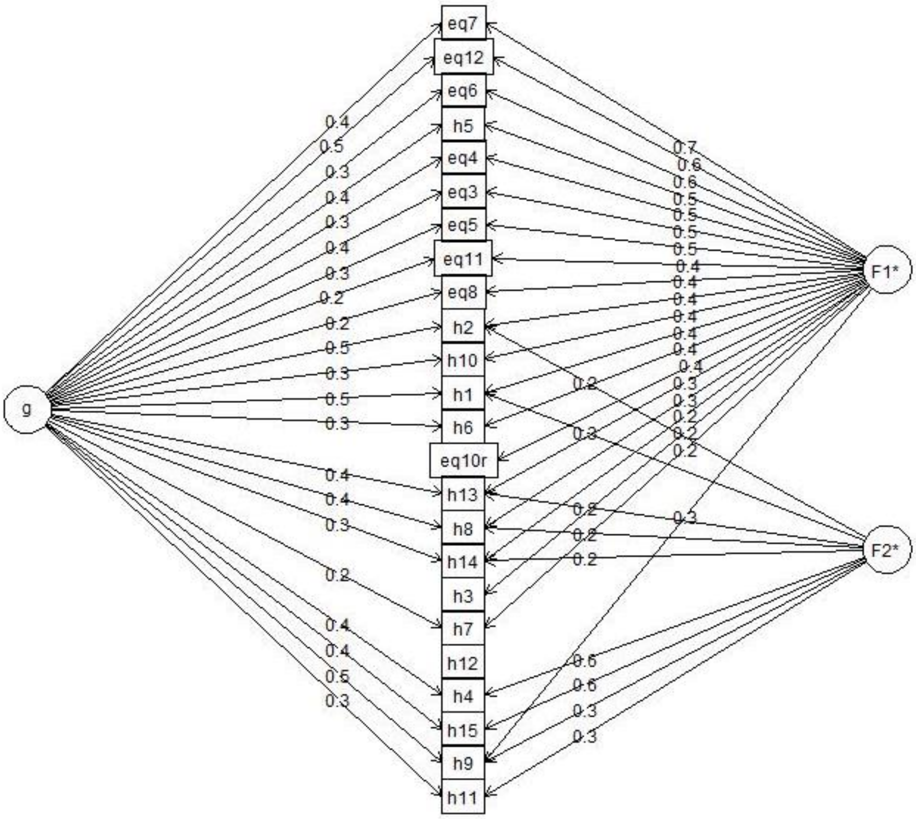

One general factor: χ^2 (252) = 1496.3, *p* < .001, RMSEA = .121[.113, .124]

Two factors: $\Delta\chi^2$ (23) = 667.6, *p* < .001, χ^2 (229) = 828.7, *p* < .001, RMSEA = .088[.080, .093]

Study 4

Four humanism items loaded only on the preference for equality factor, three loaded only on the general factor, and one did not load on any factor (59% of the items performed completely as predicted).

One general factor: χ^2 (209) = 698.0, *p* < .001, RMSEA = .086[.077, .091]

Two factors: $\Delta\chi^2$ (21) = 451.4, *p* < .001, χ^2 (188) = 423.4, *p* < .001, RMSEA = .063[.054, .069]

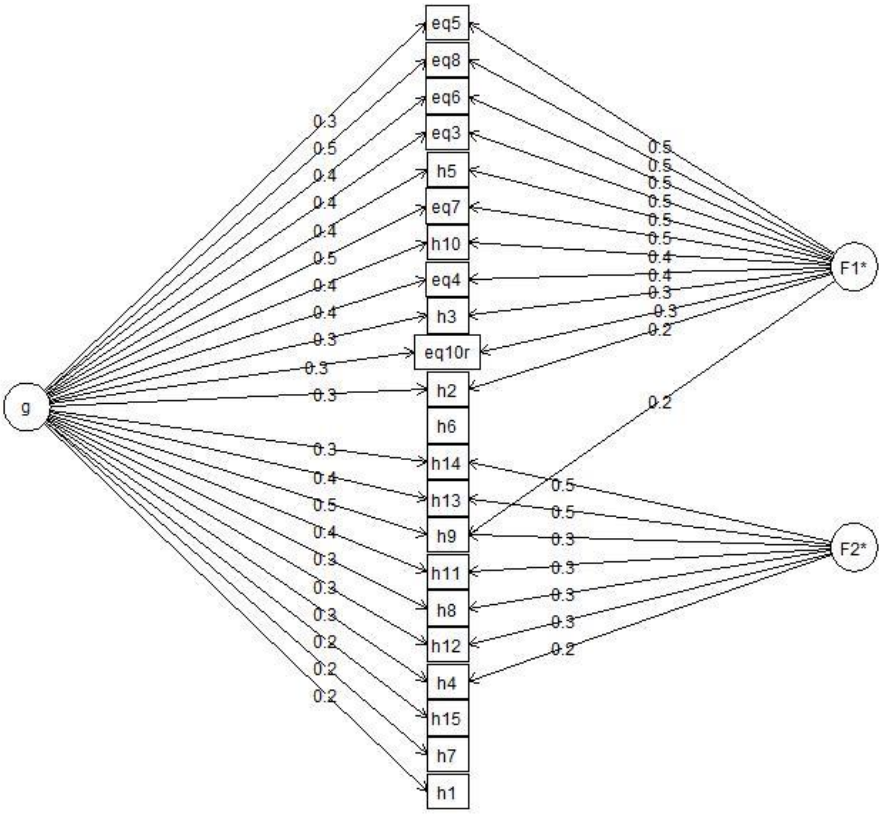

Right-wing authoritarianism and social dominance orientation

Study 1

Three reversed RWA items loaded on the SDO factor, one did not load on any factor, and there was one cross-loading (78% of the items performed completely as predicted).

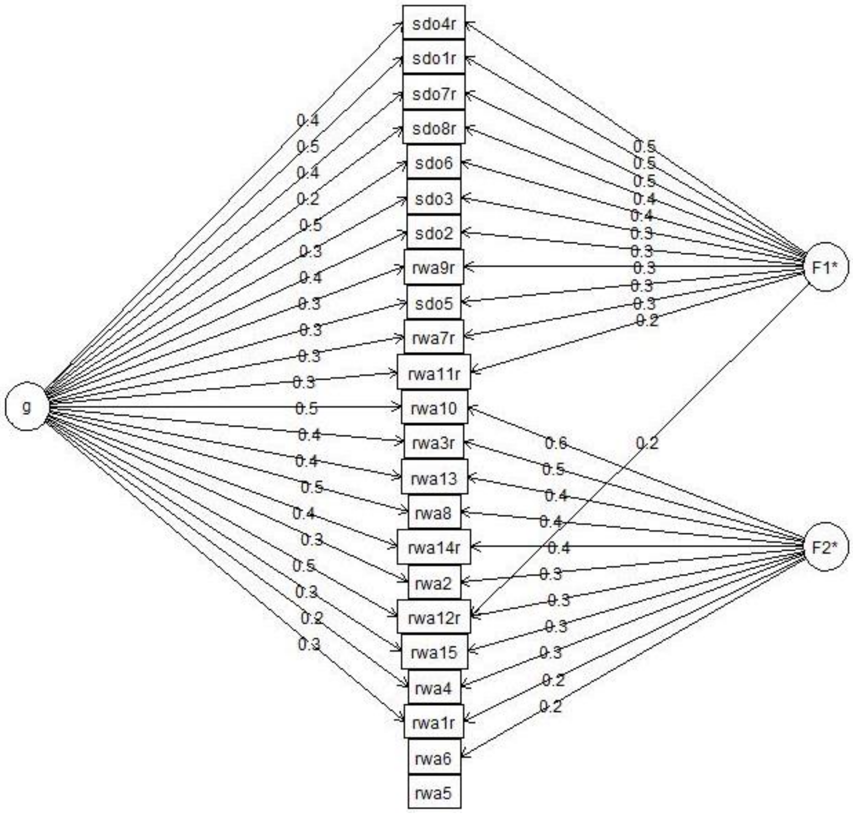

One general factor: χ^2 (230) = 1143.0 *p* < .001, RMSEA = .103[.096, .108]

Two factors: $\Delta\chi^2$ (22) = 427.3, *p* < .001, χ^2 (208) = 715.7, *p* < .001, RMSEA = .081[.073, .086]

Study 2

Although all items loaded on the factor they were supposed to load on, there were seven cross-loadings (70% of the items performed completely as predicted).

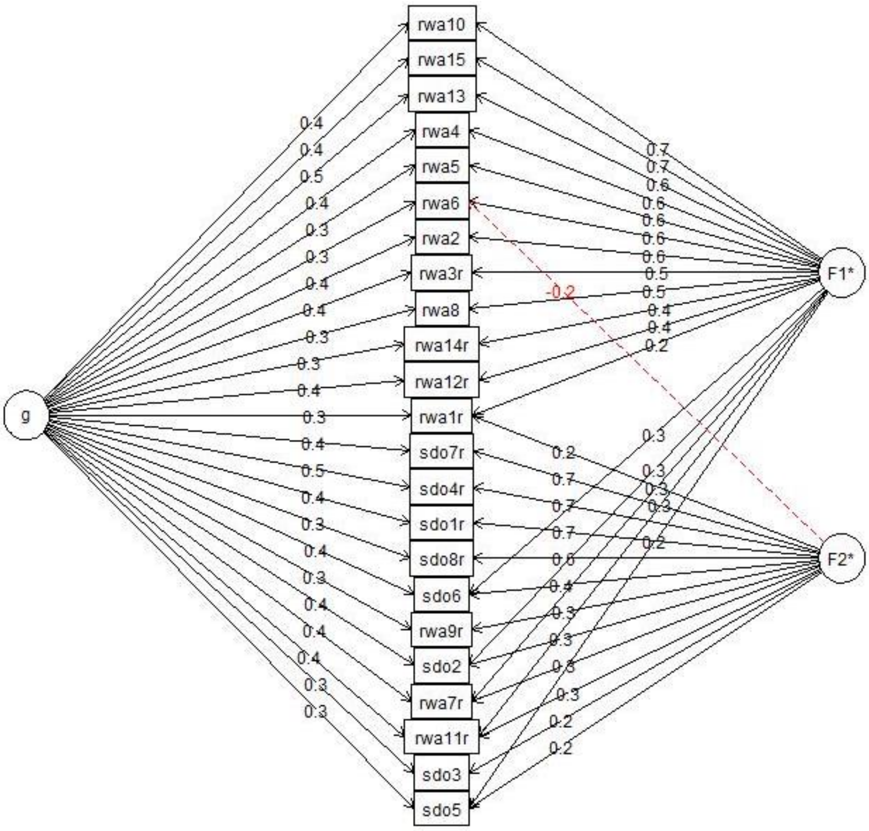

One general factor: χ^2 (230) = 2719.6, *p* < .001, RMSEA = .179 [.170, .182]

Two factors: $\Delta\chi^2$ (22) = 1323.2, *p* < .001, χ^2 (208) = 1396.4, *p* < .001, RMSEA = .130[.122, .134]

Study 3

Three reversed RWA items loaded on the SDO factor and two SDO items loaded on the RWA factor (65% of the items performed completely as predicted).

One general factor: χ^2 (230) = 1307.5, *p* < .001, RMSEA = .116[.108, .120]

Two factors: $\Delta\chi^2$ (22) = 623.4, *p* < .001, χ^2 (208) = 684.1, *p* < .001, RMSEA = .081[.073, .086]

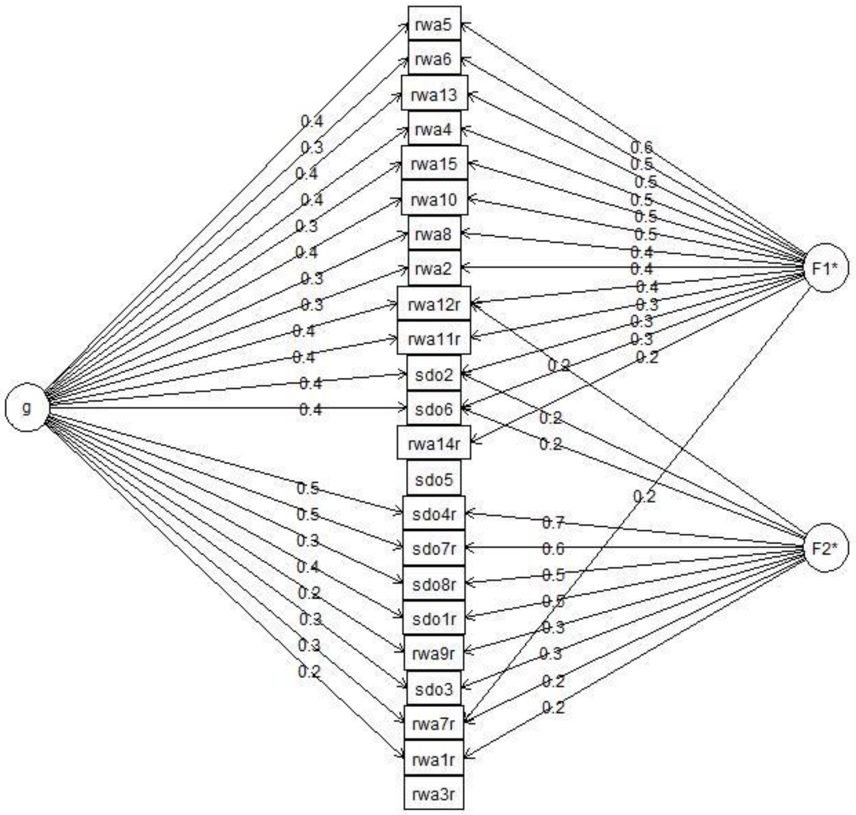

Right-wing authoritarianism and dangerous-world beliefs

Study 1

Four RWA items loaded on the DWB-factor, two did not load on any factor, and one DWB-item loaded on the RWA factor (72% of the items performed as predicted).

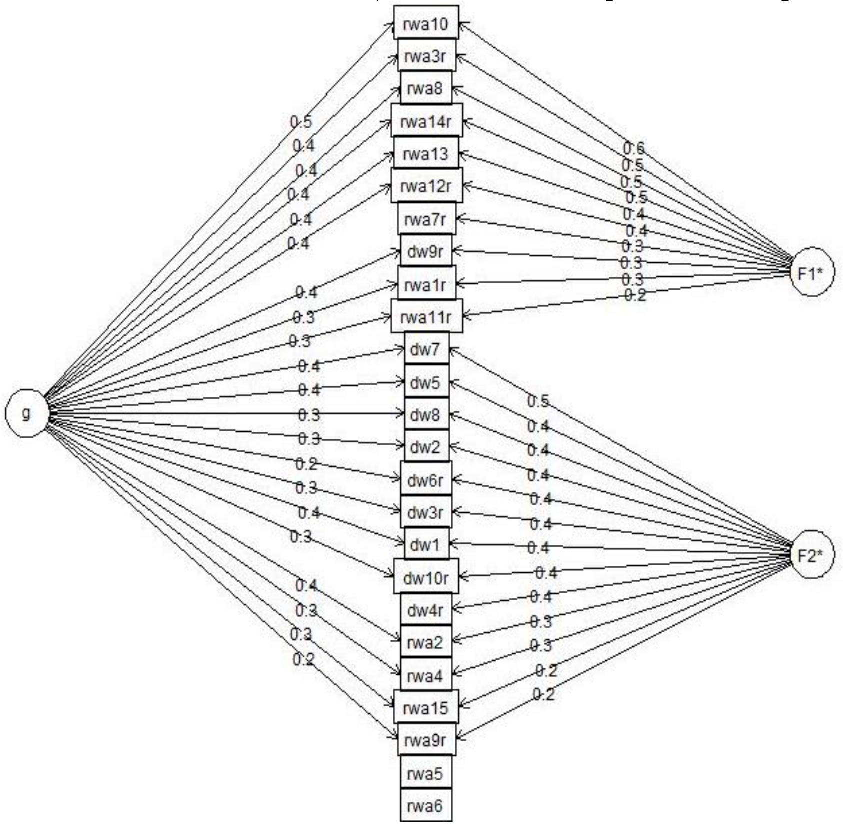

One general factor: χ^2 (275) = 1217.6, *p* < .001, RMSEA = .096[.089, .100]

Two factors: $\Delta\chi^2$ (24) = 524.0, *p* < .001, χ^2 (251) = 693.6, *p* < .001, RMSEA = .069[.062, .074]

Study 2

Three reversed RWA items loaded on the DWB-factor, one RWA-item loaded only on the general factor, and there were seven cross-loadings (56% of the items performed completely as predicted).

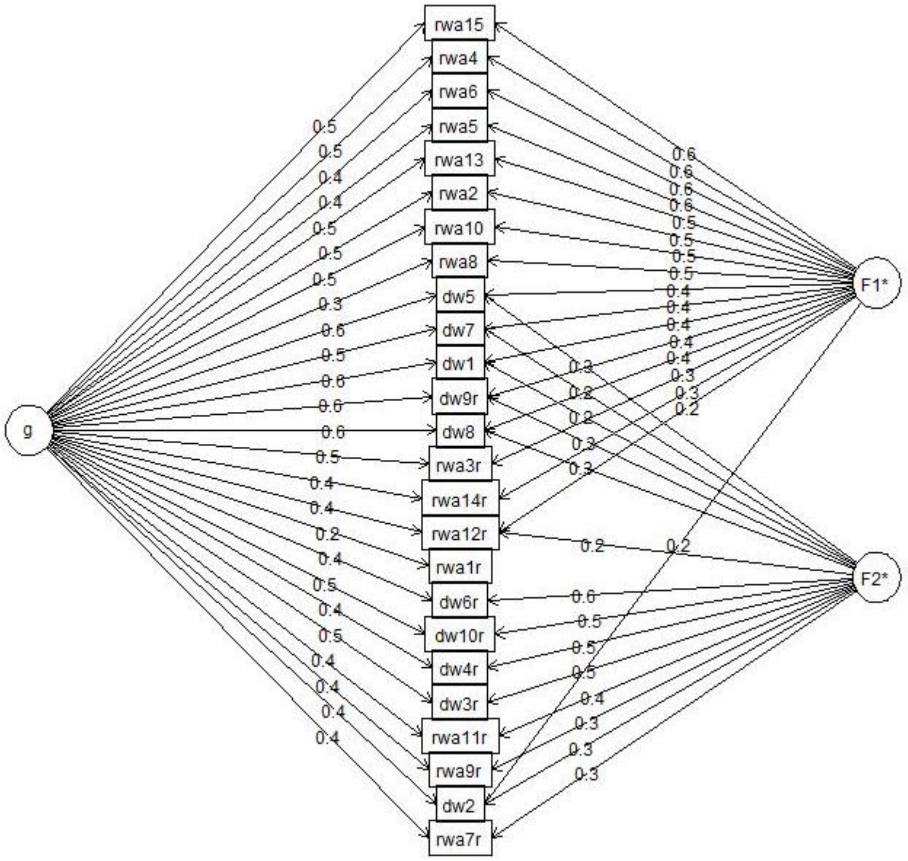

One general factor: χ^2 (275) = 2228.6, *p* < .001, RMSEA = .145[.137, .148]

Two factors: $\Delta\chi^2$ (24) = 896.6, *p* < .001, χ^2 (251) = 1332.0, *p* < .001, RMSEA = .113[.105, .117]

Study 3

The largest factor was a mix of both constructs (56% of the items performed completely as predicted).

One general factor: χ^2 (275) = 1044.0, *p* < .001, RMSEA = .090[.082, .094]

Three factors: $\Delta\chi^2$ (47) = 505.7, *p* < .001, χ^2 (228) = 538.3, *p* < .001, RMSEA = .063[.055, .068]

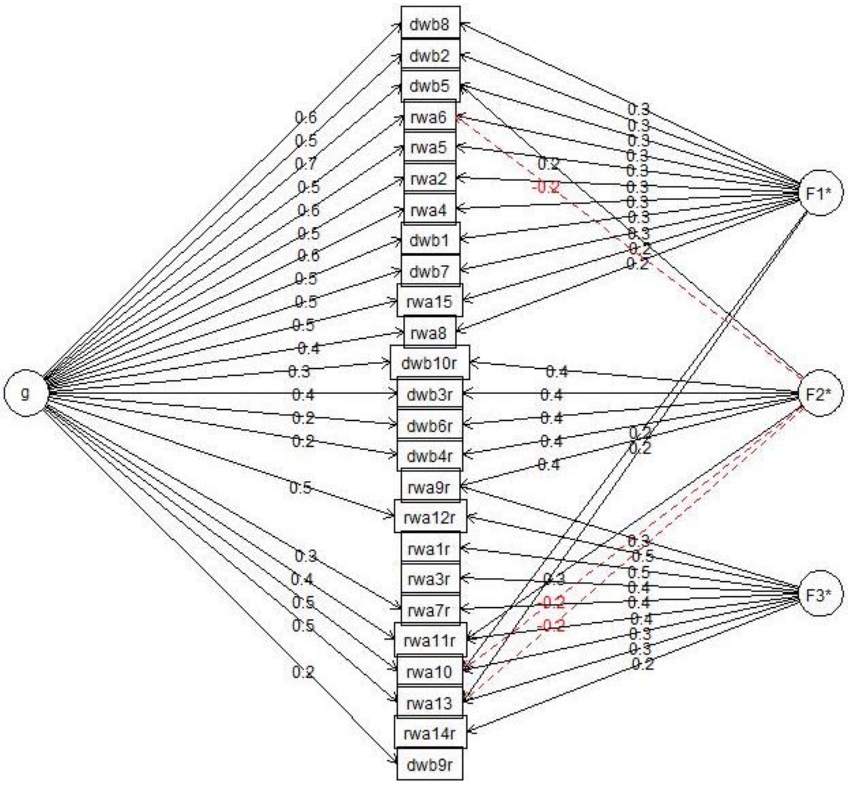

Social dominance orientation and competitive-world beliefs

Study 1

One SDO-item loaded on the CWB factor, one CWB-item loaded on the SDO factor, and four additional items cross-loaded (76% of the items performed as predicted).

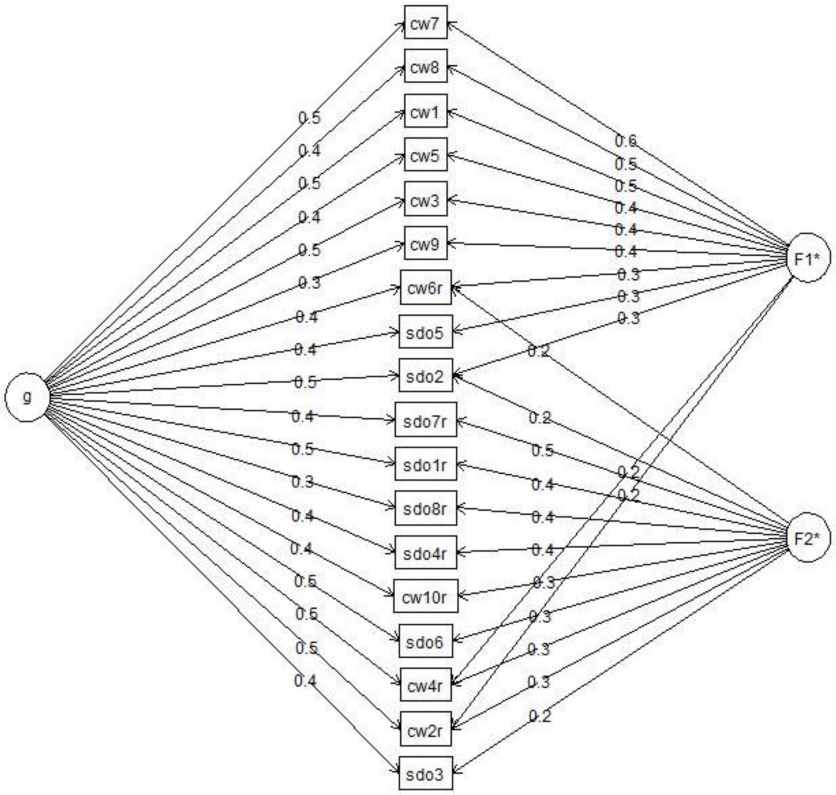

One general factor: χ^2 (135) = 2278.8, p < .001, RMSEA = .147[.141, .151]

Two factors: $\Delta\chi^2$ (17) = 1454.5, p < .001, χ^2 (118) = 824.3, p < .001, RMSEA = .090[.084, .095]

Study 2

There was a relatively high degree of overlap between the scales (61% of the items performed completely as predicted).

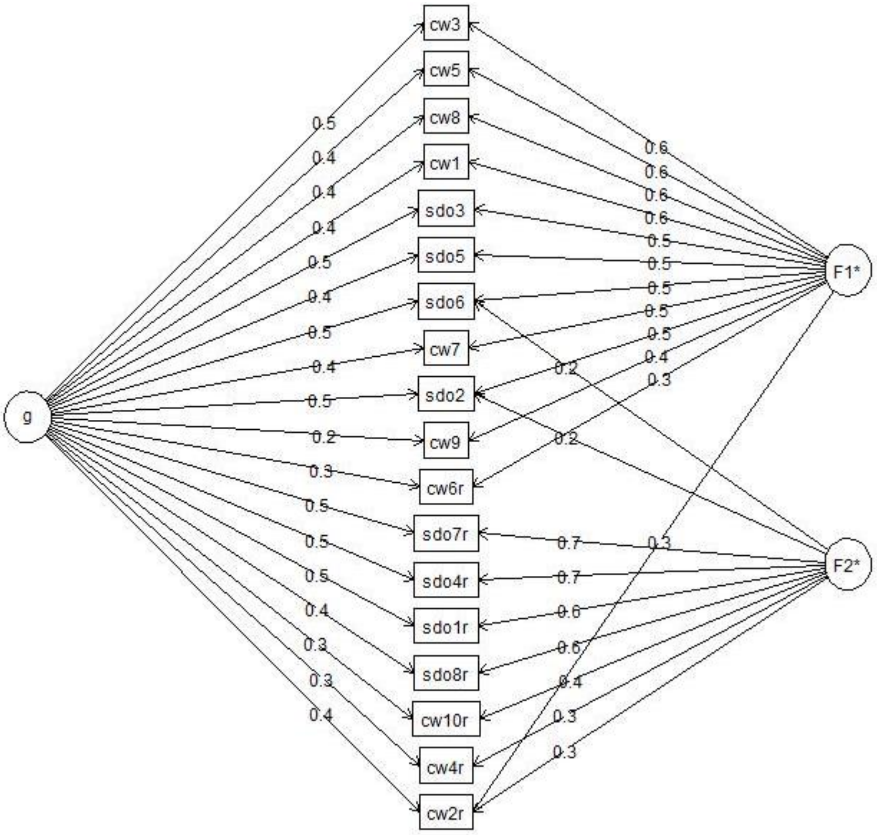

One general factor: χ^2 (135) = 1616.4, p < .001, RMSEA = .180[.170, .185]

Two factors: $\Delta\chi^2$ (17) = 1162.9, p < .001, χ^2 (118) = 453.5, p < .001, RMSEA = .092[.082, .099]

Study 3

Once again, there was a relatively high degree of overlap between the scales (56% of the items performed completely as predicted).

One general factor: χ^2 (135) = 850.4, p < .001, RMSEA = .123 [.114, .129]

Two factors: $\Delta\chi^2$ (17) = 523.5, p < .001, χ^2 (118) = 326.9, p < .001, RMSEA = .071 [.061, .079]

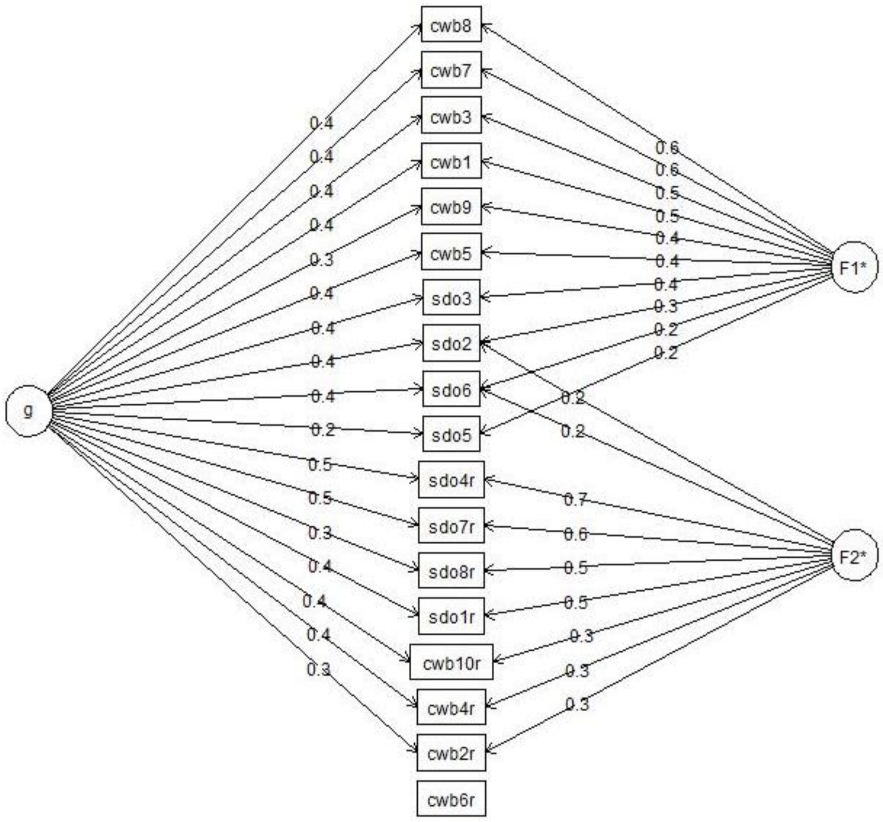

Normativism and right-wing authoritarianism

Study 1

Two RWA-items loaded on the normativism factor, two cross-loaded, and two did not load on any factor (80% of all the items performed as predicted).

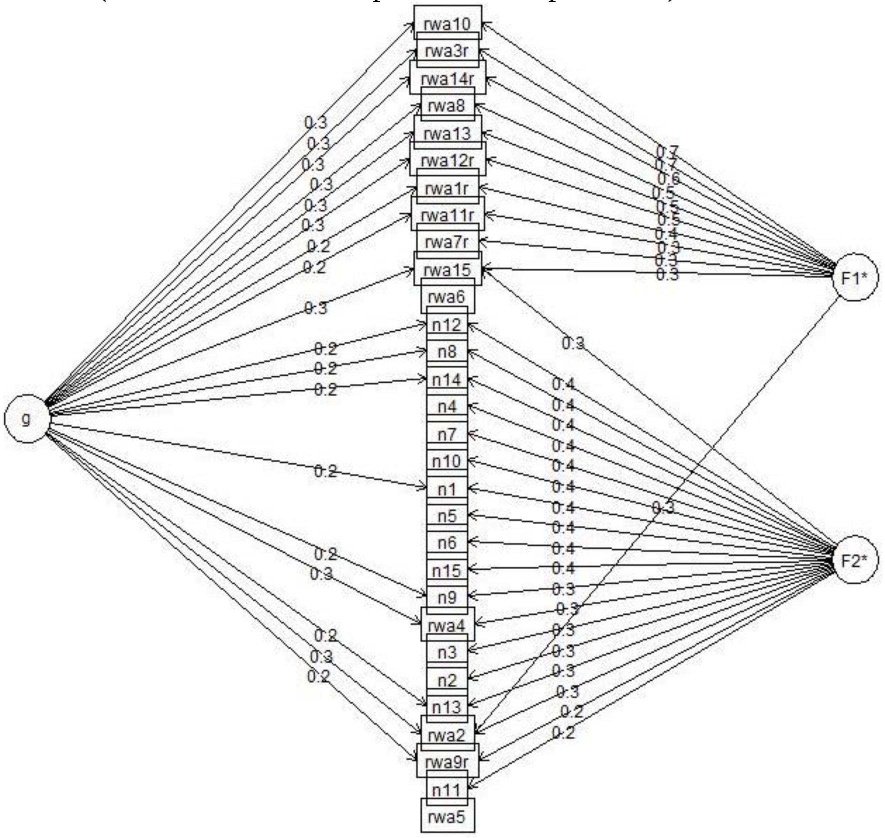

One general factor: χ^2 (405) = 1792.2, *p* < .001, RMSEA = .096[.090, .099]

Two factors: $\Delta\chi^2$ (29) = 756.4, *p* < .001, χ^2 (376) = 1035.8, *p* < .001, RMSEA = .069[.063, .073]

Study 2

Five RWA-items loaded on the normativism factor, one normativism item loaded on the RWA-factor, and eight items cross-loaded (53% of the items performed as predicted).

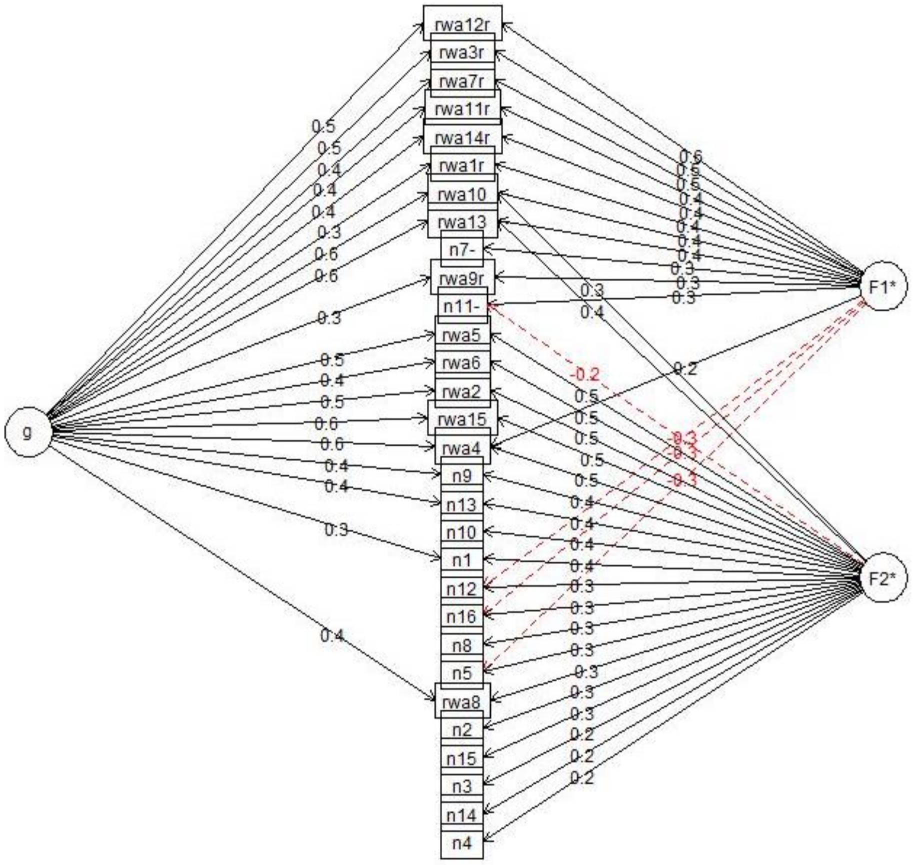

One general factor: χ^2 (405) = 2232.9, *p* < .001, RMSEA = .116[.109, .118]

Two factors: $\Delta\chi^2$ (29) = 879.4, *p* < .001, χ^2 (376) = 1353.5, *p* < .001, RMSEA = .089[.081, .091]

Study 3

One item loaded on both factors and two did not load on any factor (90% of the items performed completely as predicted).

One general factor: χ^2 (405) = 1517.5, *p* < .001, RMSEA = .089 [.083, .092]

Two factors: $\Delta\chi^2$ (29) = 426.7, *p* < .001, χ^2 (376) = 1090.8, *p* < .001, RMSEA = .075 [.068, .078]

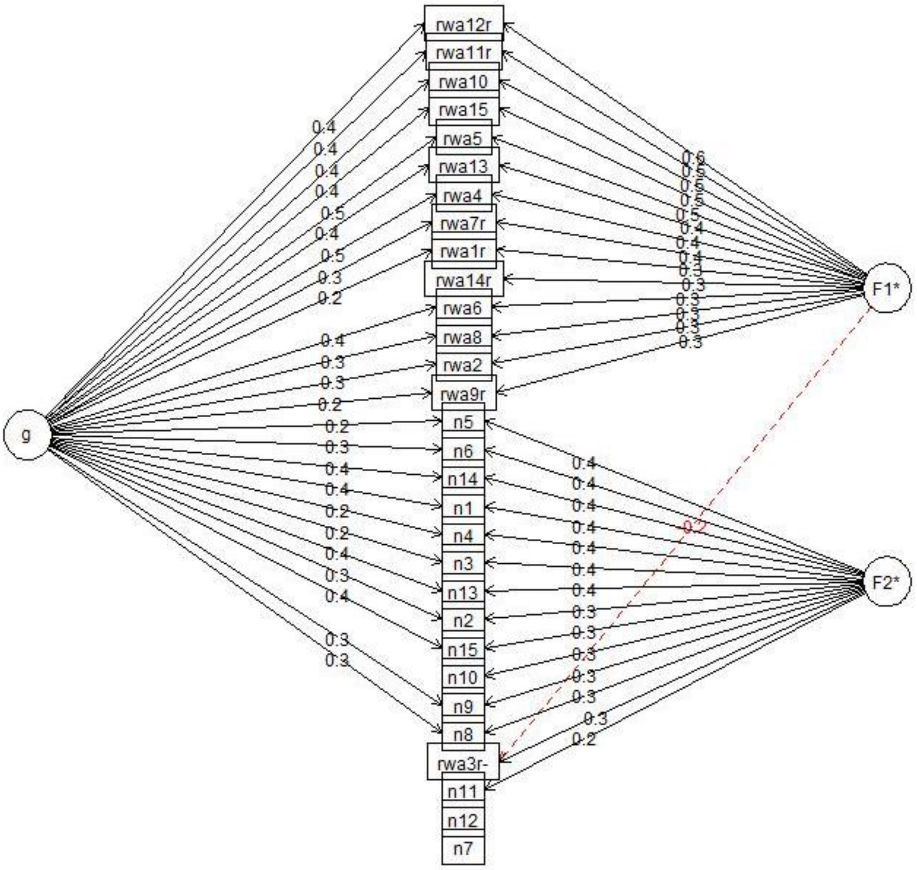

Humanism and social dominance orientation

Study 1

Two humanism items loaded on the SDO factor and two loaded only on the general factor (83% of the items performed completely as predicted).

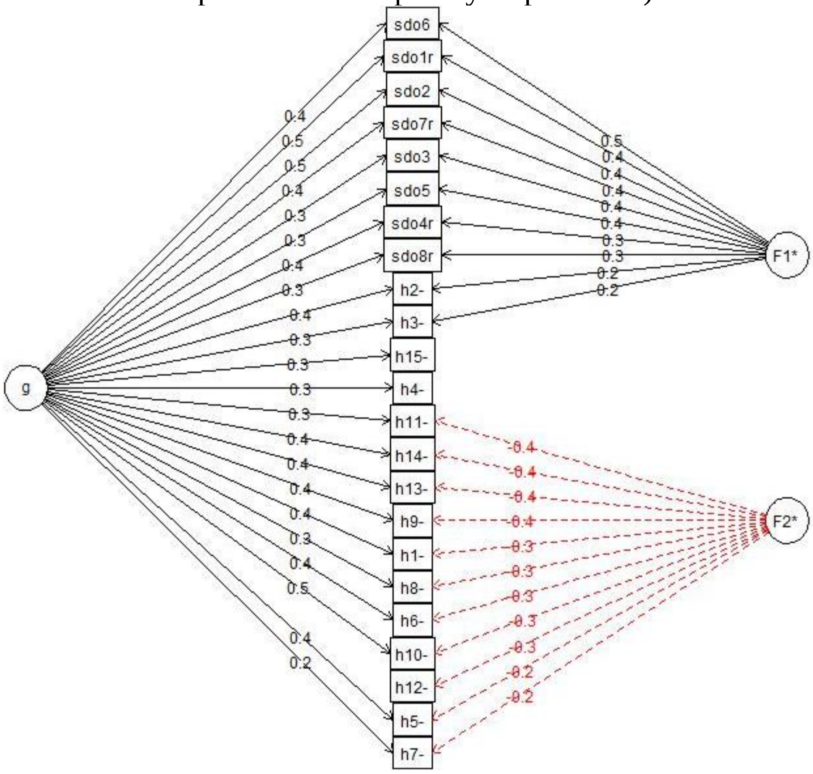

One general factor: χ^2 (230) = 766.9, p < .001, RMSEA = .079[.072, .084]

Two factors: $\Delta\chi^2$ (22) = 241.3, p < .001, χ^2 (208) = 502.8, p < .001, RMSEA = .062[.054, .068]

Study 2

One humanism item failed to load on any factor and the reversed SDO items loaded either only on the humanism factor or on both factors (72% of the items performed as predicted).

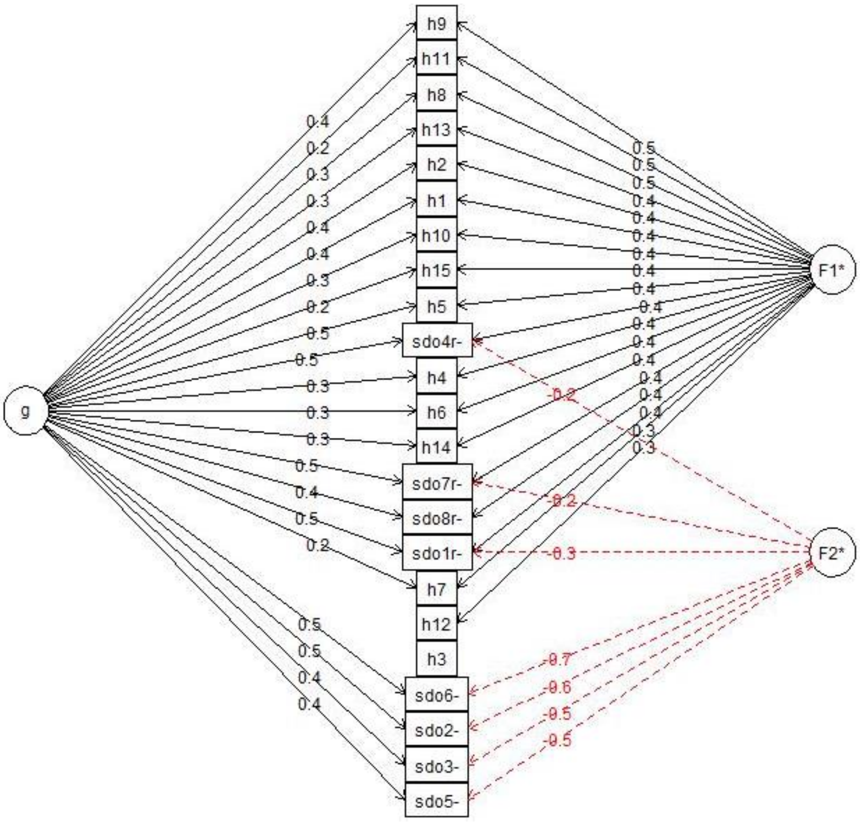

One general factor: χ^2 (230) = 1837.4, p < .001, RMSEA = .144[.136, .148]

Two factors: $\Delta\chi^2$ (22) = 761.1, p < .001, χ^2 (208) = 1076.3, p < .001, RMSEA = .111[.103, .116]

Study 3

Once again, one humanism item loaded on the SDO factor, one loaded on both factors, and one did not load on any factor (87% of the items performed completely as predicted).

One general factor: χ^2 (230) = 1722.3, p < .001, RMSEA = .136 [.128, .140]

Two factors: $\Delta\chi^2$ (22) = 1022.7, p < .001, χ^2 (208) = 699.6, p < .001, RMSEA = .083 [.074, .088]

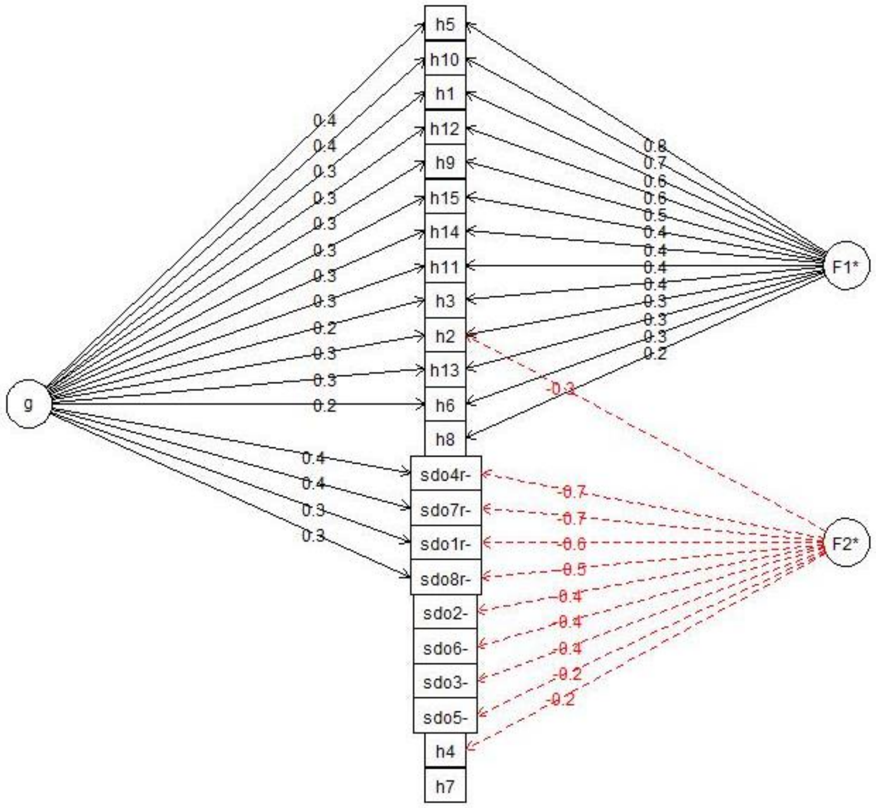

Normativism and dangerous-world belief

Study 1

One normativism item did not load on any factor and four DWB-items cross-loaded (80% of the items performed completely as predicted).

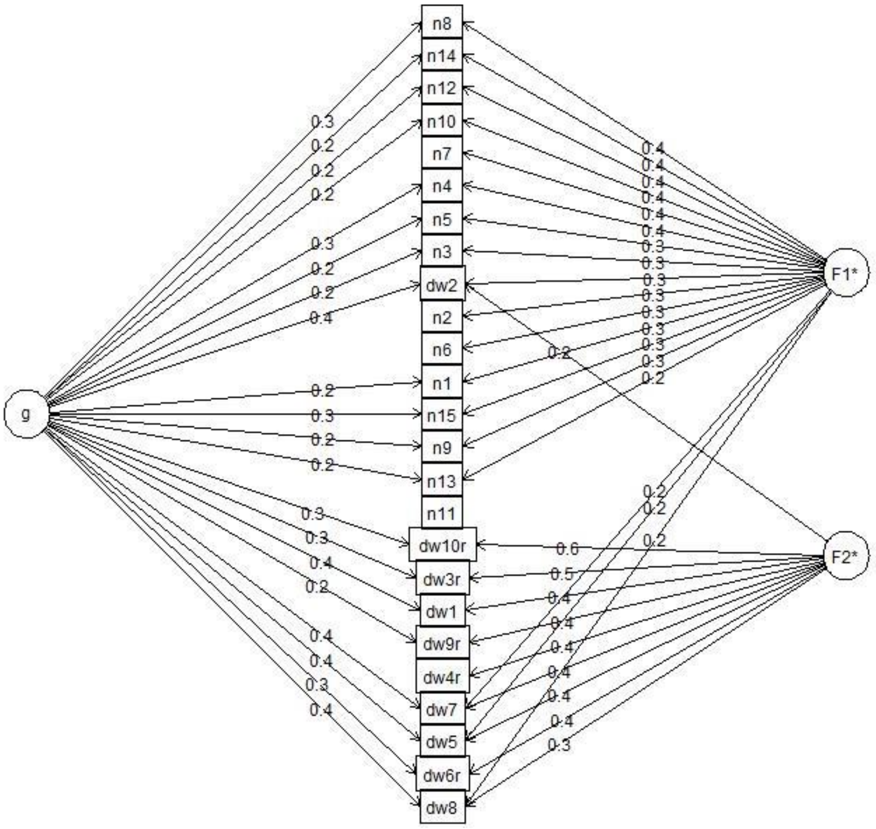

One general factor: χ^2 (275) = 1096.2, p < .001, RMSEA = .090[.083, .094]

Two factors: $\Delta\chi^2$ (24) = 438.6, p < .001, χ^2 (251) = 657.6, p < .001, RMSEA = .066[.059, .071]

Study 2

One normativism item did not load on any factor, two loaded on the DWB-factor, and one cross-loaded, while three DWB-items cross-loaded (72% of the items performed completely as predicted).

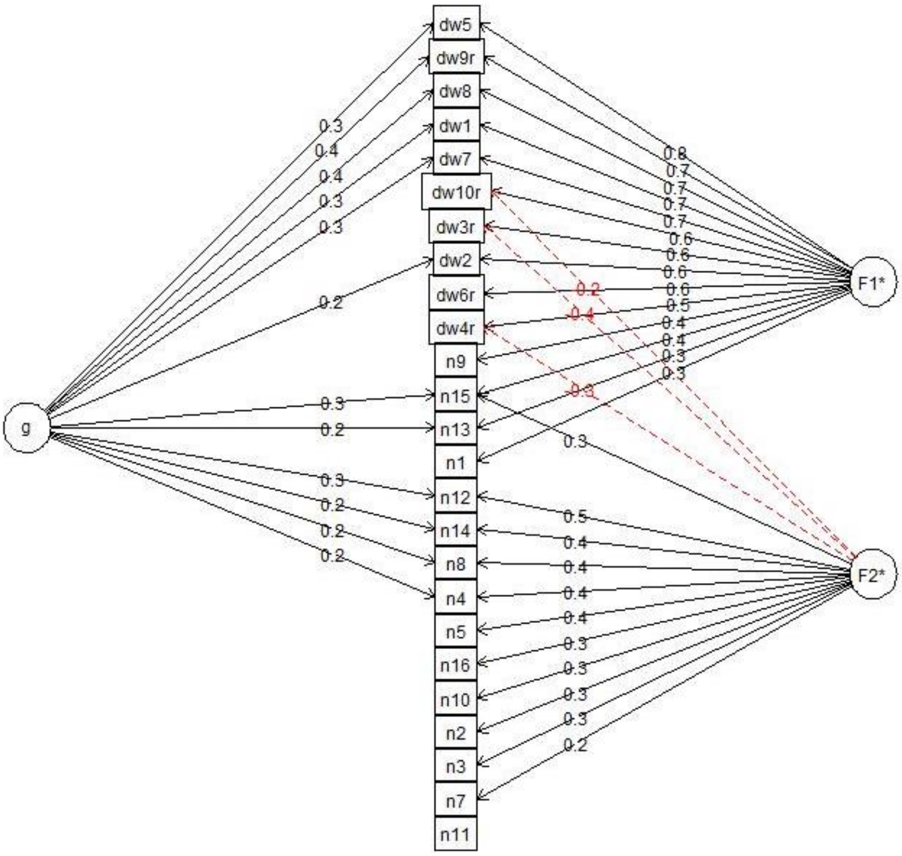

One general factor: χ^2 (275) = 2282.1, p < .001, RMSEA = .147[.139, .150]

Two factors: $\Delta\chi^2$ (24) = 1361.2, p < .001, χ^2 (251) = 920.9, p < .001, RMSEA = .089[.081, .094]

Study 3

One general factor: χ^2 (275) = 1227.5, p < .001, RMSEA = .100 [.093, .104]

Three factors: $\Delta\chi^2$ (47) = 551.8, p < .001, χ^2 (228) = 675.7, p < .001, RMSEA = .076 [.067, .080]; α = .80, hierarchical ω = .51.

There were three item cross-loadings (88% of the items performed completely as predicted).

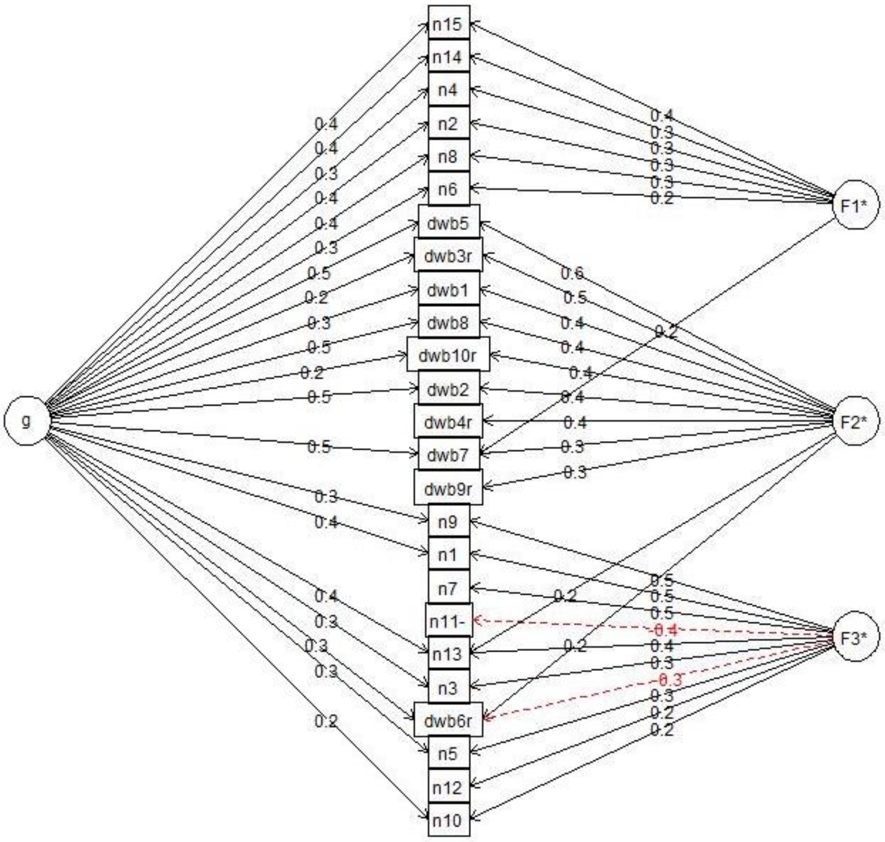

Humanism and competitive-world beliefs

Study 1

One CWB-item loaded on the humanism factor, one humanism item loaded on the CWB-factor, and one item cross-loaded (88% loaded completely as predicted).

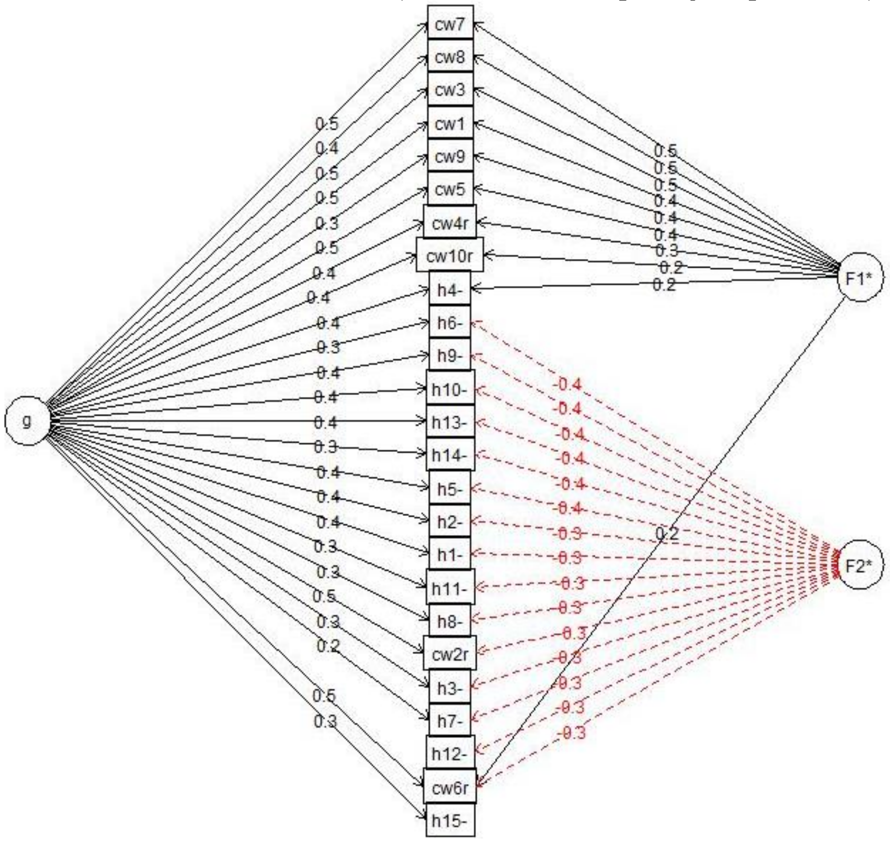

One general factor: χ^2 (275) = 974.9, *p* < .001, RMSEA = .083[.076, .087]

Two factors: $\Delta\chi^2$ (24) = 414.7, *p* < .001, χ^2 (251) = 560.2, *p* < .001, RMSEA = .058[.050, .063]

Study 2

One humanism items did not load on any factor, two reversed CWB-items loaded on the humanism factor, and two CWB-items cross-loaded (80% of the items performed completely as predicted).

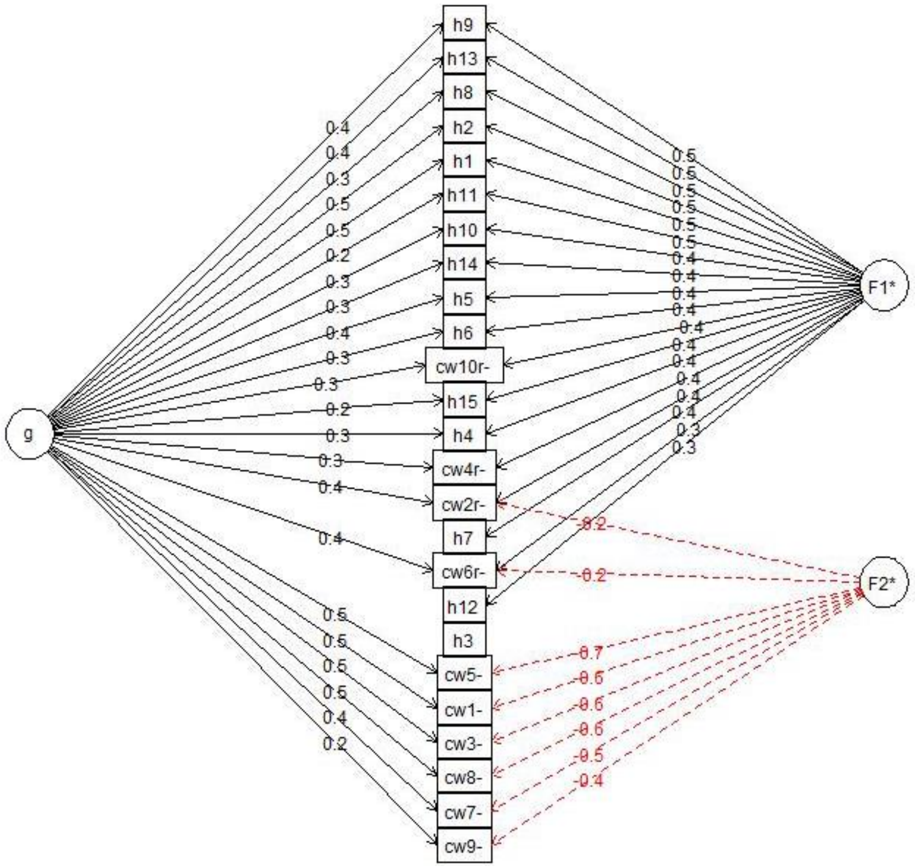

One general factor: χ^2 (275) = 1783.1, *p* < .001, RMSEA = .128[.120, .131]

Two factors: $\Delta\chi^2$ (24) = 907.3, *p* < .001, χ^2 (251) = 875.8, *p* < .001, RMSEA = .086[.078, .091]

Study 3

Two humanism items loaded on the competitive-world beliefs factor, one loaded on both factors, and one did not load on any factor (84% of the items performed completely as predicted).

One general factor: χ^2 (275) = 2489.2, *p* < .001, RMSEA = .152 [.144, .155]

Three factors: $\Delta\chi^2$ (24) = 1784.4, *p* < .001, χ^2 (251) = 704.8, *p* < .001, RMSEA = .073 [.065, .077]

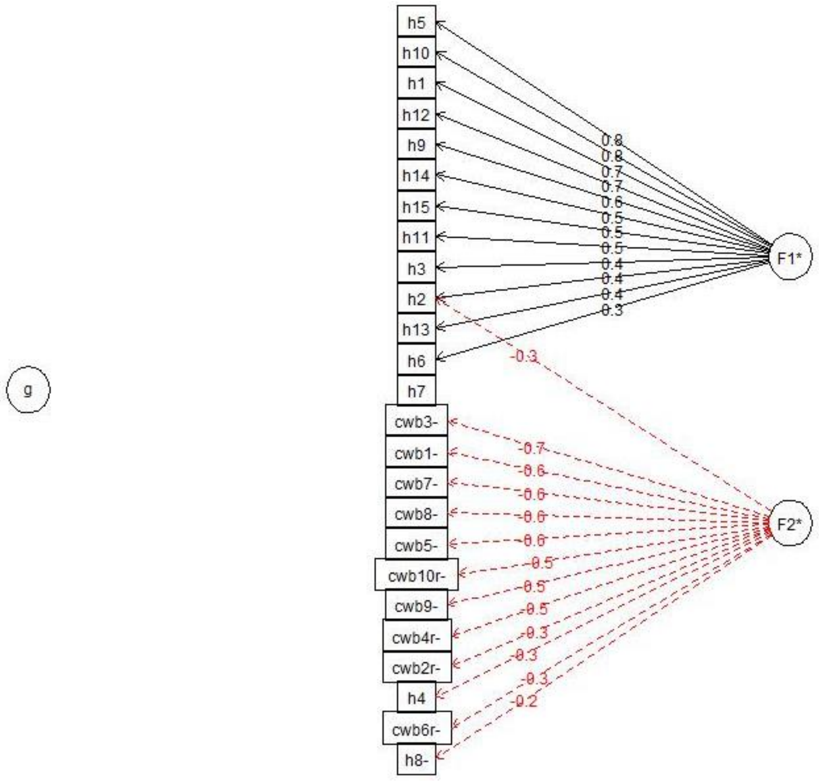
h5
h10
h1
h12
h9
h14
h15
h11
h3
h2
h13
h6
h7
cwb3-
cwb1-
cwb7-
cwb8-
cwb5-
cwb10r-
cwb9-
cwb4r-
cwb2r-
h4
cwb6r-
h8-
g
F1*
F2*
0.8
0.8
0.7
0.7
0.6
0.5
0.5
0.5
0.4
0.4
0.4
0.3
-0.3
-0.7
-0.6
-0.6
-0.6
-0.6
-0.5
-0.5
-0.5
-0.3
-0.3
-0.3
-0.2
