# Supplementary material for: Rediscovering Tomkins’ polarity theory: Humanism, normativism, and the psychological basis of left-right ideological conflict in the U.S. and Sweden
Source: PLoS One. 2020 Jul 31;15(7):e0236627. doi: 10.1371/journal.pone.0236627 (PMC7394437; doi:10.1371/journal.pone.0236627)
Supplement: S3 Appendix — This document reports results of analyses of indirect paths from humanism and normativism to political orientation. (PDF) [file pone.0236627.s003.pdf]

Indirect relations

Indirect relations based on all structural equation models we tested (calculated in AMOS 25.0) are reported in the supplementary tables below. We report unstandardized indirect relations with 95% bias-corrected bootstrap confidence intervals (20000 resamples) and *p*-values. We report total indirect relations (i.e., the sum of all paths that run through a given mediator from predictor to outcome).

Supplementary Table 1. Indirect relations based on the model of ideology as motivated social cognition.

	Unstandardized indirect effect		
	Study 1	Study 2	Study 4
HUM → SJ → PFE	.034[-.014, .130]<br>$p = .16$	.032[-.006, .088]<br>$p = .090$	.034[-.040, .176]<br>$p = .36$
HUM → SJ → RES	-.021[-.120, .009]<br>$p = .19$	-.022[-.082, .003]<br>$p = .093$	-.034[-.172, .042]<br>$p = .37$
HUM → SJ → LR	-.019[-.109, .009]<br>$p = .18$	-.030[-.100, .006]<br>$p = .10$	-.084[-.334, .124]<br>$p = .45$
HUM → PFE → LR	-.128[-.318, -.001]<br>$p = .046$	-.175[-.397, .028]<br>$p = .085$	-.534[-1.19, -.218]<br>$p = .003$
HUM → RES → LR	-.071[-.222, .065]<br>$p = .28$	-.277[-.487, -.130]<br>$p < .001$	-.007[-.102, .030]<br>$p = .50$
NORM → SJ → PFE	-.044[-.159, .003]<br>$p = .068$	-.161[-.359, -.064]<br>$p < .001$	-.238[-.530, -.075]<br>$p = .002$
NORM → SJ → RES	.027[-.008, .135]<br>$p = .15$	.107[.010, .293]<br>$p = .029$	.243[.083, .466]<br>$p = .008$
NORM → SJ → LR	.025[-.007, .136]<br>$p = .15$	.151[.048, .364]<br>$p = .001$	.599[.356, .957]<br>$p < .001$
NORM → PFE → LR	.093[.006,.227]<br>$p = .036$	.111[-.004, .318]<br>$p = .057$	.249[.075, .559]<br>$p = .003$
NORM → RES → LR	.207[.073, .402]<br>$p = .001$	1.34[.936, 2.06]<br>$p < .001$	.124[-.126, .397]<br>$p = .23$

Note. HUM = Humanism; NORM = Normativism; SJ = General system justification; PFE = Preference for equality; RES = Resistance to change; LR = Left-right self-placement.

Supplementary Table 2. Indirect relations based on the dual-process model of ideology.

	Unstandardized indirect effect		
	Study 1	Study 2	Study 3
HUM → CWB → SDO	-.269[-.420, -.158]<br>$p < .001$	-.340[-.565, -.053]<br>$p = .026$	-.069[-.197, .009]<br>$p = .084$
HUM → DWB → SDO	-.009[-.024, .000]<br>$p = .051$	-.008[-.033, .005]<br>$p = .24$	-.002[-.023, .005]<br>$p = .46$
HUM → CWB → RWA	-.027[-.055, -.008]<br>$p = .006$	-.031[-.096, .050]<br>$p = .35$	-.006[-.032, .002]<br>$p = .15$
HUM → DWB → RWA	-.101[-.178, -.046]<br>$p < .001$	-.096[-.222, .000]<br>$p = .050$	-.028[-.148, .067]<br>$p = .57$
HUM → SDO → LR	-.449[-.683, -.259]<br>$p < .001$	-.253[-.445, -.131]<br>$p = .001$	-.319[-.741, -.050]<br>$p = .021$
HUM → RWA → LR	-.418[-.681, -.234]<br>$p < .001$	-.335[-.627, -.134]<br>$p < .001$	-.073[-.604, .278]<br>$p = .65$
HUM → CWB → LR	-.182[-.315, -.092]<br>$p < .001$	.090[-.023, .520]<br>$p = .13$	-.174[-.499, .019]<br>$p = .077$
HUM → DWB → LR	-.094[-.174, -.041]<br>$p < .001$	-.051[-.129, .004]<br>$p = .063$	.007[-.024, .123]<br>$p = .42$
NORM → CWB → SDO	.272[.165, .423]<br>$p < .001$	.325[.080, .571]<br>$p = .023$	.378[.191, .652]<br>$p = .001$
NORM → DWB → SDO	.013[-.001, .031]<br>$p = .060$	.048[-.010, .173]<br>$p = .11$	.033[-.006, .082]<br>$p = .092$
NORM → CWB → RWA	.027[.008, .055]<br>$p = .005$	.030[-.068, .093]<br>$p = .34$	.035[-.025, .093]<br>$p = .22$
NORM → DWB → RWA	.142[.083, .223]<br>$p < .001$	.535[.193, 1.01]<br>$p < .001$	.394[.283, .567]<br>$p < .001$
NORM → SDO → LR	.194[.100, .328]<br>$p < .001$	.216[.098, .499]<br>$p = .001$	.563[.046, 1.20]<br>$p = .033$
NORM → RWA → LR	.314[.176, .501]<br>$p < .001$	1.05[.307, 1.94]<br>$p < .001$	1.54[.710, 3.04]<br>$p = .001$
NORM → CWB → LR	.185[.095, .315]<br>$p < .001$	-.085[-.596, .022]<br>$p = .14$	.951[.437, 1.69]<br>$p = .001$
NORM → DWB → LR	.132[.073, .216]<br>$p < .001$	.286[.127, .619]<br>$p < .001$	-.101[-.512, .228]<br>$p = .56$

Note. HUM = Humanism; NORM = Normativism; CWB = Competitive-world beliefs; DWB = Dangerous-world beliefs; SDO = Social dominance orientation; RWA = Right-wing authoritarianism; LR = Left-right self-placement.

Supplementary Table 3. Indirect relations based on the model incorporating HEXACO personality traits.

	Unstandardized Indirect effect<br>Study 4
HUM → O → SJ	.001[-.122, .086]<br>*p* = .94
HUM → E → SJ	-.147[-.365, -.020]<br>*p* = .025
HUM → H → SJ	-.088[-.250, -.014]<br>*p* = .021
HUM → O → PFE	-.001[-.086, .072]<br>*p* = .93
HUM → E → PFE	.156[.051, .334]<br>*p* = .006
HUM → H → PFE	.033[-.021, .168]<br>*p* = .20
HUM → O → RES	.000[-.089, .082]<br>*p* = .92
HUM → E → RES	.006[-.144, .150]<br>*p* = .96
HUM → H → RES	-.082[-.258, -.013]<br>*p* = .012
HUM → O → LR	.003[-.119, .085]<br>*p* = .90
HUM → E → LR	-.071[-.268, .078]<br>*p* = .29
HUM → H → LR	-.113[-.302, -.022]<br>*p* = .016
NORM → O → SJ	.137[.046, .295]<br>*p* = .005
NORM → E → SJ	-.051[-.222, .007]<br>*p* = .10
NORM → H → SJ	.098[.026, .247]<br>*p* = .013
NORM → O → PFE	-.094[-.274, -.010]<br>*p* = .025
NORM → E → PFE	.054[-.007, .187]<br>*p* = .084
NORM → H → PFE	-.037[-.162, .024]<br>*p* = .20
NORM → O → RES	.097[-.005, .253]

	p = .058
NORM → E → RES	.002[-.052, .066]<br>p = .79
NORM → H → RES	.092[.025, .260]<br>p = .006
NORM → O → LR	.125[.022, .299]<br>p = .020
NORM → E → LR	-.031[-.168, .007]<br>p = .11
NORM → H → LR	.123[.040, .290]<br>p = .010

Note. HUM = Humanism; NORM = Normativism; SJ = General system justification; PFE = Preference for equality; RES = Resistance to change; O = Openness; E = Emotionality; H = Honesty; LR = Left-right self-placement.
