# Supplementary material for: Rediscovering Tomkins’ polarity theory: Humanism, normativism, and the psychological basis of left-right ideological conflict in the U.S. and Sweden
Source: PLoS One. 2020 Jul 31;15(7):e0236627. doi: 10.1371/journal.pone.0236627 (PMC7394437; doi:10.1371/journal.pone.0236627)
Supplement: S1 Data — This is a zip archive that contains all of the data sets in csv-format and a codebook. (ZIP) [file pone.0236627.s004.zip › Codebook.docx]

**Codebook**

* = Reversed item

**Study 1**

---- Humanism ----

HH1: “All persons are in themselves valuable.”

HH2: “Human beings are basically good.”

HH3: “People are basically kind and helpful.”

HH4: “All human beings have an inner potential that they strive to realize.”

HH5: “Human beings are from the start good, even though bad circumstances can make them do bad things.”

HH6: “Although there is good and bad in people, humanity as a whole is basically good.”

HH7: “Human nature is basically good”

HH8: “When people do good deeds it is almost always out of genuine compassion and care for others.”

HI1: “Human beings should be loved at all times, because they want and need to be loved.”

HI2: “Human beings should be treated with respect at all times.”

HI3: “When people are in trouble, they need help and should be helped.”

HI4: “Children must be loved so that they can grow up to be fine adults.”

HI5: “To assume that most people are well-meaning brings out the best in others.”

HI6: “Those who err should be forgiven.”

HI7: “No one has the right to humiliate another person.”

HI8: “All persons deserve to be loved.”

HA1: “Feelings are the most important aspect of being human, because they give our lives meaning.”

HA2: “You need to be open to your feelings so that you can learn from them and understand who you are.”

HA3: “You must always leave yourself open to your own feelings – alien as they may sometimes seem.”

HA4: “There is a unique avenue to reality through the feelings, even when they seem alien.”

HA5: “The changeableness of human feelings makes life more interesting.”

HA6: “People should try to look inward to understand and accept their feelings as they are.”

HA7: “Feelings provide the most important guidance to a person’s decisions.”

HA8: “You should go with you feelings so that you do not have to look back in regret for holding back from what you really wanted.”

HE1: “The main purpose of education should be to enable the young to discover and create novelty.”

HE2: “Creativity and curiosity are the most important tools in the search for knowledge.”

HE3: “The important thing in science is to strike out into the unknown – right or wrong.”

HE4: “Personal imagination and understanding is crucial to the pursuit of knowledge.”

HE5: “A scientist must rely on creativity and intuition.”

HE6: “Learning must always start from your personal interests and experiences.”

HE7: “Great achievements require first of all great imagination.”

HE8: “Personal experiences can provide insights about reality that science cannot explain.”

HS1: “The most important purpose of society is to protect people’s rights, freedoms, and dignity.”

HS2: “The most important goal for a society is to make sure that all its members have a chance to lead a good life.”

HS3: “It is necessary to break the laws and rules of society when these lead to unfair treatment of some people.”

HS4: “The most important function of the government is to make sure people are treated in a just and dignified way.”

HS5: “Promotion of the welfare of the people is the most important function of a government.”

HS6: “We have to question the rules of the society when the well-being of individuals is threatened.”

HS7: “Society should encourage people to express themselves and follow their own desires.”

HS8: “Societies that violate human freedoms and rights must be vigorously questioned.”

---- Normativism ----

NH1: “When people do good deeds, it is almost always out of an expectation to receive something in return.”

NH2: “The bad people in the world outnumber the good people.”

NH3: “People don’t really care what happens to the next person.”

NH4: “People are naturally unfriendly and unkind.”

NH5: “Human beings are from the start primitive and egoistic animals that must be disciplined by society.”

NH6: “Human beings are basically evil.”

NH7: “Juvenile delinquency is simply a reflection of the basic evil in human beings – it has always existed in the past and it always will.”

NH8: “A person can only realize her-/himself by attaining external ideals.”

NI1: “Human beings should be treated with respect only when they deserve respect.”

NI2: “When people are in trouble, they should help themselves and not depend on others.”

NI3: “Human beings should be loved only when they have acted so that they deserve to be loved.”

NI4: “Some people respond only to punishment or the threat of punishment.”

NI5: “It is necessary to be hard and cold hearted toward other people when they deserve it.”

NI6: “Some people can only be changed by humiliating them.”

NI7: “When a person feels sorry for himself he should really feel ashamed of himself.”

NI8: “To assume that most people are well-meaning is asking for trouble.”

NA1: Human beings would be lost without reason, because feelings cannot be trusted.”

NA2: “Feelings must be controlled by reason, because they can make you do stupid things.”

NA3: “Feelings are often an obstacle to seeing how things really are.”

NA4: “You need to be wary of feelings, because they can hurt you and make you feel miserable.”

NA5: “If sanity is to be preserved, you must guard yourself against the intrusion of feelings which are alien to your nature.”

NA6: “The changeableness of human feelings is a weakness in human beings.”

NA7: “Going with your feelings often makes you unhappy in the long run.”

NA8: “There is no surer road to insanity than surrender to the feelings, particularly those which are alien to the self.”

NE1: “The most important task for a scientist is to collect facts about reality through objective observation.”

NE2: “Reason has to be continually disciplined and corrected by reality and hard facts.”

NE3: “To observe objectively and describe in a neutral language is crucial to the pursuit of knowledge.”

NE4: “The trouble with theorizing is that it leads people away from the facts and substitutes opinions for truth.”

NE5: “Observing the world accurately enables human beings to separate reality from imagination.”

NE6: “Discipline and rigour are the most important tools in the search for knowledge.”

NE7: “Imagination leads people into self-deception and delusions.”

NE8: “Education should focus on facts rather than theories.”

NS1: “The maintenance of law and order is the most important duty of any government.”

NS2: “People who commit crimes must be punished severely so that they are deterred from repeating the crime.”

NS3: “A society must enforce its laws and rules strictly in order not to deteriorate.”

NS4: “In order for society to work, there must be clear and fixed rules, and punishment for transgressions.”

NS5: “The most important function of society is to keep people’s destructive impulses under control with laws and rules.”

NS6: “Anger should be directed at those revolutionaries who undermine law and order.”

NS7: “Society should not encourage deviant and unwholesome activities.”

NS8: “It is often necessary to punish people severely in order to get them to conform to the social order.”

---- RWA ----

rwa1: “Many good people challenge the state, criticize the church and ignore ‘‘the normal way of living.”*

rwa2: “There are many radical, immoral people trying to ruin things; the society ought to stop them.”

rwa3: “People ought to put less attention to the Bible and religion, instead they ought to develop their own moral standards.”*

rwa4: “Facts show that we have to be harder against crime and sexual immorality, in order to uphold law and order.”

rwa5: “Our forefathers ought to be honored more for the way they have built our society, at the same time we ought to put an end to those forces destroying it.”

rwa6: “If the society so wants, it is the duty of every true citizen to help eliminate the evil that poisons our country from within.”

rwa7: “Our society would be better off if we showed tolerance and understanding for untraditional values and opinions.”*

rwa8: “It would be best if newspapers were censored so that people would not be able to get hold of destructive and disgusting material.”

rwa9: “The situation in the society of today would be improved if troublemakers were treated with reason and humanity.”*

rwa10: “God’s laws about abortion, pornography and marriage must be strictly followed before it is too late, violations must be punished.”

rwa11: “The society needs to show openness towards people thinking differently, rather than a strong leader, the world is not particularly evil or dangerous.”*

rwa12: “Our country needs free thinkers, who will have the courage to stand up against traditional ways, even if this upsets many people.”*

rwa13: “The ‘‘old-fashioned ways’’ and ‘‘old-fashioned values’’ still show the best way to live.”

rwa14: “It is better to accept bad literature than to censor it.”*

rwa15: “Our country needs a powerful leader, in order to destroy the radical and immoral currents prevailing in society today.”

---- SDO ----

sdo1: “We would have fewer problems if we treated people more equally.”*

sdo2: “Inferior groups should stay in their place.”

sdo3: “To get ahead in life, it is sometimes necessary to step on other groups.”

sdo4: “We should do what we can to equalize conditions for different groups.”*

sdo5: “In getting what you want, it is sometimes necessary to use force against other groups.”

sdo6: “Some groups of people are simply inferior to others.”

sdo7: “Group equality should be our ideal.”*

sdo8: “We should strive to make incomes as equal as possible.”*

---- Economic system justification ----

esj1: “Everyone has a fair shot at wealth and happiness.”

esj2: “Most people who don't get ahead in our society should not blame the system; they have only themselves to blame.”

esj3: “Economic differences in the society reflect an illegitimate distribution of resources.”*

esj4: “Social class differences reflect differences in the natural order of things.”

esj5: “Economic positions are legitimate reflections of people's achievements.”

esj6: “Society is set up so that people usually get what they deserve. If people work hard, they almost always get what they want.”

esj7: “There is no point in trying to make incomes more equal.”

ejs8: “It is unfair to have an economic system which produces extreme wealth and extreme poverty at the same time.”*

---- Dangerous-world beliefs ----

dw1: “Any day now chaos and anarchy could erupt around us. All signs are pointing to it.”

dw2: “There are many dangerous people in our society who will attack someone out of pure meanness, for no reason at all.”

dw3: “Despite what one hears about “crime on the street”, there probably isn’t any more now than there ever has been.”*

dw4: “If a person takes a few sensible precautions, nothing bad is likely to happen to him or her; we do not live in a dangerous world.”*

dw5: “Every day as society becomes more lawless and bestial; a person’s chances of being robbed, assaulted, and even murdered go up and up.”

dw6: “My knowledge and experiences tell me that the social world we live in is basically a safe, stable, and secure place in which most people are fundamentally good.”*

dw7: “It seems that every year there are fewer and fewer truly respectable people, and more and more persons with no morals at all who threaten everyone else.”

dw8: “My knowledge and experience tell me that the social world we live in is basically a dangerous and unpredictable place, in which good, decent, and moral people’s values and way of life are threatened and disrupted by bad people.”

dw9: “The end is not near. People who think that earthquakes, wars, and famines mean God might be about to destroy the world are being foolish.”*

dw10: “Although it may appear that things are constantly getting more dangerous and chaotic, it really isn’t so. Every era has its problems, and a person’s chances of living a safe, untroubled life are better today than ever before.”*

---- Competitive-world beliefs ----

cw1: “Winning is not the first thing; it’s the only thing.”

cw2: “The best way to lead a group under one’s supervision is to show them kindness, consideration, and treat them as fellow workers, not as inferiors.”*

cw3: “If it’s necessary to be cold blooded and vengeful to reach one’s goals, then one should do it.”

cw4: “Life is not governed by the “survival of the fittest.” We should let compassion and moral laws be our guide.”*

cw5: “Money, wealth, and luxury are what really count in life.”

cw6: “It is much more important in life to have integrity in your dealings with others than to have money and power.”*

cw7: “It’s a dog eat dog world where you have to be ruthless at all times.”

cw8: “You know that most people are out to “screw” you; so you have to get them first when you get a chance.”

cw9: “My knowledge and experience tells me that the social world we live in is basically a “competitive jungle” in which the fittest survive and succeed; power, wealth, and winning are everything; and might is right.”

cw10: “We can make a society based on unselfish cooperation, sharing, and people generously helping each other, and not on competition and acquisitiveness.”*

---- Insecure attachment ----

a1: “I get frustrated if romantic partners are not available when I need them”

a2: “I need a lot of reassurance that I am loved by my partner.”

a3: “I worry that romantic partners won’t care about me as much as I care about them.”

a4: “I want to get close to my partner, but I keep pulling back.”

a5:” My desire to be very close sometimes scares people away.”

a6: “I turn to my partner for many things, including comfort and reassurance.”*

a7: “I do not often worry about being abandoned.”*

a8: “I usually discuss my problems and concerns with my partner.”*

a9: “I try to avoid getting too close to my partner.”

a10: “I find that my partner(s) don’t want to get as close as I would like.”

a11: “I am nervous when partners get too close to me.”

a12: “It helps to turn to my romantic partner in times of need.”*

---- Death anxiety ----

ex1: “The prospect of my own death arouses anxiety in me.”

ex2: “I always try not to think about death.”

ex3: “Death is no doubt a grim experience.”

ex4: “I try to have nothing to do with the subject of death.”

---- Need for closure ----

nc1: “I think that having clear rules and order at work is essential for success.”

nc2: “Even after I've made up my mind about something, I am always eager to consider a different opinion.”*

nc3: “I don't like situations that are uncertain.”

nc4: “I dislike questions which could be answered in many different ways.”

nc5: “I like to have friends who are unpredictable. “

nc6: “I find that a well ordered life with regular hours suits my temperament.”

nc7: “When dining out, I like to go to places where I have been before so that I know what to expect.”

nc8: “I feel uncomfortable when I don't understand the reason why an event occurred in my life.”

nc9: “I feel irritated when one person disagrees with what everyone else in a group believes.”

nc10: “I hate to change my plans at the last minute.”

nc11: “I don't like to go into a situation without knowing what I can expect from it.”

nc12: “When I go shopping, I have difficulty deciding exactly what it is that I want.”*

nc13: “When faced with a problem I usually see the one best solution very quickly.”

nc14: “When I am confused about an important issue, I feel very upset.”

nc15: “I tend to put off making important decisions until the last possible moment.”*

nc16: “I usually make important decisions quickly and confidently.”

nc17: “I would describe myself as indecisive.”*

nc18: “I think it is fun to change my plans at the last moment.”*

nc19: “I enjoy the uncertainty of going into a new situation without knowing what might happen.”*

nc20: “My personal space is usually messy and disorganized.”*

nc21: “In most social conflicts, I can easily see which side is right and which is wrong.”

nc22: “I tend to struggle with most decisions.”*

nc23: “I believe that orderliness and organization are among the most important characteristics of a good student.”

nc24: “When considering most conflict situations, I can usually see how both sides could be right.”*

nc25: “I don't like to be with people who are capable of unexpected actions.”

nc26: “I prefer to socialize with familiar friends because I know what to expect from them.”

nc27: “I think that I would learn best in a class that lacks clearly stated objectives and requirements.”*

nc28: “When thinking about a problem, I consider as many different opinions on the issue as possible.”*

nc29: “I like to know what people are thinking all the time.”

nc30: “I dislike it when a person's statement could mean many different things.”

nc31: “It's annoying to listen to someone who cannot seem to make up his or her mind.”

nc32: “I find that establishing a consistent routine enables me to enjoy life more.”

nc33: “I enjoy having a clear and structured mode of life.”

nc34: “I prefer interacting with people whose opinions are very different from my own.”*

nc35: “I like to have a place for everything and everything in its place.”

nc36: “I feel uncomfortable when someone's meaning or intention is unclear to me.”

nc37: “When trying to solve a problem I often see so many possible options that it's confusing.”*

nc38: “I always see many possible solutions to problems I face.”*

nc39: “I'd rather know bad news than stay in a state of uncertainty.”

nc40: “I do not usually consult many different opinions before forming my own view.”

nc41: “I dislike unpredictable situations.”

nc42: “I dislike the routine aspects of my work (studies).”*

---- General system justification ----

sj1: “In general, I find society to be fair.”

sj2: “In general, the American political system operates as it should.”

sj3: “American society needs to be radically restructured.”*

sj4: “The United States is the best country in the world to live in.”

sj5: “Most policies serve the greater good.”

sj6: “Everyone has a fair shot at wealth and happiness.”

sj7: “Our society is getting worse every year.”*

sj8: “Society is set up so that people usually get what they deserve.”

---- Resistance to change ----

res1: “Young people sometimes get rebellious ideas, but as they grow up they ought to get over them and settle down.”

res2: “This country would be better off if there were more emphasis on traditional family ties.”

res3: “Our customs and national heritage are the things that have made us great, and some people should show greater respect for them.”

res4: “If you start changing things very much, you often end up making them worse.”

res5: “Changing any institution (e.g., government, religion, business) is risky, so it is better to change at a slow than a rapid pace.”

res6: “If something is wrong with an institution (e.g., government, religion, business), it is necessary to make immediate changes.”*

res7: “The answers for today's questions can often be found in the traditions and customs of the past.”

res8: “Looking back, life was much better in the past.”

res9: “The ‘‘old-fashioned ways’’ and ‘‘old-fashioned values’’ still show the best way to live.”

res10: “Our society would be better off if we showed tolerance and understanding for untraditional values and opinions.”*

res11: “If something grows up over a long time, there will usually be much wisdom in it.”

---- Preference for equality ----

pfe1: “Companies should be held accountable for promoting racial diversity and a tolerant atmosphere in their offices and corporate culture.”

pfe2: “The government should take more measures to eliminate economic disparities between men and women who are doing the same work.”

pfe3: “It is the responsibility of the government to take care of people who can’t take care of themselves.”

pfe4: “We need to take care of the poor and disadvantaged before helping the rest of the country.”

pfe5: “We should find ways to help others less fortunate than ourselves.”

pfe6: “Prosperous nations have a moral obligation to share some of their wealth with poor nations.”

pfe7: “Whether we like it or not, some people are just more worthy than others.”*

pfe8: “Large fortunes and estates should be taxed fairly heavily over and above income taxes.”

pfe9: “A person should always be concerned about the well-being of others.”

---- Single-item variables ----

Version: 1 = Completed the study in a lab; 2 = Completed the study online

ex5: “Our way of life is seriously threatened by the forces of terrorism in the world.”

Gender: 1 = Man, 2 = Woman

Age

BirthCountry: Open question

LeftRight: “Where would you place yourself on the following scale?”

LeftRightEco: “In terms of economic issues, where would you place yourself on the following scale?”

LeftRightSoc: “In terms of social and cultural issues, where would you place yourself on the following scale?”

---- Scales ----

Humanism

Normativism

RWA: Right-wing authoritarianism

SDO: Social dominance orientation

DWB: Dangerous-world beliefs

CWB: Competitive-world beliefs

EcoSJ: Economic system justification

DeathAnxiety: Death anxiety

InsecureAttachment: Insecure attachment

RC: Resistance to change

PFE: Preference for equality

SJ: General system justification

NFC: Need for closure

**Study 2**

---- Humanism ----

H1: “All persons are in themselves valuable.”

H2: “Human beings should be treated with respect at all times.”

H3: “It is necessary to break the laws and rules of society when these lead to unfair treatment of some people.”

H4: “Human beings are basically good.”

H5: “When people are in trouble, they need help and should be helped.”

H6: “The most important purpose of society is to protect people’s rights, freedoms, and dignity.”

H7: “The main purpose of education should be to enable the young to discover and create novelty.”

H8: “The important thing in science is to strike out into the unknown – right or wrong.” (excluded from the scale^[[1]](#footnote-1)^)

H9: “Human beings should be loved at all times, because they want and need to be loved.”

H10: “The most important goal for a society is to make sure that its members have a chance to lead a good life.”

H11: “Feelings are the most important aspect of being human, because they give our lives meaning.”

H12: “A scientist must rely on creativity and intuition.”

H13: “You need to be open to your feelings to learn from them and understand who you are.”

H14: “You must always leave yourself open to your own feelings – alien as they may sometimes seem.”

H15: “People are basically kind and helpful.”

H16: “Creativity and curiosity are the most important tools in the search for knowledge.”

---- Normativism ----

N1: “The maintenance of law and order is the most important duty of any government.”

N2: “When people are in trouble, they should help themselves and not depend on others.”

N3: “Human beings would be lost without reason, because feelings cannot be trusted.”

N4: “People don’t really care what happens to the next person.”

N5: “Feelings are often an obstacle to seeing how things really are.”

N6: “When people do good deeds, it is almost always out of an expectation to receive something in return.”

N7: “To observe objectively and describe in a neutral language is crucial to the pursuit of knowledge.”

N8: “Human beings should be treated with respect only when they deserve respect.”

N9: “A society must enforce its laws and rules strictly in order not to deteriorate.”

N10: “Feelings must be controlled by reason, because they can make you do stupid things.”

N11: “The most important task for a scientist is to collect facts about reality through objective observation.” (excluded from the scale)

N12: “Imagination leads people into self-deception and delusions.”

N13: “People who commit crimes must be punished severely so that they are deterred from repeating the crime.”

N14: “Human beings should be loved only when they have acted so that they deserve to be loved.”

N15: “The bad people in the world outnumber the good people.”

N16: “Reason has to be continually disciplined and corrected by reality and hard facts.”

---- RWA ----

rwa1: “Many good people challenge the state, criticize the church and ignore ‘‘the normal way of living.”*

rwa2: “There are many radical, immoral people trying to ruin things; the society ought to stop them.”

rwa3: “People ought to put less attention to the Bible and religion, instead they ought to develop their own moral standards.”*

rwa4: “Facts show that we have to be harder against crime and sexual immorality, in order to uphold law and order.”

rwa5: “Our forefathers ought to be honored more for the way they have built our society, at the same time we ought to put an end to those forces destroying it.”

rwa6: “If the society so wants, it is the duty of every true citizen to help eliminate the evil that poisons our country from within.”

rwa7: “Our society would be better off if we showed tolerance and understanding for untraditional values and opinions.”*

rwa8: “It would be best if newspapers were censored so that people would not be able to get hold of destructive and disgusting material.”

rwa9: “The situation in the society of today would be improved if troublemakers were treated with reason and humanity.”*

rwa10: “God’s laws about abortion, pornography and marriage must be strictly followed before it is too late, violations must be punished.”

rwa11: “The society needs to show openness towards people thinking differently, rather than a strong leader, the world is not particularly evil or dangerous.”*

rwa12: “Our country needs free thinkers, who will have the courage to stand up against traditional ways, even if this upsets many people.”*

rwa13: “The ‘‘old-fashioned ways’’ and ‘‘old-fashioned values’’ still show the best way to live.”

rwa14: “It is better to accept bad literature than to censor it.”*

rwa15: “Our country needs a powerful leader, in order to destroy the radical and immoral currents prevailing in society today.”

---- SDO ----

sdo1: “We would have fewer problems if we treated people more equally.”*

sdo2: “Inferior groups should stay in their place.”

sdo3: “To get ahead in life, it is sometimes necessary to step on other groups.”

sdo4: “We should do what we can to equalize conditions for different groups.”*

sdo5: “In getting what you want, it is sometimes necessary to use force against other groups.”

sdo6: “Some groups of people are simply inferior to others.”

sdo7: “Group equality should be our ideal.”*

sdo8: “We should strive to make incomes as equal as possible.”*

---- Economic system justification ----

esj1: “Everyone has a fair shot at wealth and happiness.”

esj2: “Most people who don't get ahead in our society should not blame the system; they have only themselves to blame.”

esj3: “Economic differences in the society reflect an illegitimate distribution of resources.”*

esj4: “Social class differences reflect differences in the natural order of things.”

esj5: “Economic positions are legitimate reflections of people's achievements.”

esj6: “Society is set up so that people usually get what they deserve. If people work hard, they almost always get what they want.”

esj7: “There is no point in trying to make incomes more equal.”

ejs8: “It is unfair to have an economic system which produces extreme wealth and extreme poverty at the same time.”*

---- Dangerous-world beliefs ----

dw1: “Any day now chaos and anarchy could erupt around us. All signs are pointing to it.”

dw2: “There are many dangerous people in our society who will attack someone out of pure meanness, for no reason at all.”

dw3: “Despite what one hears about “crime on the street”, there probably isn’t any more now than there ever has been.”*

dw4: “If a person takes a few sensible precautions, nothing bad is likely to happen to him or her; we do not live in a dangerous world.”*

dw5: “Every day as society becomes more lawless and bestial; a person’s chances of being robbed, assaulted, and even murdered go up and up.”

dw6: “My knowledge and experiences tell me that the social world we live in is basically a safe, stable, and secure place in which most people are fundamentally good.”*

dw7: “It seems that every year there are fewer and fewer truly respectable people, and more and more persons with no morals at all who threaten everyone else.”

dw8: “My knowledge and experience tell me that the social world we live in is basically a dangerous and unpredictable place, in which good, decent, and moral people’s values and way of life are threatened and disrupted by bad people.”

dw9: “The end is not near. People who think that earthquakes, wars, and famines mean God might be about to destroy the world are being foolish.”*

dw10: “Although it may appear that things are constantly getting more dangerous and chaotic, it really isn’t so. Every era has its problems, and a person’s chances of living a safe, untroubled life are better today than ever before.”*

---- Competitive-world beliefs ----

cw1: “Winning is not the first thing; it’s the only thing.”

cw2: “The best way to lead a group under one’s supervision is to show them kindness, consideration, and treat them as fellow workers, not as inferiors.”*

cw3: “If it’s necessary to be cold blooded and vengeful to reach one’s goals, then one should do it.”

cw4: “Life is not governed by the “survival of the fittest.” We should let compassion and moral laws be our guide.”*

cw5: “Money, wealth, and luxury are what really count in life.”

cw6: “It is much more important in life to have integrity in your dealings with others than to have money and power.”*

cw7: “It’s a dog eat dog world where you have to be ruthless at all times.”

cw8: “You know that most people are out to “screw” you; so you have to get them first when you get a chance.”

cw9: “My knowledge and experience tells me that the social world we live in is basically a “competitive jungle” in which the fittest survive and succeed; power, wealth, and winning are everything; and might is right.”

cw10: “We can make a society based on unselfish cooperation, sharing, and people generously helping each other, and not on competition and acquisitiveness.”*

---- General system justification ----

sj1: “In general, I find society to be fair.”

sj2: “In general, the American political system operates as it should.”

sj3: “American society needs to be radically restructured.”*

sj4: “The United States is the best country in the world to live in.”

sj5: “Most policies serve the greater good.”

sj6: “Everyone has a fair shot at wealth and happiness.”

sj7: “Our society is getting worse every year.”*

sj8: “Society is set up so that people usually get what they deserve.”

---- Resistance to change ----

res1: “Young people sometimes get rebellious ideas, but as they grow up they ought to get over them and settle down.”

res2: “This country would be better off if there were more emphasis on traditional family ties.”

res3: “Our customs and national heritage are the things that have made us great, and some people should show greater respect for them.”

res4: “If you start changing things very much, you often end up making them worse.”

res5: “Changing any institution (e.g., government, religion, business) is risky, so it is better to change at a slow than a rapid pace.”

res6: “If something is wrong with an institution (e.g., government, religion, business), it is necessary to make immediate changes.”*

res7: “The answers for today's questions can often be found in the traditions and customs of the past.”

res8: “Looking back, life was much better in the past.”

res9: “The ‘‘old-fashioned ways’’ and ‘‘old-fashioned values’’ still show the best way to live.”

res10: “Our society would be better off if we showed tolerance and understanding for untraditional values and opinions.”*

res11: “If something grows up over a long time, there will usually be much wisdom in it.”

---- Preference for equality ----

pfe1: “Companies should be held accountable for promoting racial diversity and a tolerant atmosphere in their offices and corporate culture.”

pfe2: “The government should take more measures to eliminate economic disparities between men and women who are doing the same work.”

pfe3: “It is the responsibility of the government to take care of people who can’t take care of themselves.”

pfe4: “We need to take care of the poor and disadvantaged before helping the rest of the country.”

pfe5: “We should find ways to help others less fortunate than ourselves.”

pfe6: “Prosperous nations have a moral obligation to share some of their wealth with poor nations.”

pfe7: “Whether we like it or not, some people are just more worthy than others.”*

pfe8: “Large fortunes and estates should be taxed fairly heavily over and above income taxes.”

pfe9: “A person should always be concerned about the well-being of others.”

---- Conservative issue preferences ----

i1: Abortion

i2: Welfare benefits*

i3: Limited government

i4: Military and national security

i5: Religion

i6: Gun ownership

i7: Traditional marriage

i8: Traditional values

i9: Fiscal responsibility

i10: Business

i11: The family unit

i12: Patriotism

i13: Labor unions*

i14: Gay Marriage*

i15: Affirmative Action*

i16: The death penalty

---- Insecure attachment ----

a1: “I get frustrated if romantic partners are not available when I need them.”

a2: “I need a lot of reassurance that I am loved by my partner.”

a3: “I worry that romantic partners won't care about me as much as I care about them.”

a4: “I want to get close to my partner, but I keep pulling back.”

a5: “My desire to be very close sometimes scares people away.”

a6: “I turn to my partner for many things, including comfort and reassurance.”*

a7: “I do not worry about being abandoned.”*

a8: “I usually discuss my problems and concerns with my partner.”*

a9: “I try to avoid getting too close to my partner.”

a10: “I find that my partner(s) don't want to get as close as I would like.”

a11: “I am nervous when partners get too close to me.”

a12: “It helps to turn to my romantic partner in times of need.”*

---- Need for closure ----

nfc1: “I don't like situations that are uncertain.”

nfc2: “I dislike questions which could be answered in many different ways.”

nfc3: “I find that a well ordered life with regular hours suits my temperament.”

nfc4: “I feel uncomfortable when I don't understand the reason why an event occurred in my life.”

nfc5: “I feel irritated when one person disagrees with what everyone else in a group believes.”

nfc6: “I don't like to go into a situation without knowing what I can expect from it.”

nfc7: “When I have made a decision, I feel relieved.”

nfc8: “When I am confronted with a problem, I'm dying to reach a solution very quickly.”

nfc9: “I would quickly become impatient and irritated if I would not find a solution to a problem immediately.”

nfc10: “I don't like to be with people who are capable of unexpected actions.”

nfc11: “I dislike it when a person's statement could mean many different things.”

nfc12: “I find that establishing a consistent routine enables me to enjoy life more.”

nfc13: “I enjoy having a clear and structured mode of life.”

nfc14: “I do not usually consult many different opinions before forming my own view.”

nfc15: “I dislike unpredictable situations.”

---- Death anxiety ----

ex1: “Death is no doubt a grim experience.”

ex2: “I try to have nothing to do with the subject of death.”

ex3: “The prospect of my own death arouses anxiety in me.”

ex4: “I always try not to think about death.”

---- Single-item variables ----

ex5: “Our way of life is seriously threatened by the forces of terrorism in the world.”

Gender: 1 = Man, 2 = Woman

Age

Occupation: Open question

Education: Open question, recoded into 1 = Secondary school, 2 = College, no degree, 3 = Associates degree, 4 = College degree, 5 = BA, 6 = MA, 7 = PhD

BirthCountry: Open question

LeftRight: “Where would you place yourself on the following scale?”

LeftRightEco: “In terms of economic issues, where would you place yourself on the following scale?”

LeftRightSoc: “In terms of social and cultural issues, where would you place yourself on the following scale?”

---- Scales ----

Humanism

Normativism

RWA: Right-wing authoritarianism

SDO: Social dominance orientation

DWB: Dangerous-world beliefs

CWB: Competitive-world beliefs

RC: Resistance to change

PFE: Preference for equality

SJ: General system justification

EcoSJ: Economic system justification

IssuePrefs: Conservative issue preferences

NFC: Need for closure

DeathAnxiety: Death anxiety

InsecureAttachment: Insecure attachment

**Study 3**

---- Humanism ----

H1: “All persons are in themselves valuable.”

H2: “Feelings are the most important aspect of being human, because they give our lives meaning.”

H3: “The most important purpose of society is to protect people’s rights, freedoms, and dignity.”

H4: “People are basically kind and helpful.”

H5: “Human beings should be loved at all times, because they want and need to be loved.”

H6: “It is necessary to break the laws and rules of society when these lead to unfair treatment of some people.”

H7: “The main purpose of education should be to enable the young to discover and create novelty.”

H8: “You must always leave yourself open to your own feelings – alien as they may sometimes seem.”

H9: “Human beings should be treated with respect at all times.”

H10: “The important thing in science is to strike out into the unknown – right or wrong.”

H11: “You need to be open to your feelings to learn from them and understand who you are.”

H12: “Creativity and curiosity are the most important tools in the search for knowledge.”

H13: “Human beings are basically good.”

H14: “When people are in trouble, they need help and should be helped.”

H15: “The most important goal for a society is to make sure that its members have a chance to lead a good life.”

---- Normativism ----

N1: “Reason has to be continually disciplined and corrected by reality and hard facts.”

N2: “People don’t really care what happens to the next person.”

N3: “Human beings would be lost without reason, because feelings cannot be trusted.”

N4: “Human beings should be treated with respect only when they deserve respect.”

N5: “People who commit crimes must be punished severely so that they are deterred from repeating the crime.”

N6: “Feelings must be controlled by reason, because they can make you do stupid things.”

N7: “When people do good deeds, it is almost always out of an expectation to receive something in return.”

N8: “When people are in trouble, they should help themselves and not depend on others.”

N9: “The most important task for a scientist is to collect facts about reality through objective observation.”

N10: “The bad people in the world outnumber the good people.”

N11: “The maintenance of law and order is the most important duty of any government.”

N12: “To observe objectively and describe in a neutral language is crucial to the pursuit of knowledge.”

N13: “A society must enforce its laws and rules strictly in order not to deteriorate.”

N14: “Feelings are often an obstacle to seeing how things really are.”

N15: “Human beings should be loved only when they have acted so that they deserve to be loved.”

---- RWA ----

rwa1: “Many good people challenge the state, criticize the church and ignore ‘‘the normal way of living.”*

rwa2: “There are many radical, immoral people trying to ruin things; the society ought to stop them.”

rwa3: “People ought to put less attention to the Bible and religion, instead they ought to develop their own moral standards.”*

rwa4: “Facts show that we have to be harder against crime and sexual immorality, in order to uphold law and order.”

rwa5: “Our forefathers ought to be honored more for the way they have built our society, at the same time we ought to put an end to those forces destroying it.”

rwa6: “If the society so wants, it is the duty of every true citizen to help eliminate the evil that poisons our country from within.”

rwa7: “Our society would be better off if we showed tolerance and understanding for untraditional values and opinions.”*

rwa8: “It would be best if newspapers were censored so that people would not be able to get hold of destructive and disgusting material.”

rwa9: “The situation in the society of today would be improved if troublemakers were treated with reason and humanity.”*

rwa10: “God’s laws about abortion, pornography and marriage must be strictly followed before it is too late, violations must be punished.”

rwa11: “The society needs to show openness towards people thinking differently, rather than a strong leader, the world is not particularly evil or dangerous.”*

rwa12: “Our country needs free thinkers, who will have the courage to stand up against traditional ways, even if this upsets many people.”*

rwa13: “The ‘‘old-fashioned ways’’ and ‘‘old-fashioned values’’ still show the best way to live.”

rwa14: “It is better to accept bad literature than to censor it.”*

rwa15: “Our country needs a powerful leader, in order to destroy the radical and immoral currents prevailing in society today.”

---- SDO ----

sdo1: “Some groups of people are simply inferior to others.”

sdo2: “In getting what you want, it is sometimes necessary to use force against other groups.”

sdo3: “To get ahead in life, it is sometimes necessary to step on other groups.”

sdo4: “Inferior groups should stay in their place.”

sdo5: “Group equality should be our ideal.”*

sdo6: “We should do what we can to equalize conditions for different groups.”*

sdo7: “We would have fewer problems if we treated people more equally.”*

sdo8: “We should strive to make incomes as equal as possible.”*

---- Dangerous-world beliefs ----

dw1: “My knowledge and experience tell me that the social world we live in is basically a dangerous and unpredictable place, in which good, decent, and moral people’s values and way of life are threatened and disrupted by bad people.”

dw2: “Although it may appear that things are constantly getting more dangerous and chaotic, it really isn’t so. Every era has its problems, and a person’s chances of living a safe, untroubled life are better today than ever before.”*

dw3: “Any day now chaos and anarchy could erupt around us. All signs are pointing to it.”

dw4: “There are many dangerous people in our society who will attack someone out of pure meanness, for no reason at all.”

dw5: “Despite what one hears about “crime on the street”, there probably isn’t any more now than there ever has been.”*

dw6: “If a person takes a few sensible precautions, nothing bad is likely to happen to him or her; we do not live in a dangerous world.”*

dw7: “Every day as society becomes more lawless and bestial; a person’s chances of being robbed, assaulted, and even murdered go up and up.”

dw8: “My knowledge and experiences tell me that the social world we live in is basically a safe, stable, and secure place in which most people are fundamentally good.”*

dw9: “It seems that every year there are fewer and fewer truly respectable people, and more and more persons with no morals at all who threaten everyone else.”

dw10: “The end is not near. People who think that earthquakes, wars, and famines mean God might be about to destroy the world are being foolish.”*

---- Competitive-world beliefs ----

cw1: “We can make a society based on unselfish cooperation, sharing, and people generously helping each other, and not on competition and acquisitiveness.”*

cw2: “The best way to lead a group under one’s supervision is to show them kindness, consideration, and treat them as fellow workers, not as inferiors.”*

cw3: “Winning is not the first thing; it’s the only thing.”

cw4: “If it’s necessary to be cold blooded and vengeful to reach one’s goals, then one should do it.”

cw5: “Life is not governed by the “survival of the fittest.” We should let compassion and moral laws be our guide.”*

cw6: “Money, wealth, and luxury are what really count in life.”

cw7: “It is much more important in life to have integrity in your dealings with others than to have money and power.”*

cw8: “It’s a dog eat dog world where you have to be ruthless at all times.”

cw9: “My knowledge and experience tells me that the social world we live in is basically a “competitive jungle” in which the fittest survive and succeed; power, wealth, and winning are everything; and might is right.”

cw10: “You know that most people are out to “screw” you; so you have to get them first when you get a chance.”

---- Need for closure ----

nfc1: “I don’t like situations that are uncertain.”

nfc2: “I dislike questions which could be answered in many different ways.”

nfc3: “I find that a well ordered life with regular hours suits my temperament.”

nfc4: “I feel uncomfortable when I don’t understand the reason why an event occurred in my life.”

nfc5: “I feel irritated when one person disagrees with what everyone else in a group believes.”

nfc6: “I don’t like to go into a situation without knowing what I can expect from it.”

nfc7: “When I have made a decision, I feel relieved.”

nfc8: “When I am confronted with a problem, I’m dying to reach a solution very quickly.”

nfc9: “I would quickly become impatient and irritated if I would not find a solution to a problem immediately.”

nfc10: “I don’t like to be with people who are capable of unexpected actions.”

nfc11: “I dislike it when a person’s statement could mean many different things.”

nfc12: “I find that establishing a consistent routine enables me to enjoy life more.”

nfc13: “I enjoy having a clear and structured mode of life.”

nfc14: “I do not usually consult many different opinions before forming my own view.”

nfc15: ”I dislike unpredictable situations.”

---- Death anxiety ---

ex1: “Coffins make me nervous.”

ex2: “The sight of a corpse deeply shocks me.”

ex3: “I would never accept a job in a funeral home.”

ex4: “I get upset when I am in a cemetery.”

ex5: “It annoys me to hear about death.”

ex6: “I avoid death thoughts at all costs.”

ex7: “Whenever the thought of death enters my mind, I try to push it away.”

ex8: “I have an intense fear of death.”

---- Single-item variables ----

Gender: 1 = Woman, 2 = Man

Age

Education: Total years of education

Income: 1 = Very high above average, 2 = Above average, 3 = A little above average, 4 = Average, 5 = A little below average, 6 = Below average, 7 = Very high above average

Religiosity: 1 = Not at all religious, 7 = Extremely religious

LeftRight: “In political matters, people sometimes talk about ‘the left’ and ‘the right.’ How would you place your views on this scale, generally speaking?”

---- Scales ----

Humanism

Normativism

RWA: Right-wing authoritarianism

SDO: Social dominance orientation

DWB: Dangerous-world beliefs

CWB: Competitive-world beliefs

NFC: Need for closure

DeathAnxiety: Death anxiety

**Study 4**

---- Humanism ----

H1: “All persons are in themselves valuable.”

H2: “Feelings are the most important aspect of being human, because they give our lives meaning.”

H3: “The most important purpose of society is to protect people’s rights, freedoms, and dignity.”

H4: “The most important goal for a society is to make sure that its members have a chance to lead a good life.”

H5: “Human beings should be loved at all times, because they want and need to be loved.”

H6: “When people do good deeds it is almost always out of genuine compassion and care for others.”

H7: “It is necessary to break the laws and rules of society when these lead to unfair treatment of some people.”

H8: “The main purpose of education should be to enable the young to discover and create novelty.”

H9: “You must always leave yourself open to your own feelings – alien as they may sometimes seem.”

H10: “Human beings should be treated with respect at all times.”

H11: “Personal experiences can provide insights about reality that science cannot explain.”

H12: “You need to be open to your feelings to learn from them and understand who you are.”

H13: “Creativity and curiosity are the most important tools in the search for knowledge.”

H14: “Human beings are basically good.”

H15: “When people are in trouble, they need help and should be helped.”

---- Normativism ----

N1: “Reason has to be continually disciplined and corrected by reality and hard facts.”

N2: “People don’t really care what happens to the next person.”

N3: “Human beings would be lost without reason, because feelings cannot be trusted.”

N4: “Human beings should be treated with respect only when they deserve respect.”

N5: “People who commit crimes must be punished severely so that they are deterred from repeating the crime.”

N6: “Feelings must be controlled by reason, because they can make you do stupid things.”

N7: “When people are in trouble, they should help themselves and not depend on others.”

N8: “The most important task for a scientist is to collect facts about reality through objective observation.”

N9: “The bad people in the world outnumber the good people.”

N10: “The maintenance of law and order is the most important duty of any government.”

N11: “To observe objectively and describe in a neutral language is crucial to the pursuit of knowledge.”

N12: “In order for society to work, there must be clear and fixed rules, and punishment for transgressions.”

N13: “You need to be wary of feelings, because they can hurt you and make you feel miserable.”

N14: “Human beings should be loved only when they have acted so that they deserve to be loved.”

N15: “Human beings are basically evil”.

---- General system justification ----

sj1: “Most policies serve the greater good.”

sj2: “Swedish society needs to be radically restructured.”*

sj3: “In general, the Swedish political system operates as it should.”

sj4: “Everyone has a fair shot at wealth and happiness.”

sj5: “In general, I find society to be fair.”

sj6: “Our society is getting worse every year.”*

sj7: “Sweden is the best country in the world to live in.”

sj8: “Society is set up so that people usually get what they deserve.”

---- Economic system justification ----

esj1: “Economic differences in the society reflect an illegitimate distribution of resources.”*

esj2: “Everyone has a fair shot at wealth and happiness.”

esj3: “Social class differences reflect differences in the natural order of things.”

esj4: “Economic positions are legitimate reflections of people's achievements.”

esj5: “Society is set up so that people usually get what they deserve. If people work hard, they almost always get what they want.”

---- Individualizing moral intuitions ----

ind1: “Whether or not someone suffered emotionally.”

ind2: “Whether or not some people were treated differently than others.”

ind3: “Whether or not someone cared for someone weak or vulnerable.”

ind4: “Whether or not someone acted unfairly.”

ind5: “Whether or not someone was cruel.”

ind6: “Whether or not someone was denied his or her rights.”

ind7: “Compassion for those who are suffering is the most crucial virtue.”

ind8: “When the government makes laws, the number one principle should be ensuring that everyone is treated fairly.”

ind9: “One of the worst things a person could do is hurt a defenseless animal.”

ind10: “Justice is the most important requirement for a society.”

ind11: “It can never be right to kill a human being.”

ind12: “I think it’s morally wrong that rich children inherit a lot of money while poor children inherit nothing.”

---- Binding moral intuitions ----

bind1: “Whether or not someone’s action showed love for his or her country.”

bind2: “Whether or not someone showed a lack of respect for authority.”

bind3: “Whether or not someone violated standards of purity and decency.”

bind4: “Whether or not someone did something to betray his or her group.”

bind5: “Whether or not someone conformed to the traditions of society.”

bind6: “Whether or not someone did something disgusting.”

bind7: “Whether or not someone showed a lack of loyalty.”

bind8: “Whether or not an action caused chaos or disorder.”

bind9: “Whether or not someone acted in a way that God would approve of.”

bind10: “I am proud of my country’s history.”

bind11: “Respect for authority is something all children need to learn.”

bind12: “People should not do things that are disgusting, even if no one is harmed.”

bind13: “People should be loyal to their family members, even when they have done something wrong.

bind14: “Men and women each have different roles to play in society.”

bind15: “I would call some acts wrong on the grounds that they are unnatural.”

bind16: “It is more important to be a team player than to express oneself.”

bind17: “If I were a soldier and disagreed with my commanding officer’s orders, I would obey anyway because that is my duty.”

bind18: “Chastity is an important and valuable virtue.”

---- Resistance to change ----

res1: “Young people sometimes get rebellious ideas, but as they grow up they ought to get over them and settle down.”

res2: “This country would be better off if there were more emphasis on traditional family ties.”

res3: “Our customs and national heritage are the things that have made us great, and some people should show greater respect for them.”

res4: “The ‘‘old-fashioned ways’’ and ‘‘old-fashioned values’’ still show the best way to live.”

res5: “Looking back, life was much better in the past.”

res6: “The answers for today's questions can often be found in the traditions and customs of the past.”

res7: “If you start changing things very much, you often end up making them worse.”

res8: “Changing any institution (e.g., government, religion, business) is risky, so it is better to change at a slow than a rapid pace.”

res9: “If something is wrong with an institution (e.g., government, religion, business), it is necessary to make immediate changes.”*

res10: “Our society would be better off if we showed tolerance and understanding for untraditional values and opinions.”*

---- Preference for equality ----

pfe1: “It is the responsibility of the government to take care of people who can’t take care of themselves.”

pfe2: “We need to take care of the poor and disadvantaged before helping the rest of the country.”

pfe3: “Companies should be held accountable for promoting racial diversity and a tolerant atmosphere in their offices and corporate culture.”

pfe4: “The government should take more measures to eliminate economic disparities between men and women who are doing the same work.”

pfe5: “We should find ways to help others less fortunate than ourselves.”

pfe6: “Prosperous nations have a moral obligation to share some of their wealth with poor nations.”

pfe7: “Whether we like it or not, some people are just more worthy than others.”*

---- Openness ----

o1: “I would be quite bored by a visit to an art gallery.”*

o2: “I'm interested in learning about the history and politics of other countries.”

o3: “I would enjoy creating a work of art, such as a novel, a song, or a painting.”

o4: “I think that paying attention to radical ideas is a waste of time.”*

o5: “If I had the opportunity, I would like to attend a classical music concert.”

o6: “I’ve never really enjoyed looking through an encyclopedia.”*

o7: “People have often told me that I have a good imagination.”

o8: “I like people who have unconventional views.”

o9: “I don’t think of myself as the artistic or creative type.”*

o10: “I find it boring to discuss philosophy.”*

---- Emotionality ----

e1: “I would feel afraid if I had to travel in bad weather conditions.”

e2: “I sometimes can't help worrying about little things.”

e3: “When I suffer from a painful experience, I need someone to make me feel comfortable.”

e4: “I feel like crying when I see other people crying.”

e5: “When it comes to physical danger, I am very fearful.”

e6: “I worry a lot less than most people do.”*

e7: “I can handle difficult situations without needing emotional support from anyone else.”*

e8: “I feel strong emotions when someone close to me is going away for a long time.”*

e9: “Even in an emergency I wouldn’t feel like panicking.”

e10: “I remain unemotional even in situations where most people get very sentimental.”*

---- Honesty-humility ----

hon1: “I wouldn't use flattery to get a raise or promotion at work, even if I thought it would succeed.”

hon2: “If I knew that I could never get caught, I would be willing to steal a million dollars.”*

hon3: “Having a lot of money is not especially important to me.”

hon4: “I think that I am entitled to more respect than the average person is.”*

hon5: “If I want something from someone, I will laugh at that person's worst jokes.”*

hon6: “I would never accept a bribe, even if it were very large.”

hon7: “I would get a lot of pleasure from owning expensive luxury goods.”*

hon8: “I want people to know that I am an important person of high status.”*

hon9: “I wouldn’t pretend to like someone just to get that person to do favors for me.”

hon10: “I’d be tempted to use counterfeit money, if I were sure I could get away with it.”*

---- Conscientiousness ----

c1: “I plan ahead and organize things, to avoid scrambling at the last minute.”

c2: “I often push myself very hard when trying to achieve a goal.”

c3: “When working on something, I don't pay much attention to small details.”*

c4: “I make decisions based on the feeling of the moment rather than on careful thought.”*

c5: “When working, I sometimes have difficulties due to being disorganized.”*

c6: “I do only the minimum amount of work needed to get by.”*

c7: “I always try to be accurate in my work, even at the expense of time.”

c8: “I make a lot of mistakes because I don’t think before I act.”*

c9: “People often call me a perfectionist.”

c10: ”I prefer to do whatever comes to mind, rather than stick to a plan.”*

---- Agreeableness ----

a1: “I rarely hold a grudge, even against people who have badly wronged me.”

a2: “People sometimes tell me that I am too critical of others.”*

a3: “People sometimes tell me that I'm too stubborn.”*

a4: “People think of me as someone who has a quick temper.”*

a5: “My attitude toward people who have treated me badly is ‘forgive and forget’.”

a6: “I tend to be lenient in judging other people.”

a7: “I am usually quite flexible in my opinions when people disagree with me.”

a8: “Most people tend to get angry more quickly than I do.”

a9: “Even when people make a lot of mistakes, I rarely say anything negative.”

a10: “When people tell me that I’m wrong, my first reaction is to argue with them.”*

---- Extraversion ----

ext1: “I feel reasonably satisfied with myself overall.”

ext2: “I rarely express my opinions in group meetings.”*

ext3: “I prefer jobs that involve active social interaction to those that involve working alone.”

ext4: “On most days, I feel cheerful and optimistic.”

ext5: “I feel that I am an unpopular person.”*

ext6: “In social situations, I’m usually the one who makes the first move.”

ext7: “The first thing that I always do in a new place is to make friends.”

ext8: “Most people are more upbeat and dynamic than I generally am.”*

ext9: “I sometimes feel that I am a worthless person.”*

ext10: “When I’m in a group of people, I’m often the one who speaks on behalf of the group.”

---- Single-item variables ----

LeftRight: “Where would you place yourself on the following scale of political orientation?”

Gender: 1 = Woman, 2 = Man

Age

---- Scales ----

Humanism

Normativism

RC: Resistance to change

PFE: Preference for equality

SJ: General system justification

EcoSJ: Economic system justification

Individualizing: Individualizing moral intuitions

Binding: Binding moral intuitions

Openness

Honesty

Emotionality

Extraversion

Agreeableness

Conscientiousness

1. We included two extra items for the epistemology facets of humanism and normativism, because some of the epistemology items had performed inconsistently in past studies. [↑](#footnote-ref-1)
